# Global burden of urinary tract infections in older women from 1990 to 2021 with projections to 2040: a trend analysis of the Global Burden of Disease Study 2021

**DOI:** 10.3389/fcimb.2025.1577777

**Published:** 2025-06-26

**Authors:** Yongtao Hu, Wenming Ma, Kun Tang, Qiao Qi, Wenlong Xu, Ruijie Hu, Shuchen Liu, Kaixuan Zhang, Jing Chen, Chaozhao Liang

**Affiliations:** ^1^ Department of Urology, the First Affiliated Hospital of Anhui Medical University, Hefei, China; ^2^ Institute of Urology, Anhui Medical University, Hefei, China; ^3^ Anhui Provincal Key Laboratory of Urological and Andrological Diseases Research and Medical Transformation, Anhui Medical University, Hefei, China

**Keywords:** urinary tract infection, older women, trends, joinpoint regression analysis, global burden of disease

## Abstract

**Background:**

Comprehensive estimations regarding the worldwide burden of urinary tract infections (UTIs) in older women aged ≥ 65 years are lacking. This study first explored the trends in the burden of UTIs among older women from 1990 to 2021 with projections to 2040.

**Methods:**

The Global Burden of Diseases, Injuries, and Risk Factors Study (GBD) 2021 analytical tool was adopted to calculate the age-standardized incidence rate (ASIR), age-standardized mortality rate (ASMR), and age-standardized disability-adjusted life-years (DALYs) rate (ASDR). We applied the Joinpoint regression analysis to examine the overall trends by calculating the average annual percentage change. In addition, the trends were further stratified by age group, sociodemographic index, and geographic region. Predictive analysis was employed to make further estimations of the burden until 2040.

**Results:**

The global ASIR of UTIs among older women remained stable between 1990 and 2021, while the ASMR and ASDR increased substantially during the same period. Moreover, the prediction analysis showed that although the ASIR in older women was projected to decline, the number of incident cases, deaths, and DALYs was forecasted to continue rising. Regionally, in 2021, South Asia experienced the greatest number of incident cases, deaths, and DALYs, and Tropical Latin America had the highest ASIR, ASMR, and ASDR. Between 1990 and 2021, we found Southern Latin America exhibited the largest regional-level increase in the ASIR, ASMR, and ASDR. At the national level, significant disparities in the burden of UTIs among older women were identified in different countries and territories.

**Conclusion:**

Although the ASIR of UTIs among older women has remained stable over the past three decades, the annual number of incident cases, deaths, and DALYs from UTIs has increased substantially. During the same period, a significant upward trend was found in the UTIs-related ASMR and ASDR. With population growth and aging, the burden of UTIs is expected to keep rising in the coming years. These findings can provide valuable perspectives on the distribution and magnitude relating to the burden of UTIs and promote equity in health outcomes.

## Introduction

Urinary tract infections (UTIs) are a type of microbial infection that can take place in any portion of the urinary system (Kumar et al., 2015). Among individuals suffering from UTIs, women experience the most significant disease burden (Foxman, 2010). This is attributed to a combination of factors including anatomical differences, hormonal changes, and the proximity of the urethra to the anus. Bacteria near the opening of the urethra are eliminated by urine flow; however, women have a shorter distance between the bladder and urethra compared to men. Furthermore, the urethral orifice in women is nearer to both the vagina and the rectum where large bacterial colonies exist. Consequently, bacteria are more likely to reach the bladder prior to being flushed away by urine (Hooton et al., 1999; Foxman, 2010; Kumar et al., 2015). Postmenopausal estrogen depletion increases infection susceptibility by diminishing vaginal *Lactobacillus* populations and elevating pH levels, thus compromising the natural defenses against pathogens like *Escherichia coli* (Eriksen, 1999). Therefore, women experience a higher incidence of UTIs than men, with an overall lifetime prevalence approaching 50% (Foxman, 2002).

The increasing risk of UTIs among women is significantly influenced by a variety of factors related to age. Firstly, physiological changes associated with aging, such as urethral shortening, decreased urethral sphincter tone, and reduced estrogen levels, increase the risk of bacterial colonization and subsequent infection. Secondly, older women frequently have comorbid conditions such as diabetes mellitus and neurogenic bladder dysfunction, which can further increase their susceptibility to UTIs and the likelihood of hospitalization (Neal, 1999). The use of indwelling urinary catheters in frail elderly individuals is a well-established risk factor for catheter-associated UTIs, with the risk of bacteriuria increasing by approximately 5% per day of catheterization (Werneburg, 2022). Additionally, sexual activity, constipation, urinary incontinence, and prior UTIs episodes constitute key risk factors for UTIs in older women (Rowe and Juthani-Mehta, 2013). Among community-dwelling older adults, UTIs history emerges as an independent risk predictor irrespective of age strata, with postmenopausal women demonstrating particularly heightened susceptibility to recurrent infections (Foxman et al., 2025). Unfortunately, the currently available treatments for UTIs are not considered ideal due to the increasing prevalence of multidrug-resistant (MDR) uropathogens (McLaughlin and Carson, 2004; McLellan and Hunstad, 2016).

Antimicrobial resistance (AMR) is one of the most urgent global health threats of the 21st century, and the spread of resistant strains makes the clinical management of UTIs more complicated (Gajdács et al., 2021; Patel et al., 2023). Between 1990 and 2021, the mortality related to AMR in people aged ≥ 50 years continued to rise, with an increase of 80% in those aged ≥ 70 years (GBD 2021 Antimicrobial Resistance Collaborators, 2024). *Escherichia coli*, as the main pathogen of UTIs, has developed resistance by losing or mutating porin proteins, altering penicillin-binding protein genes, and producing extended-spectrum β-lactamases (ESBLs) and carbapenemases (Darby et al., 2023). A retrospective study conducted predominantly among older women with UTIs revealed that nursing home residents aged ≥ 65 years exhibited approximately 40% greater AMR rates in *Escherichia coli*, *Proteus mirabilis*, and *Klebsiella pneumoniae* compared to their community-dwelling counterparts (Pulcini et al., 2019). Additionally, a recent longitudinal study investigating UTIs in women from 2018 to 2022 revealed a progressive decline in *Escherichia coli* antibiotic susceptibility, accompanied by a concurrent rise in resistance rates, with this concerning trend being particularly pronounced among older women (Mares et al., 2023). This trend may reflect the worsening of AMR due to increased antibiotic abuse during the COVID-19 pandemic (Rodrigues et al., 2024). AMR has created clinical challenges in the management of UTIs for older women, exacerbating therapeutic complexities and extending morbidity periods.

Due to population growth and aging, the disease burden of UTIs in older women can have significant personal, familial, and societal implications. Therefore, it is crucial to establish comprehensive disease burden indicators specifically for this population. However, at present, there is no accessible literature describing the global epidemiological burden of UTIs in older women. This study applied the data from the Global Burden of Diseases, Injuries, and Risk Factors Study (GBD) 2021 to first present the trends in the burden of UTIs among older women between 1990 and 2021. This will assist governments in formulating strategies for prevention and treatment, thereby improving overall health outcomes for this specific demographic.

## Methods

### Overview

GBD 2021 provides a thorough analysis of 371 diseases and injuries at different geographic levels. In addition to presenting the disease burden over this period, GBD also highlights disparities in health outcomes between different regions and populations. Furthermore, GBD 2021 serves as a public resource for researchers by providing detailed insights into trends in disease burden and risk factor exposure. The information can be used to guide decision-making processes aimed at improving population health around the world. Details about the methodology used in the database have been described in previous studies (GBD 2021 Diseases and Injuries Collaborators, 2024). According to the GBD database, a multitude of epidemiological studies have been conducted, offering guidance for countries in developing public health strategies and distributing healthcare resources (Jiang et al., 2023; IHME Pathogen Core Group, 2024; He et al., 2025; Yang et al., 2024).

GBD utilizes a range of interrelated metrics to assess health loss, and each metric includes an estimate and its corresponding 95% uncertainty interval (UI). Incidence refers to the number of newly diagnosed cases occurring within a defined period, while mortality represents the corresponding death count during the same timeframe. Disability-adjusted life-years (DALYs) represent a comprehensive metric that is utilized to quantify the overall health loss from non-fatal and fatal conditions. It is calculated by adding years of life lost due to premature death and years lived with disability. In the GBD study, the incidence, mortality, and DALYs are presented as crude rates per 100,000 population. The age-standardized rates are computed as weighted averages of these metrics, with the weights determined by the proportion of individuals in each corresponding age group according to the GBD standard population composition. These metrics are evaluated for 23 age groups spanning from birth to individuals aged ≥ 95 years. In addition, the estimates include data for both sexes, as well as for 204 countries and territories that could be further categorized into 21 regions (**Supplementary Table S1**). These regions consist of a group of countries that are not only geographically adjacent but also share comparable epidemiological characteristics (Murray et al., 2012). GBD regions could also be categorized by sociodemographic index (SDI) quintiles, including high, high-middle, middle, low-middle, and low SDI regions. The index is a comprehensive measure that takes into account the income per capita, average years of schooling, and total fertility rate in a given location (Foreman et al., 2018). It is presented on a scale ranging from 0 to 1. A score of 0 indicates the absolute lowest degree of development in the aspect of health, signifying a situation where various health-related indicators and conditions are at their poorest state. Conversely, a score of 1 represents the highest theoretical level achievable, indicating an ideal state where all health parameters and associated factors are at their most optimal and advanced.

### Data sources and case definition

In this study, estimates and their 95% UIs for incidence, mortality, and DALYs were extracted as measures of burden through the GBD 2021 Sources Tool (https://vizhub.healthdata.org/gbd-results/). We chose “Cause” as “UTI”, “Measure” as “Incidence, Mortality, DALYs”, “Metric” as “Number, Rate”, and “Sex” as “Female” to collect data from 204 countries and territories worldwide. The GBD 2021 database currently provides data from 1990 to 2021 for this study. To maximize data utility while maintaining temporal continuity, our analysis utilized this 32-year timeframe to align with the coverage available in the standardized GBD dataset. According to earlier publications (Mody and Juthani-Mehta, 2014; Huang et al., 2024), older women were defined as individuals aged ≥ 65 years in the study. In the GBD 2021, a hierarchical framework consisting of four levels was utilized to categorize the causes of death or injuries, ranging from the most general (Level 1; eg, Non-communicable diseases) to the most detailed (Level 4; eg, UTI). In the GBD 2021, UTI is defined as infections from the bladder and pyelum of the kidney. It is classified as a Level 4 cause within the Level 3 category of urinary diseases and male infertility, which is a subgroup of Level 2 other non-communicable diseases, all encompassed within the broader classification of Level 1 non-communicable diseases.

This study was exempt from institutional review board approval due to the publicly accessible nature of the data and the absence of personal identification. In addition, for the use of deidentified data in the GBD 2021 study, the need for informed consent was waived by the University of Washington Institutional Review Board (GBD 2021 Pulmonary Arterial Hypertension Collaborators, 2025).

### Statistical analysis

The age-standardized rate was calculated using the direct method, in which the known actual rates of all comparative data groups are standardized with reference to the standard population composition. This method allows for a more accurate comparison of disease burden across different populations by adjusting for differences in age distribution (Butler and Kalasinski, 1989; Fay and Feuer, 1997). We conducted comparative analyses on the age-standardized rate of incidence (ASIR), mortality (ASMR), and DALYs (ASDR) across various demographic categories, including age group, SDI, and geographic region. Joinpoint regression analysis was employed to assess trends in the burden of UTIs from 1990 to 2021, utilizing the average annual percentage change (AAPC) of three core indicators for quantitative evaluation. This method can detect joinpoints where the trends exhibit significant changes. The AAPC functions as a composite metric that synthesizes the weighted average of segmented annual percentage changes (APCs) derived from the Joinpoint regression model, thereby holistically characterizing long-term trends. A statistically significant increasing burden of UTIs is indicated when the lower bound of the 95% confidence interval (CI) for AAPC exceeds 0. Conversely, a decreasing trend is established when the upper bound of the 95% CI falls below 0. We calculated the AAPCs and their 95% CIs from 1990 to 2021 by age group, SDI, and geographic region. The equations for calculating the age-standardized rate and AAPC are presented in **Supplementary Figure S1**. The Bayesian age-period-cohort model, strengthened by integrated nested Laplace approximation, was utilized to predict the global burden of UTIs until 2040 (Li et al., 2023). Details about the Joinpoint regression model and Bayesian age-period-cohort model are shown in **Supplementary Figure S1**. The Nordpred model was performed as a sensitivity analysis on the prediction results (Gao et al., 2025). Statistical analyses were performed using R software (version 4.4.0) and Joinpoint Regression Program (version 4.9.1.0). Statistical significance was set at 2-sided p < 0.05.

## Results

### Global trends

**Figure 1:**
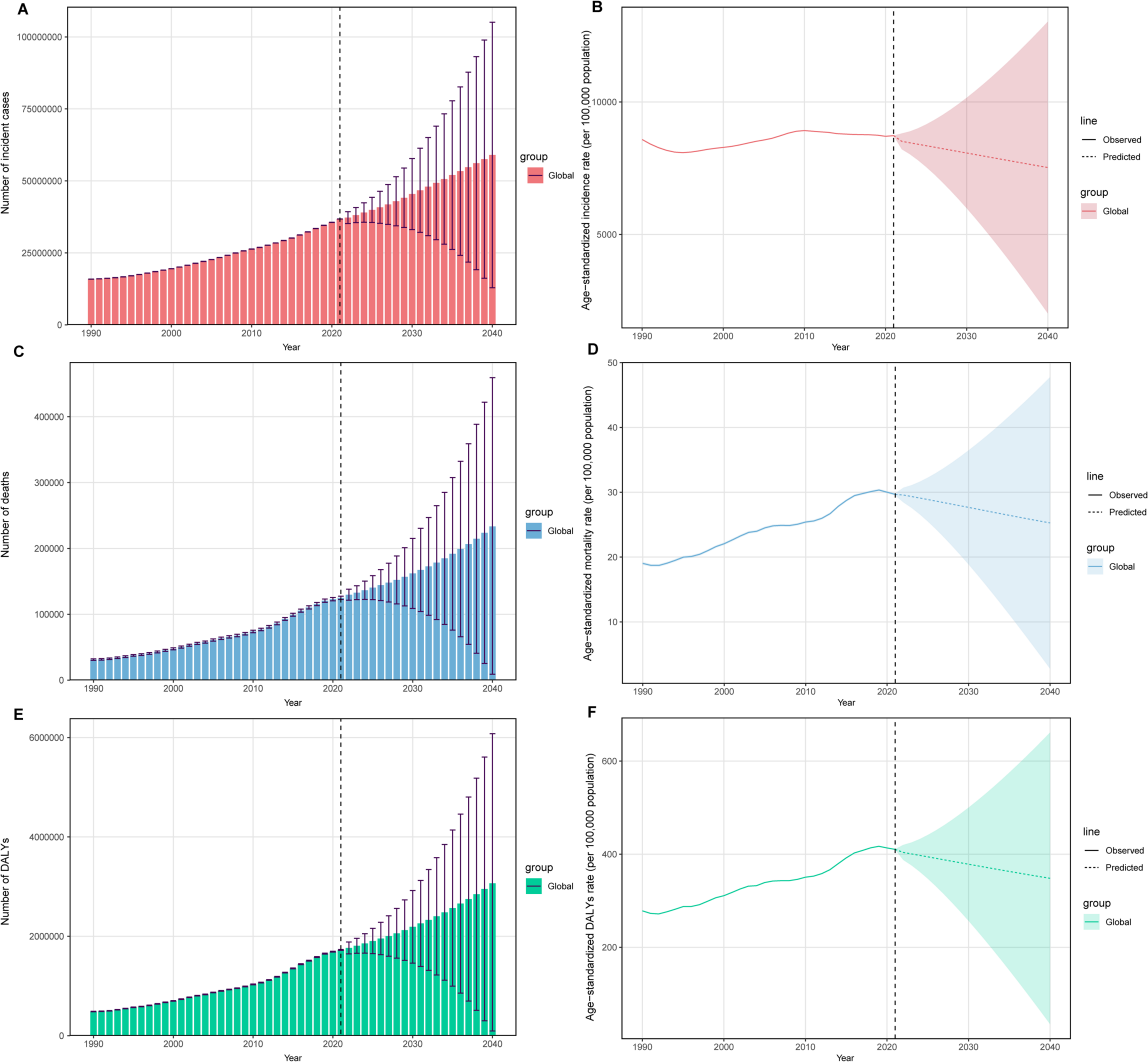
Temporal trends of the number and age-standardized incidence rate **(A, B)**, age-standardized mortality rate **(C, D)**, and age-standardized DALYs rate **(E, F)** for UTIs among older women aged ≥ 65 years from 1990 to 2040. The solid line represents the observed age-standardized rate, and the dashed line represents the age-standardized rate predicted by the Bayesian age-period-cohort model. UTIs, urinary tract infections; DALYs, disability-adjusted life-years.

Globally, the incident cases of UTIs in older women increased by 132% between 1990 and 2021, from 15.87 million (12.94-19.31) to 36.77 million (30.29-44.43). Overall, the ASIR of UTIs increased from 8580.94 (95% UI, 6985.22-10446.58) per 100,000 population in 1990 to 8727.44 (7190.01-10547.59) per 100,000 population in 2021 (**Table 1**; **Supplementary Table S2**), but the increase in the ASIR due to UTIs between 1990 and 2021 was not significant (AAPC, 0.05; 95% CI, -0.02 to 0.12; **Table 2**). During the period from 1990 to 2021, the years in which the ASIR of UTIs changed significantly were 1992, 1995, 2005, and 2009 (**Supplementary Figure S2A**). Unlike the stable trend in the ASIR, there was a notable increase in the ASMR and ASDR of UTIs among older women from 1990 to 2021 (**Supplementary Figures S2B, C**). The ASMR increased by 55.6%, from 19.06 (16.18-22.17) per 100,000 population in 1990 to 29.66 (24.18-33.28) per 100,000 population in 2021 (AAPC, 1.45; 95% CI, 1.12 to 1.77). Joinpoint regression analysis showed that the years with significant changes in the ASMR of UTIs were 1992, 2005, 2011, 2016, and 2019. While the ASDR increased by 47.4%, from 278.42 (239.85-325.44) per 100,000 population in 1990 to 410.33 (341.96-459.02) per 100,000 population in 2021 (AAPC, 1.26; 95% CI, 0.99 to 1.54). 1992, 2004, 2012, 2016, and 2019 were the years in which the ASDR of UTIs changed substantially. We also found that the continually increasing trends in the number of UTIs-related incident cases, deaths, and DALYs were noticeable between 1990 and 2021 (**Supplementary Figures S3A-C**). The prediction analysis based on the Bayesian age-period-cohort model showed that the number of incident cases from UTIs in older women was projected to continue to increase globally by 2040 (**Figure 1A**). The same trends were also found in the number of deaths and DALYs from UTIs in older women (**Figures 1C, E**). The ASIR of UTIs remained relatively stable during 1990-2021, but a downward trend was identified during the predicted period (**Figure 1B**). Furthermore, although the ASMR and ASDR for UTIs in older women have increased over the past three decades, the two indicators started to decrease after 2019, and the downward trends were predicted to continue until 2040 (**Figures 1D, F**). The analysis based on the Nordpred model also showed similar results (**Supplementary Figure S4**). However, the two models showed inconsistent results regarding the predicted trends of ASMR and ASDR. Thus, these results should be interpreted cautiously, and further research is needed to validate these conflicting outcomes.

**Table 1:** The number and age-standardized rate of incidence, mortality, and DALYs of urinary tract infections in 2021.

Location	Number of incident cases	Number of deaths	Number of DALYs	Age-standardized incidence rate per 100,000 population	Age-standardized mortality rate per 100,000 population	Age-standardized DALYs rate per 100,000 population
Global	36765312 (30285594–44432323)	124989 (101753-140289)	1725676 (1436552-1931020)	8727.44 (7190.01-10547.59)	29.66 (24.18-33.28)	410.33 (341.96-459.02)
SDI region						
High SDI	13765387 (11084856-16832029)	40179 (31183-45895)	474056 (380836-535590)	11901.82 (9593.25-14534.92)	26.99 (21.44-30.64)	348.58 (286.77-391.1)
High-middle SDI	5911031 (4888137-7060211)	23348 (19465-26050)	307823 (262972-342165)	5679.82 (4697.53-6783.2)	22.21 (18.53-24.78)	294.23 (251.61-327.01)
Middle SDI	8595804 (7062319-10323581)	32233 (26464-37519)	462752 (387774-536953)	6851.53 (5629.08-8238.61)	29.68 (24.17-34.59)	404.11 (336.33-469.28)
Low-middle SDI	6773653 (5511584-8188046)	22175 (16802-27201)	359414 (271887-440635)	10847.79 (8815.9-13128.34)	41.85 (31.75-51.37)	628.71 (476.45-770.95)
Low SDI	1686544 (1370078-2060459)	6945 (5247-8617)	120196 (91267-148292)	8371.03 (6793.06-10256.92)	43.03 (32.37-53.76)	670.73 (507.78-831.64)
Region						
Central Asia	299776 (239742-369956)	562 (470-653)	9679 (8202-11206)	8018.22 (6415.96-9887.25)	16.49 (13.78-19.17)	274.05 (231.78-317.2)
Central Europe	1330704 (1071653-1644381)	3381 (2860-3878)	45625 (39051-52766)	10044.47 (8088.62-12403.82)	23.74 (20.11-27.3)	329.72 (282.5-382.3)
Eastern Europe	2101208 (1716927-2475826)	7438 (6389-8399)	113488 (98211-128567)	9634.42 (7883.75-11337.72)	32.95 (28.36-37.16)	510.65 (443.02-577.45)
Australasia	342476 (243133-456039)	933 (693-1127)	10214 (7776-12277)	12218.76 (8640.06-16316.47)	26.04 (19.54-31.44)	303.8 (234.1-364.6)
High-income Asia Pacific	2174194 (1600347-2816000)	7398 (5011-9153)	76881 (53941-94538)	7876.58 (5907.04-10084.82)	16.32 (11.45-20.11)	194.26 (142.43-236.88)
High-income North America	8347611 (6652922-10305355)	13494 (10768-15165)	174851 (145017-194280)	23205.5 (18537.34-28619.47)	33.65 (27.23-37.67)	459.11 (385.4-508.44)
Southern Latin America	639089 (506974-780562)	4489 (3671-5196)	55896 (46492-64426)	13724.86 (10886.72-16749.46)	85.74 (70.4-99.2)	1105.39 (923.56-1272.86)
Western Europe	2876150 (2272981-3493594)	20728 (15949-23793)	227522 (179106-260015)	5971.6 (4732.09-7220.42)	28.19 (22.08-32.26)	339.33 (273.26-386.07)
Andean Latin America	412487 (322271-511472)	1189 (770-1685)	16090 (10391-22710)	15505.67 (12114.84-19228.46)	44.33 (28.7-62.82)	602.67 (389.16-850.57)
Caribbean	212487 (170269-262945)	627 (509-751)	8237 (6795-9818)	8322.11 (6676.06-10290.45)	22.15 (18.12-26.4)	303.01 (251.42-360.36)
Central Latin America	1321783 (1081463-1602406)	5398 (4596-6216)	81391 (69788-94324)	11323.61 (9268.45-13731.21)	47.02 (40.09-54.07)	705.27 (605.22-816.3)
Tropical Latin America	2177417 (1780511-2626699)	14217 (11389-16119)	185148 (153136-207484)	17297.78 (14150.66-20866.7)	110.51 (88.9-125.1)	1455.58 (1207.28-1629.42)
North Africa and Middle East	613706 (495988-751408)	3047 (2312-3908)	43478 (33274-55756)	3479.51 (2814.97-4261.91)	22.43 (16.91-28.76)	294.69 (224.21-377.8)
South Asia	8626969 (7053044-10478189)	23903 (17815-29811)	402290 (299966-499526)	13419.06 (10961.14-16321.66)	43.76 (32.63-54.81)	681.45 (508.59-848.46)
East Asia	2556786 (2029403-3124327)	4723 (3509-6846)	65647 (49435-97094)	2305.79 (1827.03-2822.56)	5.12 (3.78-7.34)	67.05 (50.29-98.22)
Oceania	8894 (7004-11187)	27 (17-46)	450 (288-779)	3594.48 (2824.88-4522.33)	15.12 (9.7-25.44)	217.56 (139.58-371.66)
Southeast Asia	1683440 (1399487-2040082)	8417 (5683-11281)	122779 (85286-161620)	5712.97 (4748.78-6923.35)	35.19 (23.4-47.64)	478.03 (328-634.66)
Central Sub-Saharan Africa	100311 (79587-125430)	256 (133-550)	4426 (2329-9420)	4655.49 (3693.55-5823.91)	16 (8.27-34.27)	242.88 (127.27-517.49)
Eastern Sub-Saharan Africa	356108 (288225-437408)	3626 (2802-4682)	63233 (49225-81472)	5298.85 (4287.52-6510.28)	65.42 (49.99-84.93)	1039.71 (803.58-1343.47)
Southern Sub-Saharan Africa	105213 (83830-130938)	202 (108-341)	3096 (1717-5156)	3694.55 (2943.66-4593.85)	8.78 (4.66-14.95)	124.46 (68.17-208.64)
Western Sub-Saharan Africa	478505 (385266-592718)	935 (681-1489)	15256 (11165-24013)	6308.78 (5080.23-7819.71)	16.08 (11.64-25.84)	237.16 (173.15-375.2)

DALYs, disability-adjusted life-years; SDI, sociodemographic index.

**Table 2:** AAPCs in the age-standardized rate of incidence, mortality, and DALYs of urinary tract infections from 1990 to 2021 at the global and regional levels.

Location	Incidence		Mortality		DALYs	
	AAPC, 1990-2021	p value	AAPC, 1990-2021	p value	AAPC, 1990-2021	p value
Global	0.05 (-0.02 to 0.12)	0.192	1.45 (1.12 to 1.77)	< 0.001	1.26 (0.99 to 1.54)	< 0.001
SDI region						
High SDI	0.09 (-0.04 to 0.22)	0.177	0.87 (0.5 to 1.25)	< 0.001	0.74 (0.38 to 1.1)	< 0.001
High-middle SDI	-0.39 (-0.43 to -0.36)	< 0.001	2.06 (1.65 to 2.48)	< 0.001	1.44 (1.04 to 1.85)	< 0.001
Middle SDI	0.58 (0.55 to 0.6)	< 0.001	2.18 (1.93 to 2.44)	< 0.001	2 (1.79 to 2.22)	< 0.001
Low-middle SDI	0.47 (0.42 to 0.52)	< 0.001	1.61 (1.15 to 2.08)	< 0.001	1.36 (1 to 1.73)	< 0.001
Low SDI	0.45 (0.43 to 0.47)	< 0.001	0.66 (0.39 to 0.93)	< 0.001	0.49 (0.18 to 0.79)	0.002
Region						
Central Asia	0.26 (0.2 to 0.32)	< 0.001	1.9 (0.65 to 3.18)	0.003	1.66 (0.38 to 2.95)	0.011
Central Europe	-0.02 (-0.04 to -0.00)	0.016	0.44 (-0.55 to 1.44)	0.387	0.04 (-0.55 to 0.63)	0.894
Eastern Europe	0.17 (0.13 to 0.21)	< 0.001	2.93 (1.6 to 4.28)	< 0.001	2.34 (1.56 to 3.12)	< 0.001
Australasia	-0.02 (-0.16 to 0.11)	0.741	1.37 (-0.23 to 2.99)	0.093	0.88 (-0.76 to 2.54)	0.296
High-income Asia Pacific	0.35 (0.27 to 0.44)	< 0.001	1.11 (0.44 to 1.79)	0.001	0.8 (0.19 to 1.42)	0.011
High-income North America	0.34 (0.08 to 0.61)	0.01	0.32 (-0.22 to 0.87)	0.241	0.4 (-0.11 to 0.91)	0.123
Southern Latin America	0.75 (0.71 to 0.79)	< 0.001	4.62 (3.43 to 5.83)	< 0.001	4.4 (3.22 to 5.6)	< 0.001
Western Europe	-0.22 (-0.27 to -0.16)	< 0.001	2.4 (1.96 to 2.85)	< 0.001	2.08 (1.64 to 2.52)	< 0.001
Andean Latin America	0.47 (0.41 to 0.52)	< 0.001	1.59 (0.79 to 2.39)	< 0.001	1.63 (0.83 to 2.43)	< 0.001
Caribbean	0.05 (0.03 to 0.06)	< 0.001	2.91 (2.01 to 3.82)	< 0.001	2.83 (1.99 to 3.67)	< 0.001
Central Latin America	0.59 (0.43 to 0.75)	< 0.001	2.16 (1.76 to 2.56)	< 0.001	2.39 (2.04 to 2.74)	< 0.001
Tropical Latin America	0.45 (0.39 to 0.52)	< 0.001	4.57 (3.83 to 5.31)	< 0.001	4.29 (3.57 to 5.01)	< 0.001
North Africa and Middle East	0.23 (0.2 to 0.25)	< 0.001	0.83 (0.61 to 1.05)	< 0.001	0.68 (0.52 to 0.85)	< 0.001
South Asia	0.42 (0.38 to 0.45)	< 0.001	1.09 (0.28 to 1.9)	0.008	0.9 (0.38 to 1.41)	0.001
East Asia	0.04 (0.02 to 0.06)	< 0.001	-0.85 (-1.48 to -0.23)	0.008	-1.18 (-1.74 to -0.61)	< 0.001
Oceania	0.14 (0.13 to 0.14)	< 0.001	-0.53 (-0.91 to -0.15)	0.006	-0.44 (-0.84 to -0.03)	0.034
Southeast Asia	-0.02 (-0.07 to 0.03)	0.481	2 (1.87 to 2.14)	< 0.001	1.62 (1.51 to 1.74)	< 0.001
Central Sub-Saharan Africa	0.16 (0.15 to 0.18)	< 0.001	0.1 (0.05 to 0.15)	< 0.001	0.04 (-0.01 to 0.1)	0.108
Eastern Sub-Saharan Africa	0.17 (0.15 to 0.19)	< 0.001	0.37 (0.18 to 0.55)	< 0.001	0.18 (0.04 to 0.31)	0.01
Southern Sub-Saharan Africa	-0.12 (-0.31 to 0.07)	0.229	0.83 (0.46 to 1.19)	< 0.001	0.93 (0.56 to 1.3)	< 0.001
Western Sub-Saharan Africa	0.11 (0.08 to 0.14)	< 0.001	0.23 (0.13 to 0.33)	< 0.001	0.17 (0.11 to 0.24)	< 0.001

AAPC, average annual percentage change; DALYs, disability-adjusted life-years; SDI, sociodemographic index.

With regard to the incidence of UTIs during 1990-2021, the most pronounced decrease was observed in older women aged ≥ 95 years (AAPC, -0.4; 95% CI, -0.57 to -0.23; **Supplementary Table S3**). We found that the incidence of UTIs in older women aged ≥ 80 years showed a declining trend after 2010 (**Supplementary Figure S5A**), which may be influenced by multifactorial drivers, including health management and education programs promoting healthier lifestyles, precision-based antibiotic stewardship strategies mitigating the risk of recurrence, and updated medical guidelines influencing subsequent screening and diagnostic practices (Nicolle et al., 2005; Marques et al., 2012; Linhares et al., 2013). The mortality and DALYs presented contrasting patterns, with a more significant rise observed in older age groups (**Supplementary Figures S5B, C**). The mortality in older women aged ≥ 95 years increased from 160.7 (119.53-186.66) per 100,000 population in 1990 to 305.89 (213.2-356.28) per 100,000 population in 2021 (AAPC, 2.22; 95% CI, 1.79 to 2.66). In addition, among older women aged ≥ 95 years, the DALYs increased from 1308.81 (975.47-1518.48) per 100,000 population in 1990 to 2460.44 (1718.93-2862.74) per 100,000 population in 2021 (AAPC, 2.18; 95% CI, 1.74 to 2.61). In 2021, the burden of UTIs among older women demonstrated marked age-related disparities, with those aged ≥ 90 years exhibiting significantly higher incidence, mortality, and DALYs compared to younger age groups. We also found that the incidence of UTIs in women aged ≥ 80 years tended to increase with age group. In 2021, the ASIR was highest in the high SDI region while the highest ASMR and ASDR were observed in the low SDI region (**Supplementary Figures S6A-C**). The ASIR, ASMR, and ASDR of UTIs significantly increased across all sociodemographic subgroups, except for the unchanged trend of ASIR in the high SDI region, and the downward trend of ASIR in the high-middle SDI region (**Table 2**). Overall, the case number of the three indicators due to UTIs increased, with the highest number in the high SDI region in 2021 (**Table 1**).

### Regional trends

Regionally, in 2021, High-income North America experienced the largest ASIR for older women with UTIs (23205.5 [18537.34-28619.47] per 100,000 population; **Table 1**), and Tropical Latin America showed the largest ASMR (110.51 [88.9-125.1] per 100,000 population) and the largest ASDR (1455.58 [1207.28-1629.42] per 100,000 population). Southern Latin America had the most rapid increase in the ASIR (from 10888.24 [8416.65-13807.91] in 1990 to 13724.86 [10886.72-16749.46] in 2021 per 100,000 population; AAPC, 0.75; 95% CI, 0.71 to 0.79; **Table 2**; **Supplementary Table S4**), the ASMR (from 19.13 [16.29-21.7] in 1990 to 85.74 [70.4-99.2] in 2021 per 100,000 population; AAPC, 4.62; 95% CI, 3.43 to 5.83), and the ASDR (from 263.67 [227.21-297.99] in 1990 to 1105.39 [923.56-1272.86] in 2021 per 100,000 population; AAPC, 4.4; 95% CI, 3.22 to 5.6). Despite increases in the ASIR, ASMR, and ASDR across the majority of regions between 1990 and 2021, some regions showed downward trends. From 1990 to 2021, Western Europe exhibited the most significant reduction in the ASIR (from 6400.15 [5226.42-7753.56] in 1990 to 5971.6 [4732.09-7220.42] in 2021 per 100,000 population; AAPC, -0.22; 95% CI, -0.27 to -0.16), while East Asia experienced the most notable decrease in the ASMR (from 6.69 [4.32-9.16] in 1990 to 5.12 [3.78-7.34] in 2021 per 100,000 population; AAPC, -0.85; 95% CI, -1.48 to -0.23), and the ASDR (from 96.66 [63.2-131.34] in 1990 to 67.05 [50.29-98.22] in 2021 per 100,000 population; AAPC, -1.18; 95% CI, -1.74 to -0.61). Among all GBD regions, the greatest number of incident cases, deaths, and DALYs for UTIs among older women aged ≥ 65 years occurred in South Asia in 2021.

### National trends

**Figure 2:**
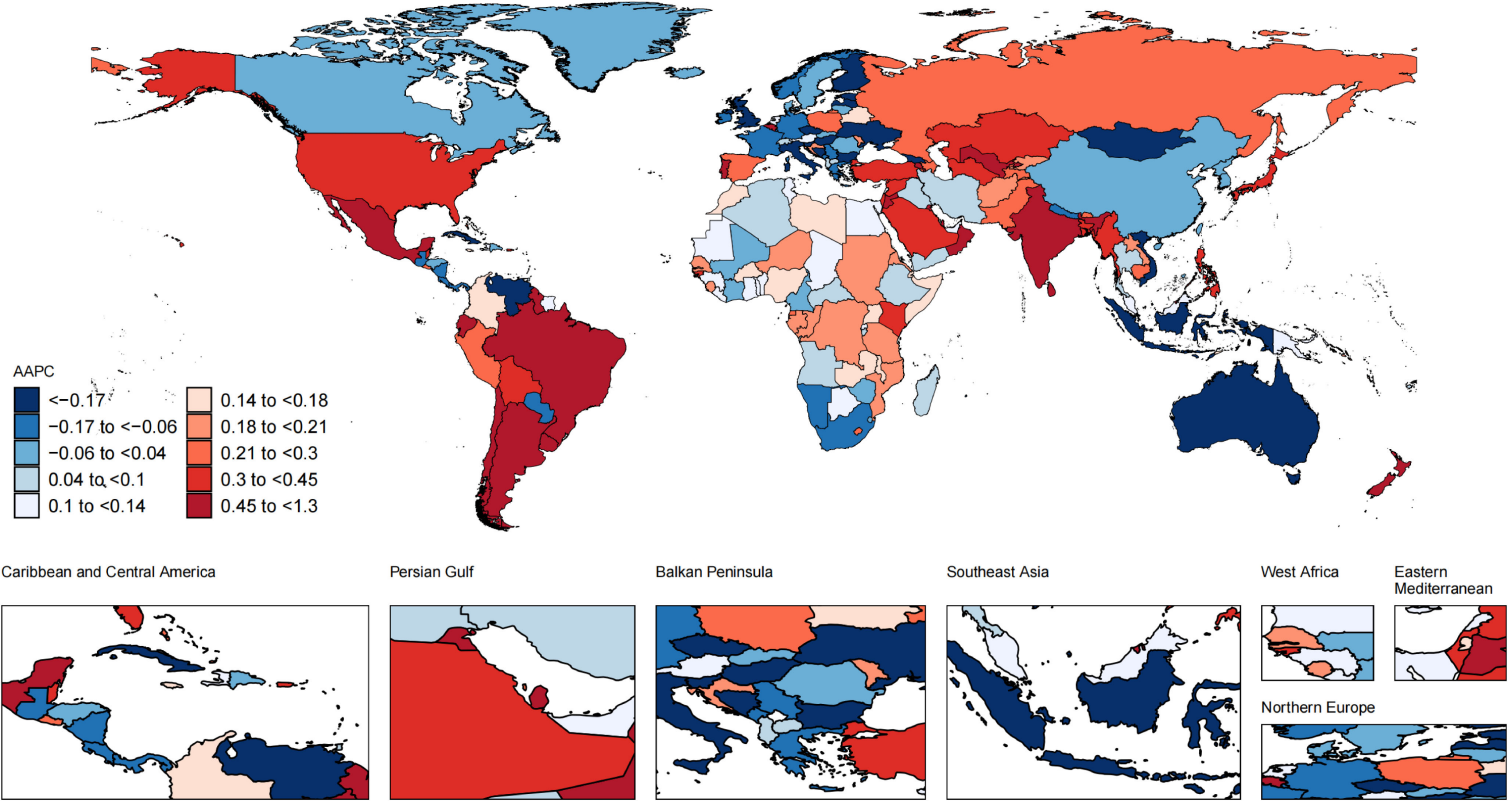
Global map of average annual percentage change in the age-standardized incidence rate of UTIs among older women aged ≥ 65 years from 1990 to 2021. AAPC, average annual percentage change; UTIs, urinary tract infections.

**Figure 3:**
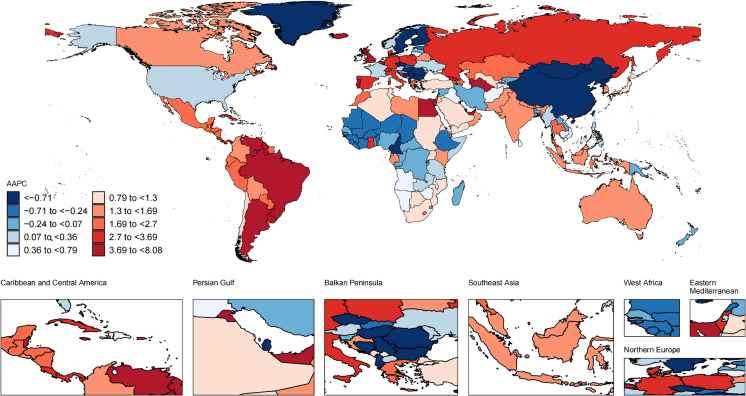
Global map of average annual percentage change in the age-standardized mortality rate of UTIs among older women aged ≥ 65 years from 1990 to 2021. AAPC, average annual percentage change; UTIs, urinary tract infections.

**Figure 4:**
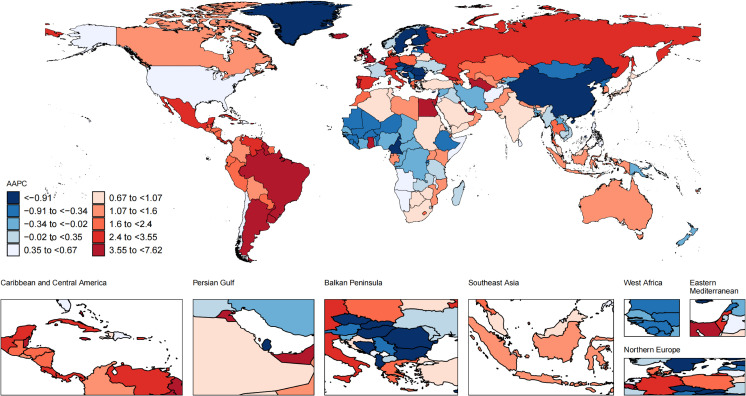
Global map of average annual percentage change in the age-standardized DALYs rate of UTIs among older women aged ≥ 65 years from 1990 to 2021. AAPC, average annual percentage change; UTIs, urinary tract infections; DALYs, disability-adjusted life-years.

**Figure 5:**
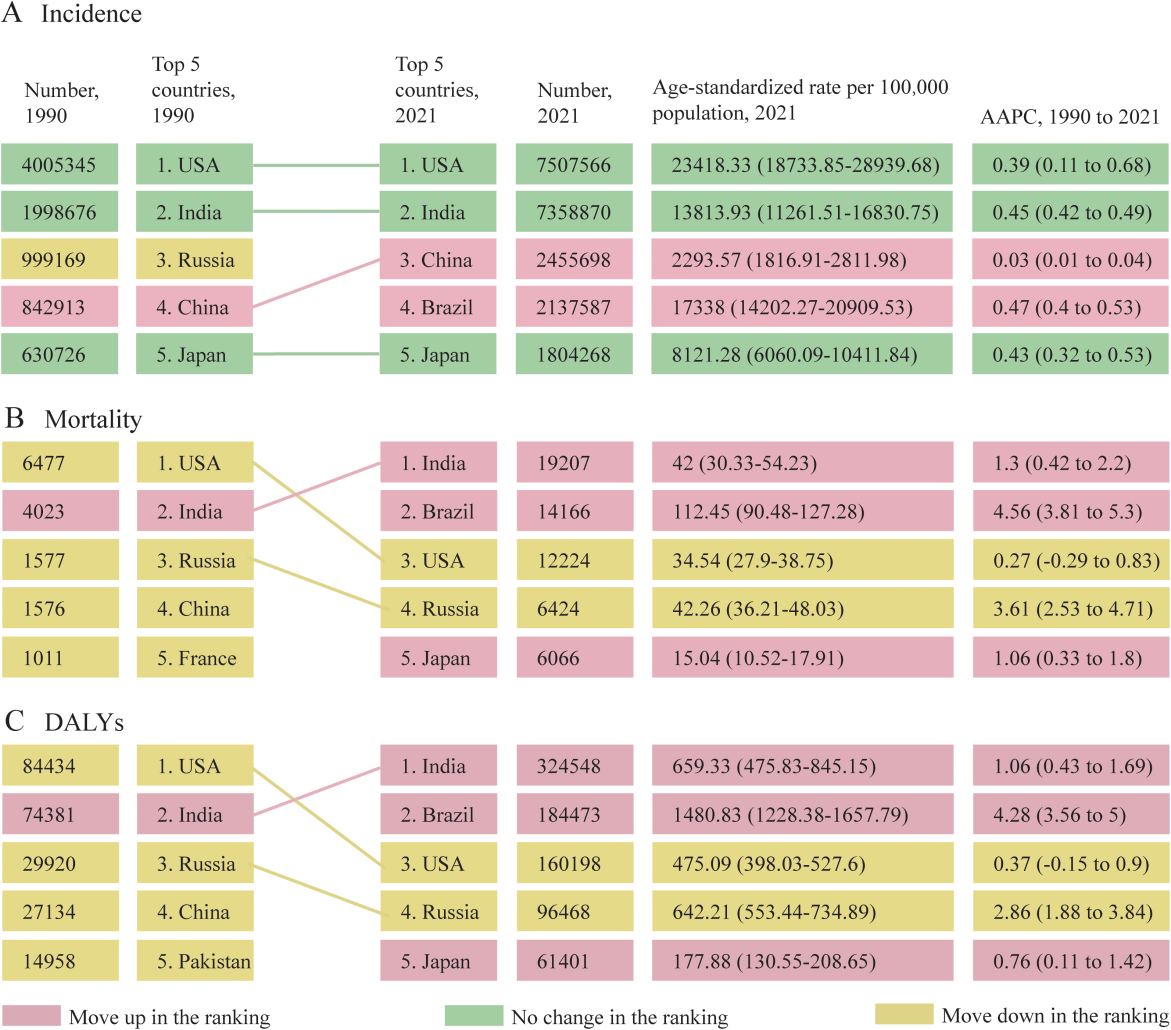
Changes in the top 5 countries with the highest absolute burden of UTIs among older women aged ≥ 65 years from 1990 to 2021, based on the number of incident cases **(A)**, deaths **(B)**, and DALYs **(C)**. AAPC, average annual percentage change; UTIs, urinary tract infections; DALYs, disability-adjusted life-years.

Nationally, from 1990 to 2021, the most substantial increase in the ASIR of UTIs occurred in New Zealand (from 10640.08 [7732.6-14035.03] in 1990 to 16009.17 [11754.29-20739.92] in 2021 per 100,000 population; AAPC, 1.3; 95% CI, 1.22 to 1.38; **Figure 2**; **Supplementary Table S5**), while United Kingdom had the sharpest decline in the ASIR of UTIs (from 5737.96 [4643.54-7025.68] in 1990 to 3926.78 [3171.41-4843.91] in 2021 per 100,000 population; AAPC, -1.18; 95% CI, -1.33 to -1.02). Over the same period, Kuwait exhibited the most significant increase in the ASMR (from 1.86 [1.38-2.32] in 1990 to 23.84 [15.58-31.96] in 2021 per 100,000 population; AAPC, 8.08; 95% CI, 3.21 to 13.18; **Figure 3**; **Supplementary Table S6**), and the ASDR (from 28.76 [21.76-35.88] in 1990 to 316.21 [215.14-417.46] in 2021 per 100,000 population; AAPC, 7.62; 95% CI, 2.93 to 12.52; **Figure 4**; **Supplementary Table S7**). In contrast to the upward trends in Kuwait, during the period from 1990 to 2021, Guam had the substantial decreases in the ASMR (from 80.92 [48.63-123.74] in 1990 to 15.41 [9.37-23.23] in 2021 per 100,000 population; AAPC, -5.59; 95% CI, -8.05 to -3.08), and the ASDR (from 982.55 [602.99-1494.2] in 1990 to 260.02 [165.06-383.63] in 2021 per 100,000 population; AAPC, -4.52; 95% CI, -6.46 to -2.54). In 2021, the United States of America had the largest number of incident cases and the highest ASIR of UTIs among older women aged ≥ 65 years (**Figure 5A**). The country with the largest number of UTIs-related deaths and DALYs in 2021 was India (**Figures 5B, C**). In 2021, the United Arab Emirates experienced the highest ASMR (113.53 [59.05-181.16] per 100,000 population) and ASDR (1641.82 [875.37-2595.58] per 100,000 population). The age-standardized rates of the three indicators among older women aged ≥ 65 years in 1990 and 2021 are shown in **Supplementary Figures S7, 8**.

## Discussion

To our knowledge, this study is the first comprehensive analysis reporting the trends in the burden of UTIs among older women aged ≥ 65 years at different geographic levels from 1990 to 2021. Between 1990 and 2021, a notable rise was observed in the annual number of incident cases related to UTIs, along with related deaths and DALYs among older women. Another epidemiological study focusing on the burden of UTIs in the Middle East and North Africa region showed similar results (Amiri et al., 2025). This occurred despite the fact that the ASIR was relatively stable over the same period. Additionally, upward trends were also identified in the UTIs-related ASMR and ASDR. The prediction analysis indicated that despite the projected decrease in the ASIR for UTIs in older women, the number of incident cases, deaths, and DALYs was predicted to keep increasing. Therefore, it is imperative to develop effective treatment and prevention strategies aimed at addressing the heavy burden and mitigating the adverse effects of UTIs in this population. This study serves as a valuable resource for national healthcare policymakers to evaluate the burden of UTIs, enabling them to inform the development of such strategies.

Our study showed that the incidence of UTIs in older women tended to increase with the elevation of age groups after the age of 80, which was consistent with the findings of previous studies (Ahmed et al., 2018). A large-scale retrospective study analyzed the incidence of clinically diagnosed UTIs among older adults in the UK from March 2004 to April 2014. The results indicated that for older women, the incidence of clinically diagnosed UTIs per 100 person-years at risk rose from 11.35 to 14.34 in the 75–84 age group and from 14.65 to 19.8 in those aged 85 and over. However, it was interesting to note that since 2010, a downward trend in the incidence of UTIs was found in older women over 80 years, and this decrease was more significant in higher age groups, while it plateaued in older women under 80 years, which is contrary to our understanding. With the progress in medical technology and the wide availability of healthcare services, the diagnosis and treatment of UTIs have been significantly enhanced. Clinical investigations have demonstrated that intravesical instillation of hyaluronic acid-chondroitin sulfate combination therapy significantly reduces the incidence of UTIs while maintaining a favorable safety profile. This therapeutic regimen not only alleviates clinical symptoms but also enhances quality of life metrics in patients with recurrent UTIs, as evidenced by sustained therapeutic benefits observed throughout a 12-month follow-up period (Damiano et al., 2011). Current clinical advancements have empowered healthcare professionals to achieve timely detection of UTIs among elderly patients, leading to more precise antibiotic treatment strategies that effectively prevent recurrence (Linhares et al., 2013). In addition, healthcare systems are placing increased emphasis on health management and education for the elderly population. The protocol promotes healthier lifestyle habits among older women, contributing to measurable reductions in UTIs occurrence among this demographic (Marques et al., 2012). The guidelines from the Infectious Diseases Society of America do not recommend routine screening and therapeutic interventions for this condition across community settings (Nicolle et al., 2005). These guidelines are expected to gradually change subsequent screening and diagnostic practices for UTIs in older women, potentially helping reduce the incidence over time.

From 1990 to 2021, the ASIR in the high SDI region remained stable, while that in the high-middle SDI region declined. Conversely, in other regions, the ASIR, ASMR, and ASDR all rose. In addition, the region with high SDI exhibited the highest ASIR, whereas the region with low SDI showed the greatest ASMR and ASDR. The study of Zhu et al. also showed that the ASIR of UTIs was the highest in high SDI regions, while the ASDR and ASMR of UTIs were the highest in low-middle SDI regions, which was slightly different from our results (Zhu et al., 2021). Therefore, prioritized interventions to mitigate the burden of UTIs should focus on the low-middle SDI region globally, with particular emphasis on low SDI regions when addressing elderly female populations. The heterogeneous burden of UTIs across SDI groups arises synergistically from disparities in healthcare access, socioeconomic status, aging demographics, and antibiotic misuse. High SDI regions, equipped with better healthcare infrastructure and advanced medical technologies, can detect UTIs early, leading to a higher ASIR. However, in low SDI regions, limited healthcare resources may result in delayed diagnosis and inadequate treatment, which could contribute to higher ASMR and ASDR as patients develop severe complications by the time they receive medical attention (Öztürk and Murt, 2020). Socioeconomic status also plays a crucial role in health outcomes. In low SDI regions, low-quality healthcare systems, poor living conditions, and inadequate sanitation due to socioeconomic disparities can increase the risk of UTIs. Malnutrition and lack of clean water may further weaken immunity. High SDI regions have better hygiene and advanced healthcare systems, which help to reduce severe UTIs and mortality (Rosenthal et al., 2025). In addition, aging demographics differ across SDI regions. Low SDI regions have a relatively younger population, but their elderly face more healthcare access challenges. High SDI regions have more elderly people, explaining the higher ASIR as UTIs incidence rises with age. Their better healthcare systems can effectively manage the disease, preventing severe stages and leading to lower ASMR and ASDR (Zeng et al., 2022). Lastly, antibiotic use patterns vary significantly between regions. High SDI regions have stricter antibiotic regulations and greater awareness of resistance, promoting appropriate use. In contrast, low SDI regions may have more antibiotic overuse and misuse due to self-medication and insufficient regulation. This contributes to antibiotic resistance, making UTIs harder to treat and increasing complications and mortality risks (Sachdev et al., 2022). The high burden of UTIs among older women in low SDI regions highlights an urgent need for healthcare systems to implement targeted interventions. Firstly, implementing regular screening programs can facilitate early detection and treatment of UTIs, particularly in high-risk elderly populations. Secondly, enhancing infection control measures in long-term care facilities is crucial; this includes improving hygiene practices, establishing strict sterilization protocols for medical equipment, and conducting regular infection surveillance (Prieto et al., 2025). Thirdly, focusing on geriatric urology can lead to better prevention and management strategies tailored to the unique needs of older women. Additionally, strengthening the community-based health education, ensuring the rational use of antibiotics and monitoring antibiotic resistance, establishing robust UTIs surveillance and follow-up systems will assist in monitoring and managing the disease (Prieto et al., 2024).

South Asia experienced the highest burden of UTIs among older women in 2021. This challenging situation could be intimately linked to the sheer size of the population in India and similar countries within South Asia. The study by Zhu et al. also confirmed the high burden of UTIs in South Asia. These findings show that South Asia has not only a heavy disease burden of UTIs among older women but also the highest overall burden of UTIs globally (Zhu et al., 2021). High-income North America had a higher ASIR, whereas Tropical Latin America had higher ASMR and ASDR, which is in line with the results of another global epidemiological study of UTIs (Zeng et al., 2022). The most significant rises in the ASIR, ASMR, and ASDR occurred in Southern Latin America from 1990 to 2021, which might be associated with bacterial resistance to antimicrobial agents. Moreover, there is a high mortality across all age groups in Latin America due to bacterial resistance against antimicrobial agents (Antimicrobial Resistance Collaborators, 2022; Li et al., 2022). The country with the largest number of UTIs-related deaths and DALYs in 2021 was India. Epidemiological analyses by Zeng et al. demonstrated significant alignment with these findings, a phenomenon which is likely attributable to the demographic characteristics of India, notably its expansive population base (Zeng et al., 2022). The United Arab Emirates had the highest ASMR and ASDR in 2021. ESBL-producing bacteria have emerged as the leading cause of community-acquired UTIs in the United Arab Emirates, while ESBL-producing bacteria are considered MDR pathogens with a limited range of treatment options, leading to increased morbidity and mortality (Schwaber et al., 2005; Schwaber and Carmeli, 2007; Ranjan Dash et al., 2018). The ASIR had the most significant increase in New Zealand from 1990 to 2021. *Escherichia coli* and *Klebsiella pneumoniae* are common causes of MDR UTIs in the New Zealand community, with increasing rates from 2011 to 2016, which may have led to the increase in the ASIR (Toombs-Ruane et al., 2020). During this period, Kuwait experienced the most significant rises in the ASMR and ASDR. A study from Kuwait revealed that ESBL-producing *Escherichia coli* and *Klebsiella pneumoniae* had a significant role in the pathogenesis of hospital- and community-acquired UTIs. A considerable proportion of these pathogens in Kuwait were highly resistant to first- and second-line antibiotics for UTIs, which may have accounted for the substantial increases in recent years (Al Benwan et al., 2010). Portugal has the second-most pronounced increase in ASMR after Kuwait, while simultaneously confronting MDR uropathogens that compromise the management of UTIs in older women (Linhares et al., 2013). Furthermore, inappropriate antibiotic utilization and indiscriminate catheter application in older women have emerged as compounding factors, exacerbating mortality outcomes through their association with AMR development and iatrogenic complications (Almeida et al., 2020; Rodrigues et al., 2025). These findings emphasize implementing targeted infection control, antimicrobial stewardship, and real-time surveillance to optimize UTIs outcomes and mitigate resistance evolution (Gharbi et al., 2019).

The increasing burden of UTIs in older women is significantly exacerbated by the emergence of MDR pathogens, particularly *Escherichia coli* and *Klebsiella pneumoniae*. *Escherichia coli* is the most common causative pathogen of UTIs, accounting for approximately 70% of cases. Among these, MDR strains are on the rise. Studies have shown that MDR *Escherichia coli* exhibits high resistance rates to common antibiotics such as ceftriaxone, ampicillin, and cefotaxime, with resistance rates exceeding 50% in some regions (Jalil and Al Atbee, 2022). *Klebsiella pneumoniae* is another common pathogen in UTIs, particularly in hospital-acquired UTIs. MDR *Klebsiella pneumoniae* strains are also on the rise, exhibiting resistance to multiple antibiotics, including carbapenems. ESBL-producing *Klebsiella pneumoniae* strains are highly resistant to third-generation cephalosporins and may also display resistance to other classes of antibiotics such as aminoglycosides and fluoroquinolones (Ciresa et al., 2024). Infections caused by these MDR strains are associated with higher treatment failure rates, prolonged hospital stays, and increased mortality. For instance, a study found that patients with UTIs caused by carbapenem-resistant *Klebsiella pneumoniae* had a mortality rate nearly twice that of patients infected with carbapenem-susceptible strains. Inappropriate empirical antibiotic therapy and inadequate antibiotic stewardship programs contribute to the development of MDR strains (Rodrigues et al., 2024). To address this challenge, the World Health Organization has listed carbapenem-resistant *Escherichia coli* and *Klebsiella pneumoniae* as critically priority pathogens (Cabrera et al., 2025). Rapid diagnostic technologies and precision medicine approaches are needed to guide antibiotic use, coupled with enhanced infection control measures to curb the spread of MDR pathogens, thereby improving UTIs outcomes in older women.

Our study has several limitations. Firstly, the GBD database does not differentiate between acute and recurrent UTIs in its publicly available datasets. This lack of distinction may obscure nuanced epidemiological patterns and limit our ability to fully capture the distinct disease burdens and trends of these two clinically different entities. As a result, the findings should be interpreted with caution, especially when considering the specific implications for clinical management and public health strategies related to acute versus recurrent UTIs. Future iterations of the GBD and similar databases could adopt a more granular categorization of UTIs, allowing for more detailed and accurate analyses of their respective burdens and trends over time. Secondly, the absence of specific pathogen data limits our understanding of the relationship between these pathogens and the burden of UTIs in older women. This gap highlights the critical need for future global health studies to integrate pathogen-specific data, which would enable more nuanced analyses of infection dynamics and targeted intervention strategies. Thirdly, our analysis did not account for the impact of AMR on UTIs outcomes. Variations in AMR prevalence across countries, particularly the lack of systematic AMR surveillance data in low-resource settings, may have led to underestimation of mortality and disability rates in regions with high antibiotic resistance. Future studies integrating AMR surveillance data could refine burden estimates and improve the accuracy of predictive models. Finally, in regions where diagnostic procedures are inconsistent or healthcare services are limited, the data may be subject to biases that the Joinpoint regression and Bayesian age-period-cohort models cannot fully account for. While the GBD 2021 version ensures methodological consistency for historical estimates, this could potentially influence the accuracy of the identified trends and projections, as the models rely on the premise of relatively uniform data quality across different regions. This limitation should be considered when interpreting the results, especially when extrapolating findings to regions with markedly different healthcare contexts than those represented in the dataset.

## Conclusion

Despite the ASIR among older women with UTIs remaining stable from 1990 to 2021, the UTIs-related ASMR and ASDR have increased significantly over the past three decades. Furthermore, our study showed that the burden of UTIs in terms of absolute numbers would continue to increase globally by 2040 despite the predicted downward trend in ASIR. The significant impact of UTIs on older women in diverse SDI regions highlights the immediate necessity for healthcare systems to implement tailored interventions. High SDI regions should prioritize strengthening early diagnosis systems and personalized interventions to address high ASIR; low SDI regions require expanding antibiotic access and community health worker training to mitigate ASMR and ASDR, while high-middle SDI regions should sustain declining ASIR by scaling proven prevention strategies. Future prospective cohort studies are essential to validate these findings and projections.

## Data Availability

Publicly available datasets were analyzed in this study. This data can be found here: https://vizhub.healthdata.org/gbd-results/.
