# Supplementary material for: Global burden of urinary tract infections in older women from 1990 to 2021 with projections to 2040: a trend analysis of the Global Burden of Disease Study 2021
Source: Front Cell Infect Microbiol. 2025 Jun 26;15:1577777. doi: 10.3389/fcimb.2025.1577777 (PMC12240952; doi:10.3389/fcimb.2025.1577777)
Supplement: Supplementary file 1 [file Table1.docx]

**Supplementary Materials**

**Catalogue**

**Figure S1**. Supplementary methods **3**

**Figure S2**. The Joinpoint regression analysis on the age-standardized incidence (A), mortality (B), and DALYs (C) rates of urinary tract infections among older women from 1990 to 2021 at the global level **4**

**Figure S3**. The Joinpoint regression analysis on the number of incident cases (A), deaths (B), and DALYs (C) of urinary tract infections among older women from 1990 to 2021 at the global level **5**

**Figure S4**. Temporal trends of the number and age-standardized incidence rate (A, B), age-standardized mortality rate (C, D), and age-standardized DALYs rate (E, F) for urinary tract infections among older women aged ≥ 65 years from 1990 to 2040 **6**

**Figure S5**. The line charts of the incidence (A), mortality (B), and DALYs (C) of urinary tract infections among older women from 1990 to 2021 at the global level in subgroup of age **7**

**Figure S6**. The line charts of the age-standardized incidence (A), mortality (B), and DALYs (C) rates of urinary tract infections among older women from 1990 to 2021 at the global level in subgroup of SDI **8**

**Figure S7**. Global map of the age-standardized rate of global incidence (A), mortality (B), and DALYs (C) of urinary tract infections among older women in 1990. **9**

**Figure S8**. Global map of the age-standardized rate of global incidence (A), mortality (B), and DALYs (C) of urinary tract infections among older women in 2021. **10**

**Table S1**. Geographical hierarchy and SDI quintiles for countries estimated in GBD 2021 **11**

**Table S2**. The age-standardized rate of incidence, mortality, and DALYs of urinary tract infections from 1990 to 2021 at the global level **22**

**Table S3**. Incidence, mortality, and DALYs of urinary tract infections by age group in 2021, and their AAPCs from 1990 to 2021 **23**

**Table S4**. The age-standardized rate of incidence, mortality, and DALYs of urinary tract infections from 1990 to 2021 at the regional level **38**

**Table S5**. The case number and age-standardized rate of incidence of urinary tract infections in 1990 and 2021, and the temporal trends between 1990 and 2021 at the national level **68**

**Table S6**. The case number and age-standardized rate of mortality of urinary tract infections in 1990 and 2021, and the temporal trends between 1990 and 2021 at the national level **83**

**Table S7**. The case number and age-standardized rate of DALYs of urinary tract infections in 1990 and 2021, and the temporal trends between 1990 and 2021 at the national level **91**

**Figure S1**. Supplementary methods.


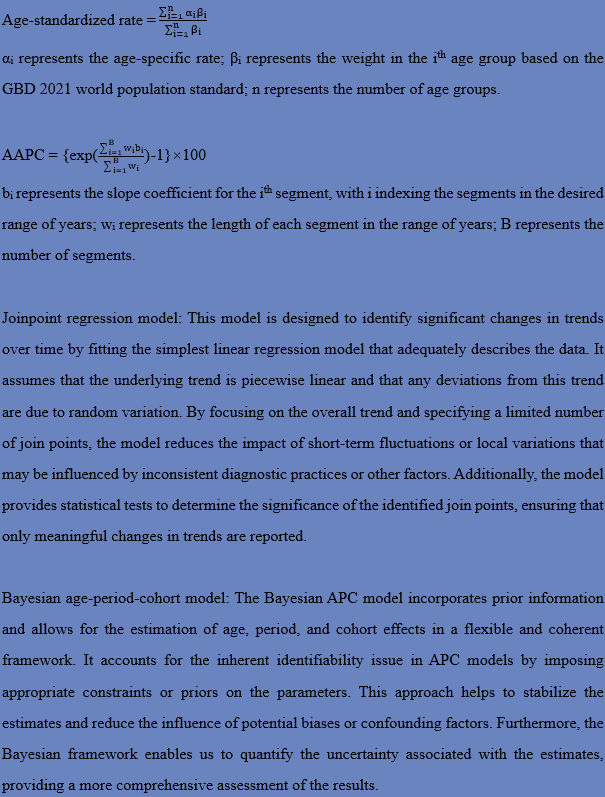


**Figure S2**. The Joinpoint regression analysis on the age-standardized incidence (A), mortality (B), and DALYs (C) rates of urinary tract infections among older women from 1990 to 2021 at the global level. DALYs, disability-adjusted life years; APC, annual percentage change.


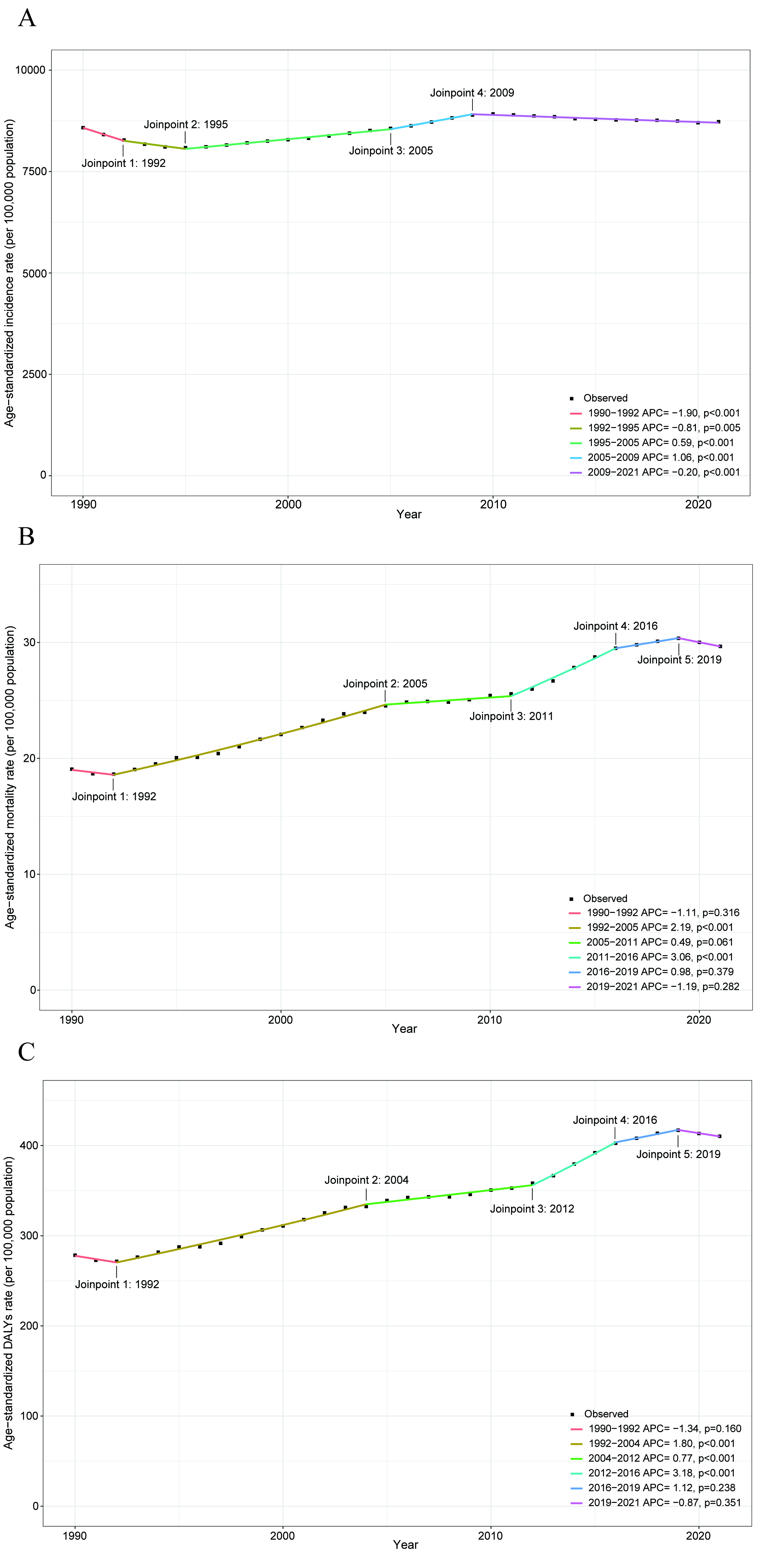


**Figure S3**. The Joinpoint regression analysis on the number of incident cases (A), deaths (B), and DALYs (C) of urinary tract infections among older women from 1990 to 2021 at the global level. DALYs, disability-adjusted life years; APC, annual percentage change.


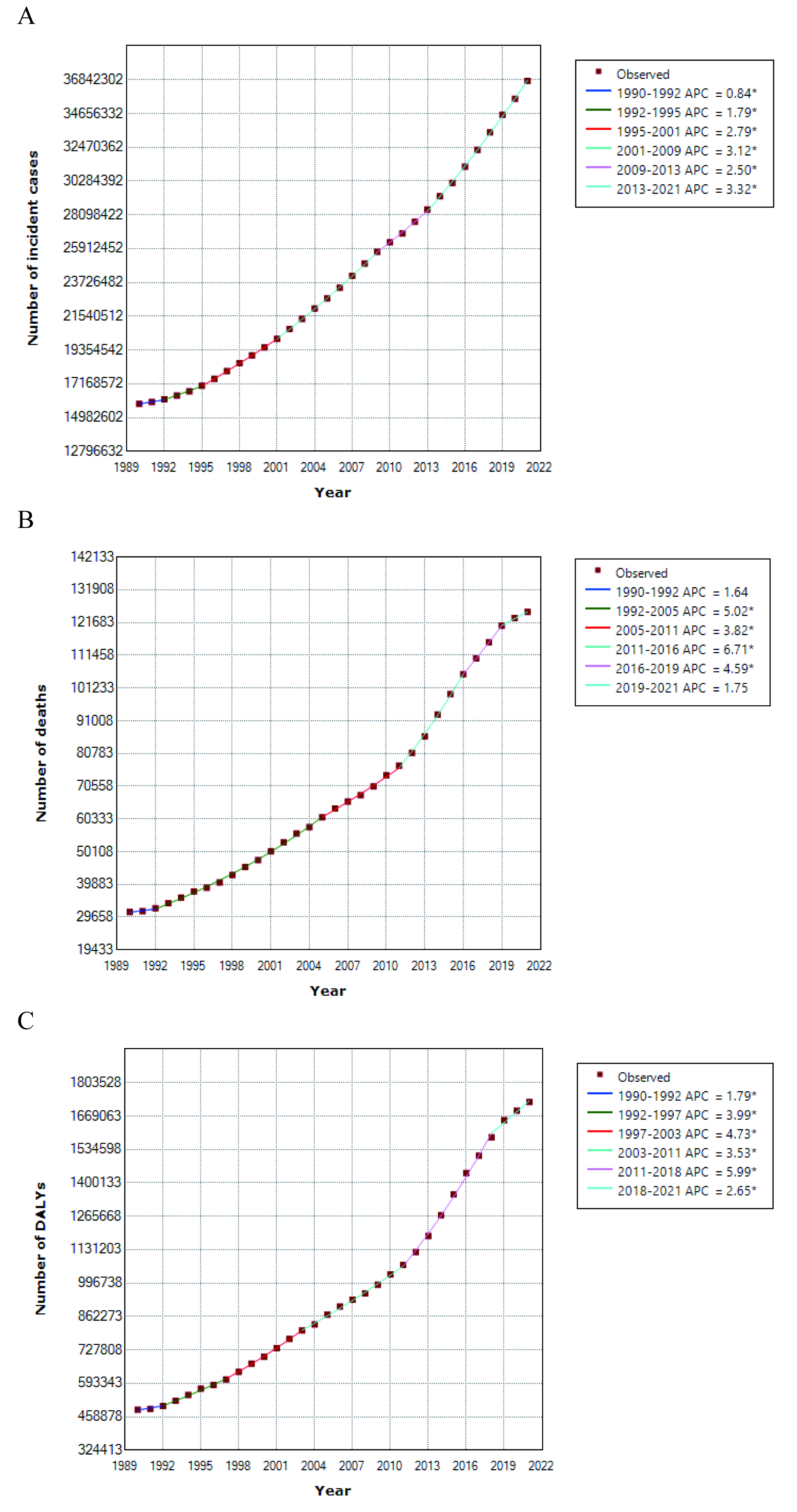


**Figure S4**. Temporal trends of the number and age-standardized incidence rate (A, B), age-standardized mortality rate (C, D), and age-standardized DALYs rate (E, F) for urinary tract infections among older women aged ≥ 65 years from 1990 to 2040. The solid line represents the observed age-standardized rate, and the dashed line represents the age-standardized rate predicted by the Nordpred model. UTIs, urinary tract infections; DALYs, disability-adjusted life-years.


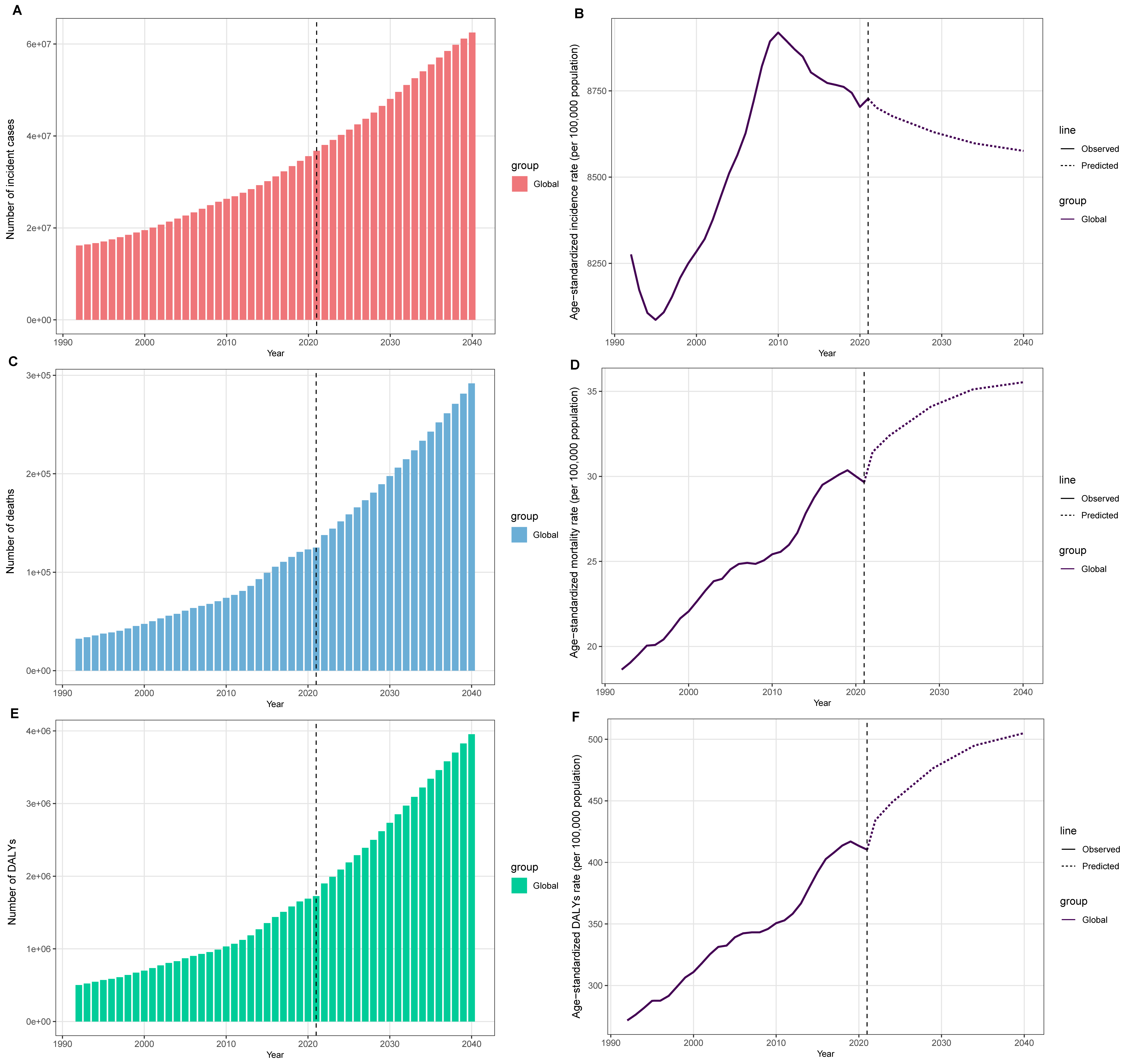


.

**Figure S5**. The line charts of the incidence (A), mortality (B), and DALYs (C) of urinary tract infections among older women from 1990 to 2021 at the global level in subgroup of age. DALYs, disability-adjusted life years.


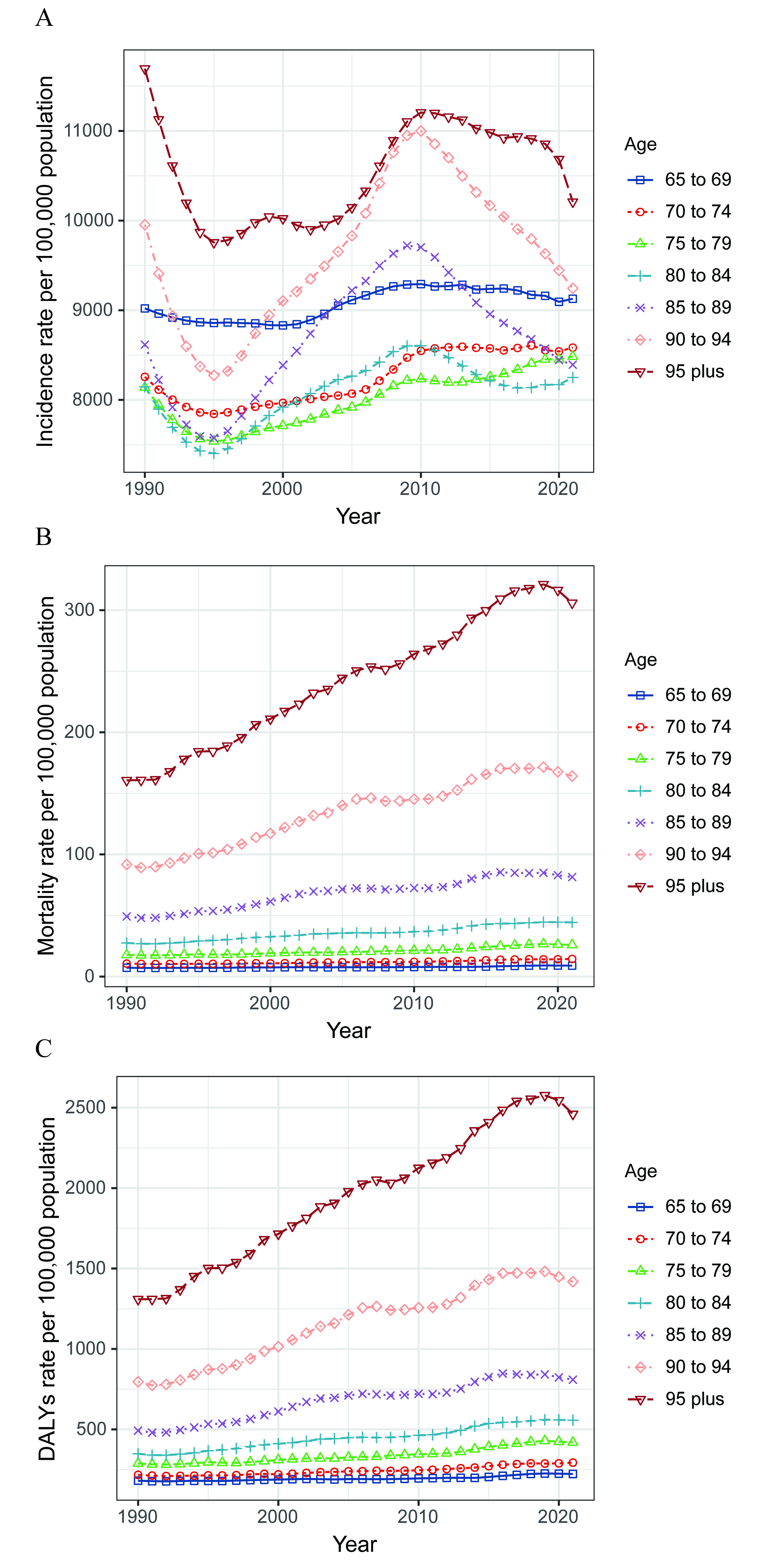


**Figure S6**. The line charts of the age-standardized incidence (A), mortality (B), and DALYs (C) rates of urinary tract infections among older women from 1990 to 2021 at the global level in subgroup of SDI. DALYs, disability-adjusted life years; SDI, sociodemographic index.


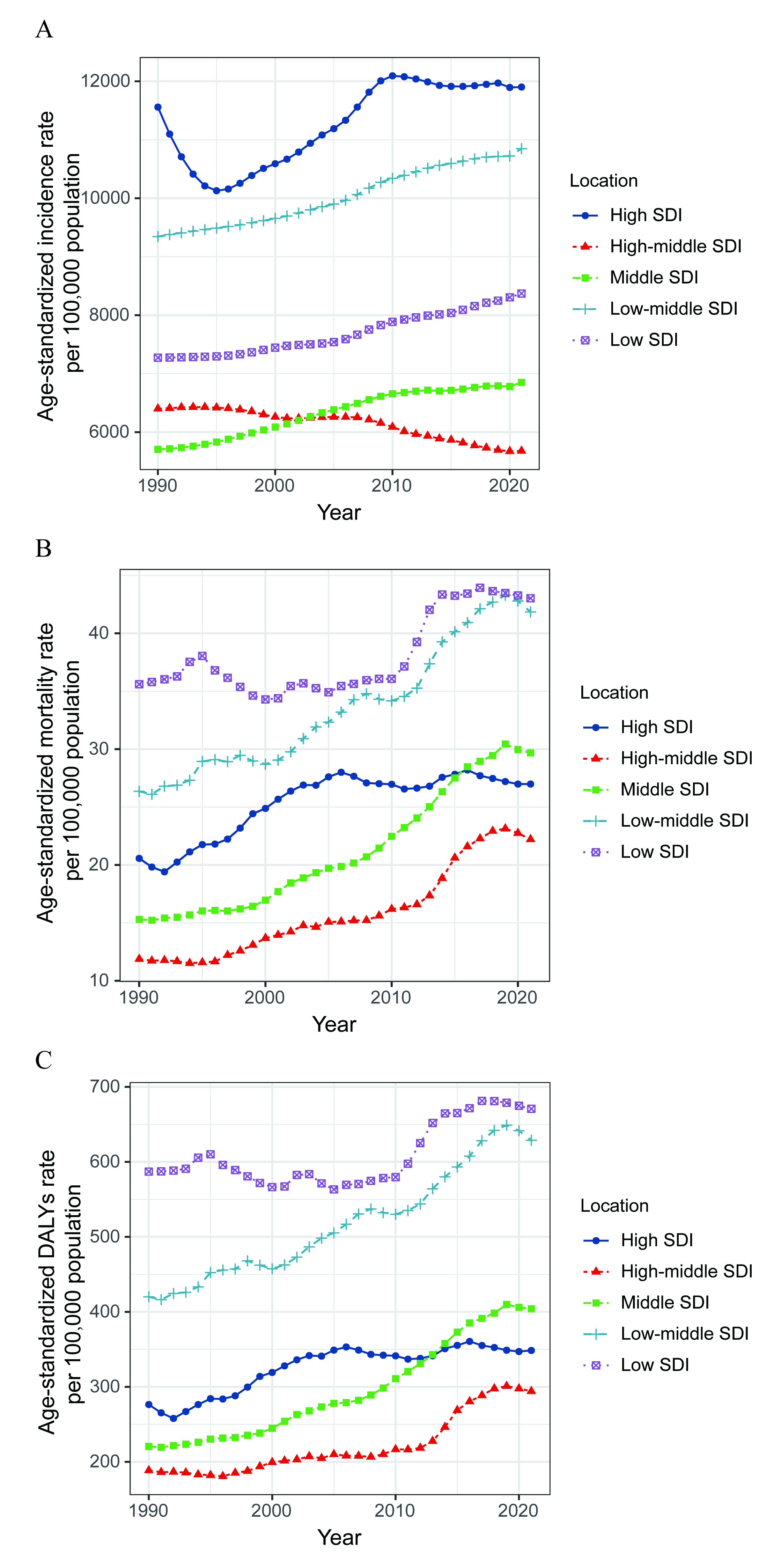


**Figure S7**. Global map of the age-standardized rate of global incidence (A), mortality (B), and DALYs (C) of urinary tract infections among older women in 1990. DALYs, disability-adjusted life years.


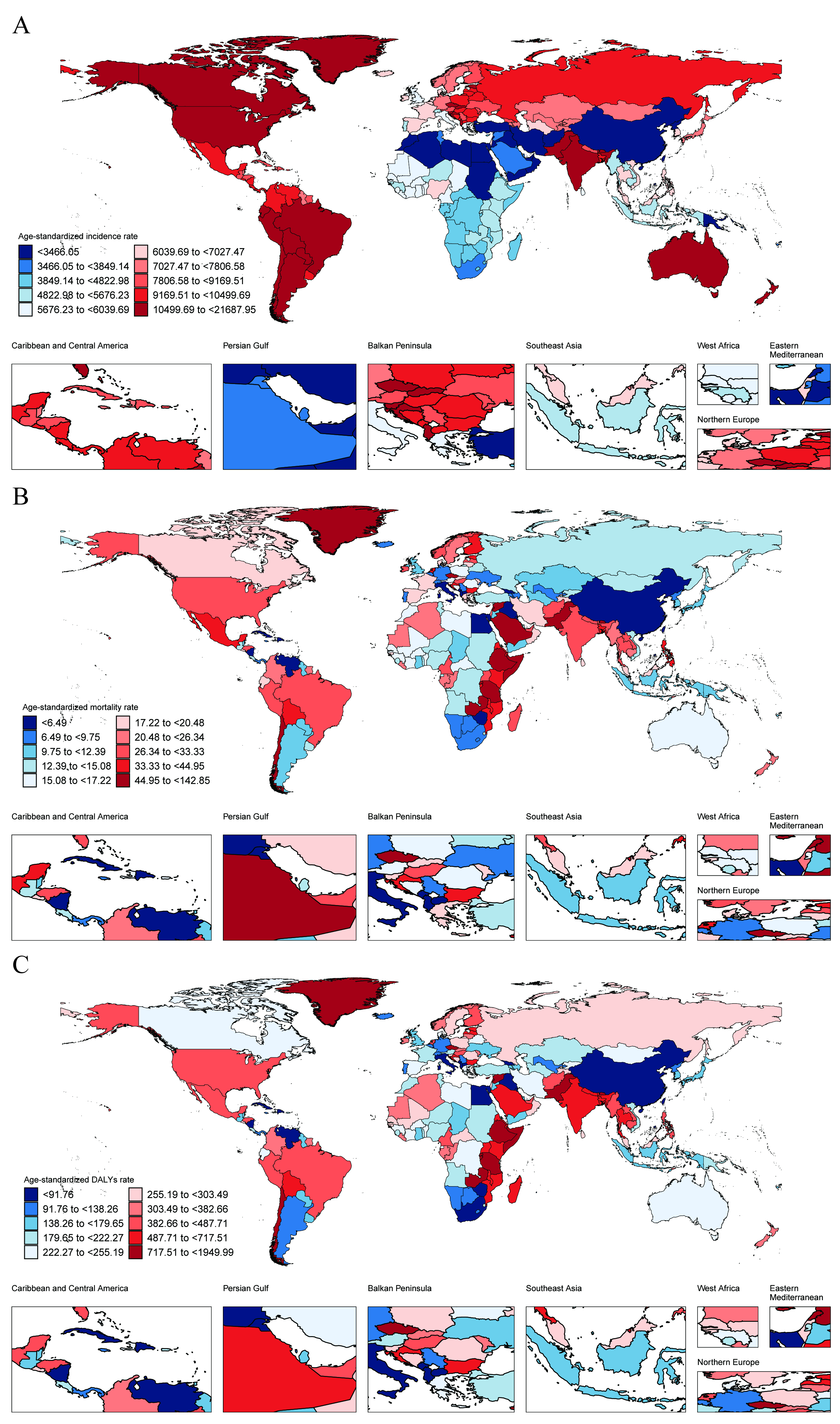


**Figure S8**. Global map of the age-standardized rate of global incidence (A), mortality (B), and DALYs (C) of urinary tract infections among older women in 2021. DALYs, disability-adjusted life years.


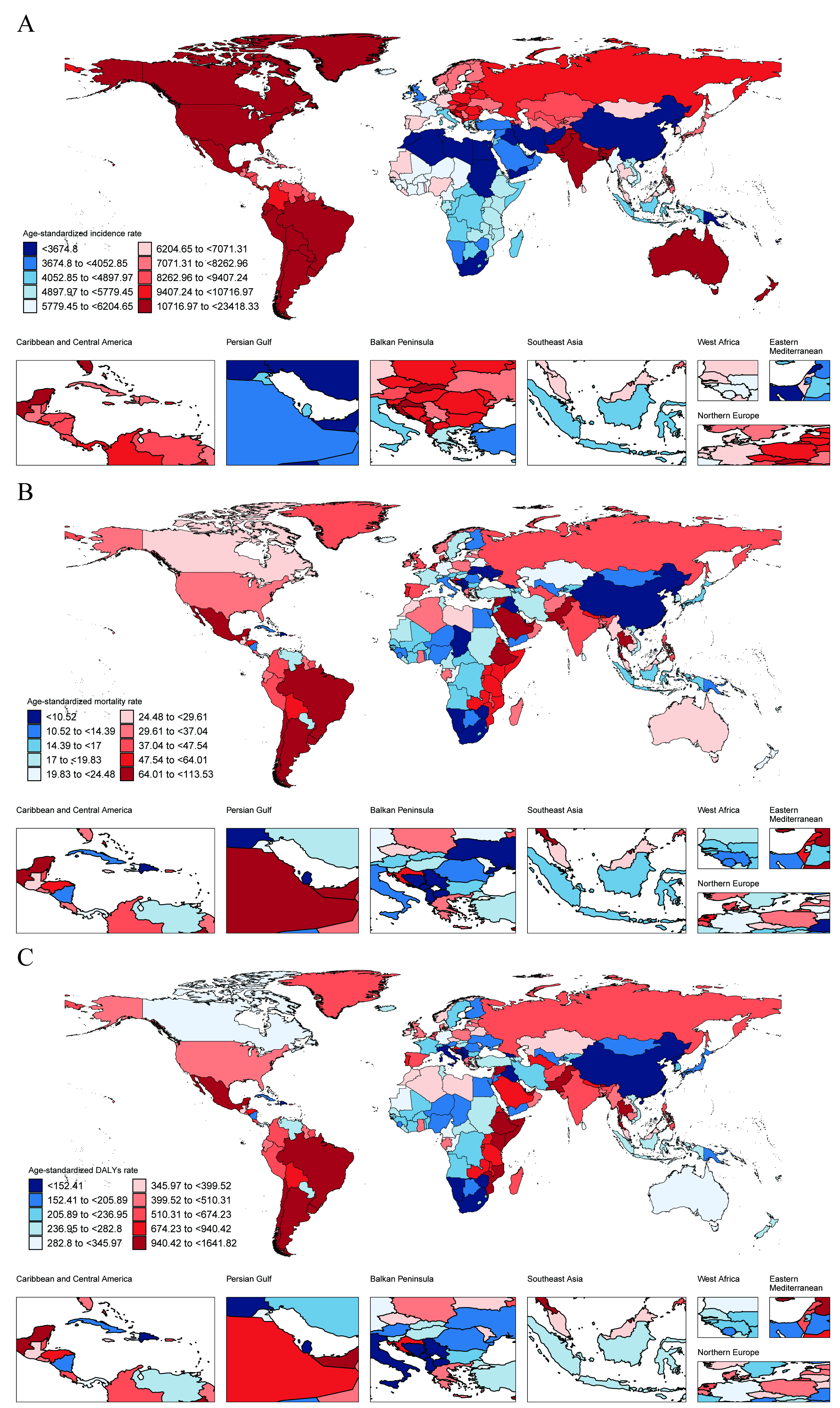


**Table S1. Geographical hierarchy and SDI quintiles for countries estimated in GBD 2021.**

| Country name | SDI Quintile | Region name | Super-region name |
| --- | --- | --- | --- |
| Albania | Middle SDI | Central Europe | Central Europe, Eastern Europe, and Central Asia |
| Bosnia and Herzegovina | High-middle SDI | Central Europe | Central Europe, Eastern Europe, and Central Asia |
| Bulgaria | High-middle SDI | Central Europe | Central Europe, Eastern Europe, and Central Asia |
| Croatia | High-middle SDI | Central Europe | Central Europe, Eastern Europe, and Central Asia |
| Czechia | High SDI | Central Europe | Central Europe, Eastern Europe, and Central Asia |
| Hungary | High-middle SDI | Central Europe | Central Europe, Eastern Europe, and Central Asia |
| Montenegro | High-middle SDI | Central Europe | Central Europe, Eastern Europe, and Central Asia |
| North Macedonia | High-middle SDI | Central Europe | Central Europe, Eastern Europe, and Central Asia |
| Poland | High SDI | Central Europe | Central Europe, Eastern Europe, and Central Asia |
| Romania | High-middle SDI | Central Europe | Central Europe, Eastern Europe, and Central Asia |
| Serbia | High-middle SDI | Central Europe | Central Europe, Eastern Europe, and Central Asia |
| Slovakia | High SDI | Central Europe | Central Europe, Eastern Europe, and Central Asia |
| Slovenia | High SDI | Central Europe | Central Europe, Eastern Europe, and Central Asia |
| Belarus | High-middle SDI | Eastern Europe | Central Europe, Eastern Europe, and Central Asia |
| Estonia | High SDI | Eastern Europe | Central Europe, Eastern Europe, and Central Asia |
| Latvia | High SDI | Eastern Europe | Central Europe, Eastern Europe, and Central Asia |
| Lithuania | High SDI | Eastern Europe | Central Europe, Eastern Europe, and Central Asia |
| Republic of Moldova | High-middle SDI | Eastern Europe | Central Europe, Eastern Europe, and Central Asia |
| Russian Federation | High-middle SDI | Eastern Europe | Central Europe, Eastern Europe, and Central Asia |
| Ukraine | High-middle SDI | Eastern Europe | Central Europe, Eastern Europe, and Central Asia |
| Armenia | Middle SDI | Central Asia | Central Europe, Eastern Europe, and Central Asia |
| Azerbaijan | Middle SDI | Central Asia | Central Europe, Eastern Europe, and Central Asia |
| Georgia | High-middle SDI | Central Asia | Central Europe, Eastern Europe, and Central Asia |
| Kazakhstan | High-middle SDI | Central Asia | Central Europe, Eastern Europe, and Central Asia |
| Kyrgyzstan | Low-middle SDI | Central Asia | Central Europe, Eastern Europe, and Central Asia |
| Mongolia | Low-middle SDI | Central Asia | Central Europe, Eastern Europe, and Central Asia |
| Tajikistan | Low-middle SDI | Central Asia | Central Europe, Eastern Europe, and Central Asia |
| Turkmenistan | Middle SDI | Central Asia | Central Europe, Eastern Europe, and Central Asia |
| Uzbekistan | Middle SDI | Central Asia | Central Europe, Eastern Europe, and Central Asia |
| Australia | High SDI | Australasia | High-income |
| New Zealand | High SDI | Australasia | High-income |
| Brunei Darussalam | High-middle SDI | High-income Asia Pacific | High-income |
| Japan | High SDI | High-income Asia Pacific | High-income |
| Republic of Korea | High SDI | High-income Asia Pacific | High-income |
| Singapore | High SDI | High-income Asia Pacific | High-income |
| Canada | High SDI | High-income North America | High-income |
| Greenland | High SDI | High-income North America | High-income |
| United States of America | High SDI | High-income North America | High-income |
| Argentina | High-middle SDI | Southern Latin America | High-income |
| Chile | High-middle SDI | Southern Latin America | High-income |
| Uruguay | High-middle SDI | Southern Latin America | High-income |
| Andorra | High SDI | Western Europe | High-income |
| Austria | High SDI | Western Europe | High-income |
| Belgium | High SDI | Western Europe | High-income |
| Cyprus | High SDI | Western Europe | High-income |
| Denmark | High SDI | Western Europe | High-income |
| Finland | High SDI | Western Europe | High-income |
| France | High SDI | Western Europe | High-income |
| Germany | High SDI | Western Europe | High-income |
| Greece | High-middle SDI | Western Europe | High-income |
| Iceland | High SDI | Western Europe | High-income |
| Ireland | High SDI | Western Europe | High-income |
| Israel | High-middle SDI | Western Europe | High-income |
| Italy | High-middle SDI | Western Europe | High-income |
| Luxembourg | High SDI | Western Europe | High-income |
| Malta | High-middle SDI | Western Europe | High-income |
| Monaco | High SDI | Western Europe | High-income |
| Netherlands | High SDI | Western Europe | High-income |
| Norway | High SDI | Western Europe | High-income |
| Portugal | High-middle SDI | Western Europe | High-income |
| San Marino | High SDI | Western Europe | High-income |
| Spain | High-middle SDI | Western Europe | High-income |
| Sweden | High SDI | Western Europe | High-income |
| Switzerland | High SDI | Western Europe | High-income |
| United Kingdom | High SDI | Western Europe | High-income |
| Bolivia (Plurinational State of) | Low-middle SDI | Andean Latin America | Latin America and Caribbean |
| Ecuador | Middle SDI | Andean Latin America | Latin America and Caribbean |
| Peru | Middle SDI | Andean Latin America | Latin America and Caribbean |
| Colombia | Middle SDI | Central Latin America | Latin America and Caribbean |
| Costa Rica | Middle SDI | Central Latin America | Latin America and Caribbean |
| El Salvador | Low-middle SDI | Central Latin America | Latin America and Caribbean |
| Guatemala | Low-middle SDI | Central Latin America | Latin America and Caribbean |
| Honduras | Low-middle SDI | Central Latin America | Latin America and Caribbean |
| Mexico | Middle SDI | Central Latin America | Latin America and Caribbean |
| Nicaragua | Low-middle SDI | Central Latin America | Latin America and Caribbean |
| Panama | Middle SDI | Central Latin America | Latin America and Caribbean |
| Venezuela (Bolivarian Republic of) | Low-middle SDI | Central Latin America | Latin America and Caribbean |
| Brazil | Middle SDI | Tropical Latin America | Latin America and Caribbean |
| Paraguay | Middle SDI | Tropical Latin America | Latin America and Caribbean |
| Antigua and Barbuda | High-middle SDI | Caribbean | Latin America and Caribbean |
| Bahamas | High-middle SDI | Caribbean | Latin America and Caribbean |
| Barbados | High-middle SDI | Caribbean | Latin America and Caribbean |
| Belize | Low-middle SDI | Caribbean | Latin America and Caribbean |
| Bermuda | High SDI | Caribbean | Latin America and Caribbean |
| Cuba | Middle SDI | Caribbean | Latin America and Caribbean |
| Dominica | High-middle SDI | Caribbean | Latin America and Caribbean |
| Dominican Republic | Middle SDI | Caribbean | Latin America and Caribbean |
| Grenada | Middle SDI | Caribbean | Latin America and Caribbean |
| Guyana | Middle SDI | Caribbean | Latin America and Caribbean |
| Haiti | Low SDI | Caribbean | Latin America and Caribbean |
| Jamaica | Middle SDI | Caribbean | Latin America and Caribbean |
| Puerto Rico | High SDI | Caribbean | Latin America and Caribbean |
| Saint Kitts and Nevis | High-middle SDI | Caribbean | Latin America and Caribbean |
| Saint Lucia | Middle SDI | Caribbean | Latin America and Caribbean |
| Saint Vincent and the Grenadines | Middle SDI | Caribbean | Latin America and Caribbean |
| Suriname | Middle SDI | Caribbean | Latin America and Caribbean |
| Trinidad and Tobago | High-middle SDI | Caribbean | Latin America and Caribbean |
| United States Virgin Islands | High SDI | Caribbean | Latin America and Caribbean |
| Afghanistan | Low SDI | North Africa and Middle East | North Africa and Middle East |
| Algeria | Middle SDI | North Africa and Middle East | North Africa and Middle East |
| Bahrain | High-middle SDI | North Africa and Middle East | North Africa and Middle East |
| Egypt | Low-middle SDI | North Africa and Middle East | North Africa and Middle East |
| Iran (Islamic Republic of) | Middle SDI | North Africa and Middle East | North Africa and Middle East |
| Iraq | Middle SDI | North Africa and Middle East | North Africa and Middle East |
| Jordan | High-middle SDI | North Africa and Middle East | North Africa and Middle East |
| Kuwait | High SDI | North Africa and Middle East | North Africa and Middle East |
| Lebanon | High-middle SDI | North Africa and Middle East | North Africa and Middle East |
| Libya | High-middle SDI | North Africa and Middle East | North Africa and Middle East |
| Morocco | Low-middle SDI | North Africa and Middle East | North Africa and Middle East |
| Oman | High-middle SDI | North Africa and Middle East | North Africa and Middle East |
| Palestine | Middle SDI | North Africa and Middle East | North Africa and Middle East |
| Qatar | High SDI | North Africa and Middle East | North Africa and Middle East |
| Saudi Arabia | High SDI | North Africa and Middle East | North Africa and Middle East |
| Sudan | Low-middle SDI | North Africa and Middle East | North Africa and Middle East |
| Syrian Arab Republic | Middle SDI | North Africa and Middle East | North Africa and Middle East |
| Tunisia | Middle SDI | North Africa and Middle East | North Africa and Middle East |
| Turkey | High-middle SDI | North Africa and Middle East | North Africa and Middle East |
| United Arab Emirates | High SDI | North Africa and Middle East | North Africa and Middle East |
| Yemen | Low SDI | North Africa and Middle East | North Africa and Middle East |
| Bangladesh | Low-middle SDI | South Asia | South Asia |
| Bhutan | Low-middle SDI | South Asia | South Asia |
| India | Low-middle SDI | South Asia | South Asia |
| Nepal | Low SDI | South Asia | South Asia |
| Pakistan | Low-middle SDI | South Asia | South Asia |
| Cambodia | Low-middle SDI | Southeast Asia | Southeast Asia, East Asia, and Oceania |
| Indonesia | Middle SDI | Southeast Asia | Southeast Asia, East Asia, and Oceania |
| Lao People’s Democratic Republic | Low-middle SDI | Southeast Asia | Southeast Asia, East Asia, and Oceania |
| Malaysia | High-middle SDI | Southeast Asia | Southeast Asia, East Asia, and Oceania |
| Maldives | Middle SDI | Southeast Asia | Southeast Asia, East Asia, and Oceania |
| Mauritius | High-middle SDI | Southeast Asia | Southeast Asia, East Asia, and Oceania |
| Myanmar | Low-middle SDI | Southeast Asia | Southeast Asia, East Asia, and Oceania |
| Philippines | Middle SDI | Southeast Asia | Southeast Asia, East Asia, and Oceania |
| Seychelles | High-middle SDI | Southeast Asia | Southeast Asia, East Asia, and Oceania |
| Sri Lanka | Middle SDI | Southeast Asia | Southeast Asia, East Asia, and Oceania |
| Thailand | Middle SDI | Southeast Asia | Southeast Asia, East Asia, and Oceania |
| Timor-Leste | Low SDI | Southeast Asia | Southeast Asia, East Asia, and Oceania |
| Viet Nam | Middle SDI | Southeast Asia | Southeast Asia, East Asia, and Oceania |
| China | High-middle SDI | East Asia | Southeast Asia, East Asia, and Oceania |
| Democratic People’s Republic of Korea | Low-middle SDI | East Asia | Southeast Asia, East Asia, and Oceania |
| Taiwan (Province of China) | High SDI | East Asia | Southeast Asia, East Asia, and Oceania |
| American Samoa | High-middle SDI | Oceania | Southeast Asia, East Asia, and Oceania |
| Cook Islands | High-middle SDI | Oceania | Southeast Asia, East Asia, and Oceania |
| Fiji | Middle SDI | Oceania | Southeast Asia, East Asia, and Oceania |
| Guam | High-middle SDI | Oceania | Southeast Asia, East Asia, and Oceania |
| Kiribati | Low-middle SDI | Oceania | Southeast Asia, East Asia, and Oceania |
| Marshall Islands | Low-middle SDI | Oceania | Southeast Asia, East Asia, and Oceania |
| Micronesia (Federated States of) | Low-middle SDI | Oceania | Southeast Asia, East Asia, and Oceania |
| Nauru | Middle SDI | Oceania | Southeast Asia, East Asia, and Oceania |
| Niue | High-middle SDI | Oceania | Southeast Asia, East Asia, and Oceania |
| Northern Mariana Islands | High-middle SDI | Oceania | Southeast Asia, East Asia, and Oceania |
| Palau | High-middle SDI | Oceania | Southeast Asia, East Asia, and Oceania |
| Papua New Guinea | Low SDI | Oceania | Southeast Asia, East Asia, and Oceania |
| Samoa | Low-middle SDI | Oceania | Southeast Asia, East Asia, and Oceania |
| Solomon Islands | Low SDI | Oceania | Southeast Asia, East Asia, and Oceania |
| Tokelau | Middle SDI | Oceania | Southeast Asia, East Asia, and Oceania |
| Tonga | Middle SDI | Oceania | Southeast Asia, East Asia, and Oceania |
| Tuvalu | Low-middle SDI | Oceania | Southeast Asia, East Asia, and Oceania |
| Vanuatu | Low-middle SDI | Oceania | Southeast Asia, East Asia, and Oceania |
| Angola | Low SDI | Central Sub-Saharan Africa | Sub-Saharan Africa |
| Central African Republic | Low SDI | Central Sub-Saharan Africa | Sub-Saharan Africa |
| Congo | Low SDI | Central Sub-Saharan Africa | Sub-Saharan Africa |
| Democratic Republic of the Congo | Low SDI | Central Sub-Saharan Africa | Sub-Saharan Africa |
| Equatorial Guinea | Middle SDI | Central Sub-Saharan Africa | Sub-Saharan Africa |
| Gabon | Middle SDI | Central Sub-Saharan Africa | Sub-Saharan Africa |
| Burundi | Low SDI | Eastern sub-Saharan Africa | Sub-Saharan Africa |
| Comoros | Low-middle SDI | Eastern sub-Saharan Africa | Sub-Saharan Africa |
| Djibouti | Low-middle SDI | Eastern sub-Saharan Africa | Sub-Saharan Africa |
| Eritrea | Low SDI | Eastern sub-Saharan Africa | Sub-Saharan Africa |
| Ethiopia | Low SDI | Eastern sub-Saharan Africa | Sub-Saharan Africa |
| Kenya | Low-middle SDI | Eastern sub-Saharan Africa | Sub-Saharan Africa |
| Madagascar | Low SDI | Eastern sub-Saharan Africa | Sub-Saharan Africa |
| Malawi | Low SDI | Eastern sub-Saharan Africa | Sub-Saharan Africa |
| Mozambique | Low SDI | Eastern sub-Saharan Africa | Sub-Saharan Africa |
| Rwanda | Low SDI | Eastern sub-Saharan Africa | Sub-Saharan Africa |
| Somalia | Low SDI | Eastern sub-Saharan Africa | Sub-Saharan Africa |
| South Sudan | Low SDI | Eastern sub-Saharan Africa | Sub-Saharan Africa |
| Uganda | Low SDI | Eastern sub-Saharan Africa | Sub-Saharan Africa |
| United Republic of Tanzania | Low SDI | Eastern sub-Saharan Africa | Sub-Saharan Africa |
| Zambia | Low-middle SDI | Eastern sub-Saharan Africa | Sub-Saharan Africa |
| Botswana | Middle SDI | Southern Sub-Saharan Africa | Sub-Saharan Africa |
| Eswatini | Low-middle SDI | Southern Sub-Saharan Africa | Sub-Saharan Africa |
| Lesotho | Low-middle SDI | Southern Sub-Saharan Africa | Sub-Saharan Africa |
| Namibia | Low-middle SDI | Southern Sub-Saharan Africa | Sub-Saharan Africa |
| South Africa | Middle SDI | Southern Sub-Saharan Africa | Sub-Saharan Africa |
| Zimbabwe | Low-middle SDI | Southern Sub-Saharan Africa | Sub-Saharan Africa |
| Benin | Low SDI | Western Sub-Saharan Africa | Sub-Saharan Africa |
| Burkina Faso | Low SDI | Western Sub-Saharan Africa | Sub-Saharan Africa |
| Cabo Verde | Low-middle SDI | Western Sub-Saharan Africa | Sub-Saharan Africa |
| Cameroon | Low-middle SDI | Western Sub-Saharan Africa | Sub-Saharan Africa |
| Chad | Low SDI | Western Sub-Saharan Africa | Sub-Saharan Africa |
| Côte d’Ivoire | Low SDI | Western Sub-Saharan Africa | Sub-Saharan Africa |
| Gambia | Low SDI | Western Sub-Saharan Africa | Sub-Saharan Africa |
| Ghana | Low-middle SDI | Western Sub-Saharan Africa | Sub-Saharan Africa |
| Guinea | Low SDI | Western Sub-Saharan Africa | Sub-Saharan Africa |
| Guinea-Bissau | Low SDI | Western Sub-Saharan Africa | Sub-Saharan Africa |
| Liberia | Low SDI | Western Sub-Saharan Africa | Sub-Saharan Africa |
| Mali | Low SDI | Western Sub-Saharan Africa | Sub-Saharan Africa |
| Mauritania | Low-middle SDI | Western Sub-Saharan Africa | Sub-Saharan Africa |
| Niger | Low SDI | Western Sub-Saharan Africa | Sub-Saharan Africa |
| Nigeria | Low-middle SDI | Western Sub-Saharan Africa | Sub-Saharan Africa |
| Sao Tome and Principe | Low-middle SDI | Western Sub-Saharan Africa | Sub-Saharan Africa |
| Senegal | Low SDI | Western Sub-Saharan Africa | Sub-Saharan Africa |
| Sierra Leone | Low SDI | Western Sub-Saharan Africa | Sub-Saharan Africa |
| Togo | Low SDI | Western Sub-Saharan Africa | Sub-Saharan Africa |

Abbreviations: GBD, Global Burden of Diseases, Injuries, and Risk Factors Study; SDI, sociodemographic index.

**Table S2. The age-standardized rate of incidence, mortality, and DALYs of urinary tract infections from 1990 to 2021 at the global level.**

| Year | Rate (95% UI) per 100 000 population | | |
| --- | --- | --- | --- |
|  | Incidence | Mortality | DALYs |
| 1990 | 8580.94 (6985.22-10446.58) | 19.06 (16.18-22.17) | 278.42 (239.85-325.44) |
| 1991 | 8411.99 (6855.52-10215.64) | 18.67 (15.82-21.64) | 272.85 (234.3-317.45) |
| 1992 | 8275.93 (6762.56-10023.13) | 18.64 (15.82-21.57) | 271.68 (233.97-315.65) |
| 1993 | 8173.01 (6689.58-9875.01) | 19.04 (16.16-21.99) | 276.26 (237.71-320.5) |
| 1994 | 8106.44 (6648.89-9779.57) | 19.52 (16.53-22.52) | 281.72 (241.92-326.98) |
| 1995 | 8086.34 (6643.24-9745.73) | 20.05 (16.97-23.08) | 287.6 (246.95-332.95) |
| 1996 | 8108.18 (6663.41-9770.29) | 20.09 (16.99-23.13) | 287.71 (246.99-333.16) |
| 1997 | 8152.63 (6714.88-9820.73) | 20.41 (17.22-23.4) | 291.57 (249.22-336.6) |
| 1998 | 8207.72 (6772.41-9878.01) | 21 (17.72-23.93) | 298.97 (255.45-341.91) |
| 1999 | 8249.97 (6809.84-9927.01) | 21.65 (18.25-24.67) | 306.51 (261.82-350.94) |
| 2000 | 8284.35 (6842.9-9965.26) | 22.06 (18.55-25.12) | 310.92 (265.17-355.94) |
| 2001 | 8320.4 (6882.09-9990.17) | 22.66 (18.99-25.64) | 317.98 (270.4-362.12) |
| 2002 | 8376.93 (6929.34-10048.77) | 23.28 (19.52-26.3) | 325.42 (276.93-369) |
| 2003 | 8444.96 (6980.35-10123.53) | 23.84 (19.95-26.89) | 331.37 (281.64-375.19) |
| 2004 | 8511.24 (7033.44-10193.87) | 23.97 (19.99-27.24) | 332.43 (281.85-379.69) |
| 2005 | 8563.62 (7073.4-10238.03) | 24.53 (20.46-27.8) | 339.16 (287.83-386.01) |
| 2006 | 8626.81 (7143.06-10290.07) | 24.85 (20.6-28.09) | 342.39 (289.41-388.68) |
| 2007 | 8720.84 (7238.42-10379.75) | 24.91 (20.64-28.18) | 343.15 (289.61-389.63) |
| 2008 | 8820.92 (7334.97-10472.27) | 24.86 (20.55-28.12) | 343.19 (289.7-389.38) |
| 2009 | 8893.47 (7404.74-10542.99) | 25.06 (20.68-28.4) | 345.91 (291.61-393.37) |
| 2010 | 8918.85 (7424.79-10578.08) | 25.42 (20.97-28.88) | 350.66 (295.64-399.39) |
| 2011 | 8894.93 (7420.98-10539.72) | 25.56 (21.07-28.95) | 352.88 (297-400.18) |
| 2012 | 8870.35 (7416.61-10499.96) | 25.97 (21.33-29.35) | 358.27 (300.68-405.49) |
| 2013 | 8849.15 (7417.04-10456.34) | 26.68 (21.97-30.06) | 366.68 (308.18-413.65) |
| 2014 | 8803.45 (7395.88-10396.1) | 27.83 (22.97-31.23) | 379.55 (319.73-426.45) |
| 2015 | 8787.56 (7397.02-10379.35) | 28.75 (23.67-32.15) | 392.08 (330.14-438.06) |
| 2016 | 8772.66 (7381.14-10390.02) | 29.5 (24.27-33.02) | 402.72 (338.5-449.83) |
| 2017 | 8767.41 (7365.73-10410.86) | 29.8 (24.56-33.27) | 408.06 (343.42-454.76) |
| 2018 | 8761.43 (7326.99-10430.83) | 30.11 (24.83-33.71) | 413.69 (347.93-462.83) |
| 2019 | 8744.03 (7286.8-10416.78) | 30.36 (24.95-34.05) | 416.99 (349.4-467.15) |
| 2020 | 8703.33 (7243.97-10446.29) | 30.01 (24.46-33.67) | 413.43 (344.09-462.77) |
| 2021 | 8727.44 (7190.01-10547.59) | 29.66 (24.18-33.28) | 410.33 (341.96-459.02) |

Abbreviations: DALYs, disability-adjusted life-years; UI, uncertainty interval.

**Table S3. Incidence, mortality, and DALYs of urinary tract infections by age group in 2021, and their AAPCs from 1990 to 2021.**

|  | Incidence | |  | Mortality | |  | DALYs | |
| --- | --- | --- | --- | --- | --- | --- | --- | --- |
|  | Rate per 100 000 population, 2021 | AAPC, 1990-2021 |  | Rate per 100 000 population, 2021 | AAPC, 1990-2021 |  | Rate per 100 000 population, 2021 | AAPC, 1990-2021 |
| **Global** | | | | | | | | |
| 65-69 years | 9128.23 (7453.31-11005.63) | 0.03 (-0.01 to 0.07) |  | 8.93 (7.64-10.04) | 0.73 (0.51 to 0.95)^*^ |  | 222.61 (190.87-250.31) | 0.71 (0.5 to 0.92)^*^ |
| 70-74 years | 8583.34 (7174.16-10300.63) | 0.13 (0.09 to 0.17)^*^ |  | 14.42 (12.28-16.06) | 0.98 (0.69 to 1.27)^*^ |  | 293.45 (251.21-326.33) | 0.96 (0.67 to 1.26)^*^ |
| 75-79 years | 8484.25 (6985.93-10247.73) | 0.14 (0.06 to 0.22)^*^ |  | 25.97 (22.69-28.78) | 1.25 (1 to 1.5)^*^ |  | 419.04 (367.25-464.35) | 1.24 (1 to 1.48)^*^ |
| 80-84 years | 8250.52 (6926.65-10137.95) | 0.03 (-0.06 to 0.12) |  | 44.36 (37.5-49.12) | 1.6 (1.39 to 1.8)^*^ |  | 556.85 (472.82-616.62) | 1.57 (1.38 to 1.76)^*^ |
| 85-89 years | 8393.02 (6833.62-10050.33) | -0.1 (-0.26 to 0.05) |  | 81.46 (65.3-91.17) | 1.69 (1.37 to 2.01)^*^ |  | 808.82 (649.24-904.63) | 1.66 (1.34 to 1.98)^*^ |
| 90-94 years | 9245.08 (7340.47-11328.68) | -0.21 (-0.43 to 0.01) |  | 164.13 (123.28-186.55) | 1.96 (1.63 to 2.29)^*^ |  | 1418.06 (1066.41-1610.74) | 1.94 (1.62 to 2.27)^*^ |
| 95+ years | 10209.57 (7741.85-13460.05) | -0.4 (-0.57 to -0.23)^*^ |  | 305.89 (213.2-356.28) | 2.22 (1.79 to 2.66)^*^ |  | 2460.44 (1718.93-2862.74) | 2.18 (1.74 to 2.61)^*^ |
| **High SDI** | | | | | | | | |
| 65-69 years | 10351.13 (8200.87-12656.15) | -0.05 (-0.14 to 0.04) |  | 5.14 (4.75-5.55) | 0.46 (0.23 to 0.69)^*^ |  | 131.14 (120.86-141.39) | 0.44 (0.25 to 0.62)^*^ |
| 70-74 years | 11159.69 (9154.27-13526.27) | 0.02 (-0.07 to 0.11) |  | 10.01 (9.04-11.09) | 0.52 (0.11 to 0.94)^*^ |  | 205.76 (186-227.71) | 0.49 (0.08 to 0.9)^*^ |
| 75-79 years | 12976.81 (10401.13-15926.12) | 0.33 (0.24 to 0.42)^*^ |  | 21.79 (19.04-23.6) | 0.56 (-0.14 to 1.27) |  | 353.24 (309.13-382.01) | 0.57 (-0.15 to 1.29) |
| 80-84 years | 13584.96 (11392.52-16518.34) | 0.28 (0.17 to 0.39)^*^ |  | 41.9 (33.47-47.87) | 0.72 (0.45 to 0.98)^*^ |  | 527.35 (422.38-599.08) | 0.7 (0.42 to 0.97)^*^ |
| 85-89 years | 14528.94 (11505.6-17579.07) | 0.16 (0.02 to 0.3)^*^ |  | 84.54 (63.74-97.82) | 0.95 (0.61 to 1.3)^*^ |  | 839.21 (635.25-969.76) | 0.92 (0.52 to 1.33)^*^ |
| 90-94 years | 15458.12 (11938.24-19279.2) | 0.01 (-0.32 to 0.34) |  | 177.42 (130.21-204.82) | 1.32 (0.96 to 1.68)^*^ |  | 1535.01 (1128.68-1770.16) | 1.31 (0.95 to 1.66)^*^ |
| 95+ years | 15473.76 (11521.44-20696.18) | -0.43 (-0.64 to -0.21)^*^ |  | 329.33 (227.09-386.35) | 1.62 (1.37 to 1.86)^*^ |  | 2638.12 (1823.72-3089.94) | 1.57 (1.31 to 1.83)^*^ |
| **High-middle SDI** | | | | | | | | |
| 65-69 years | 6659.09 (5456.44-7896.9) | -0.64 (-0.74 to -0.55)^*^ |  | 5.42 (4.88-6) | -0.45 (-1 to 0.1) |  | 135.82 (123.16-149.88) | -0.48 (-0.94 to -0.03)^*^ |
| 70-74 years | 5954.42 (4994.92-7031.84) | -0.34 (-0.42 to -0.25)^*^ |  | 9.63 (8.56-10.83) | 0.6 (0.08 to 1.12)^*^ |  | 196.11 (175.14-220.28) | 0.64 (0.31 to 0.97)^*^ |
| 75-79 years | 5157.8 (4306.15-6155.04) | -0.46 (-0.57 to -0.36)^*^ |  | 16.9 (15.27-18.55) | 1.01 (0.52 to 1.5)^*^ |  | 271.33 (245.32-297.51) | 1.06 (0.71 to 1.4)^*^ |
| 80-84 years | 4797.87 (3959.37-5976.79) | 0.03 (-0.01 to 0.08) |  | 34.61 (29.84-37.75) | 2.53 (2.04 to 3.02)^*^ |  | 432.03 (372.34-470.63) | 2.47 (1.99 to 2.96)^*^ |
| 85-89 years | 3960.28 (3252.37-4776.02) | 0.37 (0.28 to 0.45)^*^ |  | 67.25 (54.44-75.39) | 3.39 (3.01 to 3.77)^*^ |  | 665.14 (539.34-745.04) | 3.35 (2.97 to 3.72)^*^ |
| 90-94 years | 3540.48 (2866.05-4314.17) | 0.88 (0.79 to 0.97)^*^ |  | 137.98 (106.04-156.56) | 3.74 (3.08 to 4.41)^*^ |  | 1190.06 (915.25-1350.12) | 3.73 (3.07 to 4.4)^*^ |
| 95+ years | 3433.65 (2424.43-4529.96) | 1.23 (1.11 to 1.35)^*^ |  | 248.36 (182.28-283.44) | 4.16 (3.59 to 4.73)^*^ |  | 2018.18 (1481.93-2301.52) | 4.13 (3.58 to 4.69)^*^ |
| **Middle SDI** | | | | | | | | |
| 65-69 years | 8047.13 (6573.75-9567.17) | 0.49 (0.41 to 0.57)^*^ |  | 8.36 (7.21-9.74) | 1.55 (1.31 to 1.78)^*^ |  | 208.4 (179.95-241.75) | 1.46 (1.21 to 1.7)^*^ |
| 70-74 years | 6980.17 (5765.48-8357.78) | 0.62 (0.56 to 0.68)^*^ |  | 13.7 (11.82-15.87) | 1.65 (1.51 to 1.8)^*^ |  | 278.29 (241.86-321.45) | 1.65 (1.46 to 1.83)^*^ |
| 75-79 years | 6326.83 (5255.49-7646.51) | 0.68 (0.63 to 0.72)^*^ |  | 25.1 (21.73-29.55) | 1.93 (1.63 to 2.23)^*^ |  | 404.54 (350.94-475.03) | 1.91 (1.62 to 2.2)^*^ |
| 80-84 years | 5674.59 (4694.58-7114.77) | 0.63 (0.6 to 0.66)^*^ |  | 44.54 (36.92-50.77) | 2.31 (1.99 to 2.63)^*^ |  | 558.52 (464.64-635.61) | 2.29 (1.97 to 2.61)^*^ |
| 85-89 years | 5049.92 (4097.47-6033.66) | 0.55 (0.5 to 0.6)^*^ |  | 85.34 (68.17-98.29) | 2 (1.52 to 2.49)^*^ |  | 847.35 (677.76-975.06) | 1.98 (1.51 to 2.46)^*^ |
| 90-94 years | 4919.64 (3885.13-5941.42) | 0.82 (0.77 to 0.86)^*^ |  | 168.31 (127.33-198.84) | 2.79 (2.4 to 3.17)^*^ |  | 1452.34 (1099.39-1715.55) | 2.77 (2.38 to 3.17)^*^ |
| 95+ years | 4748.89 (3710.49-6236.7) | 1.43 (1.35 to 1.52)^*^ |  | 322.71 (234.08-389.36) | 3.81 (3.52 to 4.09)^*^ |  | 2602.8 (1891.12-3134.33) | 3.81 (3.52 to 4.1)^*^ |
| **Low-middle SDI** | | | | | | | | |
| 65-69 years | 13031.92 (10589.08-15603.11) | 0.37 (0.34 to 0.39)^*^ |  | 17.02 (12.63-21.06) | 0.84 (0.36 to 1.33)* |  | 421.4 (313.65-520.42) | 0.82 (0.33 to 1.31)^*^ |
| 70-74 years | 11415.42 (9357.61-13919.06) | 0.5 (0.49 to 0.52)^*^ |  | 26.71 (19.65-32.48) | 1.13 (0.61 to 1.65)^*^ |  | 541.96 (401.33-657.41) | 1.12 (0.61 to 1.64)^*^ |
| 75-79 years | 9897.55 (8055.98-11923.54) | 0.53 (0.49 to 0.56)^*^ |  | 42.14 (32.22-52.43) | 1.31 (0.8 to 1.83)^*^ |  | 679.94 (521.38-845.71) | 1.3 (0.78 to 1.83)^*^ |
| 80-84 years | 8491 (6924.97-10471.66) | 0.57 (0.54 to 0.61)^*^ |  | 63.29 (49.85-76.05) | 1.79 (1.02 to 2.56)^*^ |  | 796.89 (627.98-958.36) | 1.78 (1.01 to 2.55)^*^ |
| 85-89 years | 7081.49 (5658.18-8579.03) | 0.67 (0.62 to 0.71)^*^ |  | 99.72 (76.86-120.97) | 2.08 (1.28 to 2.89)^*^ |  | 995.81 (768.1-1207.35) | 2.07 (1.27 to 2.87)^*^ |
| 90-94 years | 6391.99 (4735.66-7945.23) | 0.79 (0.72 to 0.86)^*^ |  | 171.31 (131.03-211.79) | 2.57 (1.63 to 3.52)^*^ |  | 1479.58 (1132.53-1828.48) | 2.56 (1.62 to 3.51)^*^ |
| 95+ years | 6700.2 (4791.96-9179.07) | 1.19 (1.12 to 1.26)^*^ |  | 264.84 (185.59-342.21) | 2.58 (1.75 to 3.42)^*^ |  | 2121.58 (1494.48-2749.21) | 2.57 (1.53 to 3.61)^*^ |
| **Low SDI** | | | | | | | | |
| 65-69 years | 10313.28 (8384.94-12395.2) | 0.41 (0.38 to 0.43)^*^ |  | 19.91 (15.46-24.27) | -0.14 (-0.46 to 0.19) |  | 489.92 (381.95-595.81) | -0.13 (-0.49 to 0.24) |
| 70-74 years | 9128 (7434.39-11244.09) | 0.53 (0.51 to 0.55)^*^ |  | 28.48 (20.47-35.35) | 0.34 (0.02 to 0.66)^*^ |  | 576.57 (417.36-714.65) | 0.34 (0.02 to 0.66)^*^ |
| 75-79 years | 7693.95 (6267.91-9450.64) | 0.51 (0.46 to 0.55)^*^ |  | 50.47 (38.94-62.03) | 0.47 (0.08 to 0.87)^*^ |  | 813.61 (628.67-999.11) | 0.47 (0.09 to 0.86)^*^ |
| 80-84 years | 6216.1 (4962.18-8060.08) | 0.43 (0.35 to 0.51)^*^ |  | 69.16 (53.38-86.25) | 0.84 (0.46 to 1.22)^*^ |  | 871.08 (672.97-1084.97) | 0.83 (0.45 to 1.2)^*^ |
| 85-89 years | 4409.83 (3553.5-5425.26) | 0.33 (0.23 to 0.42)^*^ |  | 73.85 (54.12-94.19) | 1.25 (0.49 to 2.01)^*^ |  | 739.74 (542.41-943.69) | 1.24 (0.47 to 2.02)^*^ |
| 90-94 years | 3265.59 (2527.8-4102.56) | 0.17 (0.05 to 0.29)^*^ |  | 135.16 (99.76-172.79) | 1.45 (0.42 to 2.49)^*^ |  | 1167.44 (862.09-1491.79) | 1.44 (0.42 to 2.48)^*^ |
| 95+ years | 3119.2 (2382.21-4123) | 0.22 (0.11 to 0.32)^*^ |  | 298.44 (212.16-398.46) | 1.71 (1.15 to 2.26)^*^ |  | 2462.85 (1751.73-3288.06) | 1.71 (1.15 to 2.27)^*^ |
| **Central Asia** | | | | | | | | |
| 65-69 years | 10939.66 (8750.61-13537.56) | 0.28 (0.21 to 0.34)^*^ |  | 9.39 (8.07-10.83) | 0.56 (-0.42 to 1.54) |  | 235.3 (203.9-270.3) | 0.54 (-0.42 to 1.51) |
| 70-74 years | 8733.48 (6941.51-10809.7) | 0.27 (0.22 to 0.32)^*^ |  | 14.48 (12.09-17.15) | 2.02 (0.79 to 3.26)^*^ |  | 296.5 (248.1-349.21) | 2 (0.81 to 3.21)^*^ |
| 75-79 years | 6643.48 (5389.03-8106.59) | 0.23 (0.19 to 0.28)^*^ |  | 16.77 (14.53-18.92) | 2 (1.16 to 2.85)^*^ |  | 269.31 (233.77-303.14) | 1.93 (1.11 to 2.77)^*^ |
| 80-84 years | 4846.79 (3855.84-5960.32) | 0.18 (0.14 to 0.22)^*^ |  | 22.35 (18.76-25.97) | 2.88 (1.6 to 4.17)^*^ |  | 281.99 (237.5-327.45) | 2.83 (1.48 to 4.19)^*^ |
| 85-89 years | 3375.88 (2767.32-4051.33) | 0.07 (0.03 to 0.11)^*^ |  | 30.25 (24.77-34.97) | 2.82 (1.19 to 4.48)^*^ |  | 302.99 (248.39-349.99) | 2.81 (1.21 to 4.44)^*^ |
| 90-94 years | 2408.2 (1877.3-2911.72) | -0.03 (-0.08 to 0.02) |  | 42.99 (33.6-50.73) | 1.9 (0.6 to 3.23)^*^ |  | 371.68 (290.64-438.75) | 1.89 (0.59 to 3.21)^*^ |
| 95+ years | 1972.85 (1516.66-2451.14) | -0.07 (-0.1 to -0.03)^*^ |  | 68.18 (47.5-84.1) | 0.95 (-0.4 to 2.32) |  | 555.76 (387.54-685.39) | 0.91 (-0.5 to 2.35) |
| **Central Europe** | | | | | | | | |
| 65-69 years | 9237.83 (7277.82-11276.58) | -0.26 (-0.27 to -0.25)^*^ |  | 6.66 (5.68-8.07) | -1.66 (-2.75 to -0.56)^*^ |  | 167.17 (143.42-202.32) | -1.62 (-2.58 to -0.65)^*^ |
| 70-74 years | 10162.86 (8309.02-12386.91) | -0.09 (-0.12 to -0.07)^*^ |  | 11.46 (9.86-13.68) | -0.92 (-1.6 to -0.23)^*^ |  | 234.78 (204.01-278.79) | -0.91 (-1.55 to -0.26)^*^ |
| 75-79 years | 11124.89 (9120.61-13863.44) | 0.09 (0.04 to 0.14)^*^ |  | 20.63 (17.94-24.22) | -0.79 (-1.78 to 0.21) |  | 335.08 (292.73-391.65) | -0.73 (-1.64 to 0.18) |
| 80-84 years | 11051.48 (9140.94-14097.29) | 0.25 (0.18 to 0.32)^*^ |  | 38.76 (33.66-44.25) | 0.84 (-0.4 to 2.09) |  | 486.81 (424.8-555.45) | 0.73 (0.08 to 1.38)^*^ |
| 85-89 years | 9951.56 (7765.12-12301.54) | 0.4 (0.32 to 0.48)^*^ |  | 75.36 (64.09-84) | 2.58 (1.62 to 3.54)^*^ |  | 749.04 (639.06-834.12) | 2.53 (1.58 to 3.48)^*^ |
| 90-94 years | 8536.56 (6347.97-10641.15) | 0.44 (0.35 to 0.53)^*^ |  | 121.19 (97.66-135.04) | 2.26 (1.15 to 3.39)^*^ |  | 1048.91 (846.2-1167.57) | 2.25 (1.16 to 3.35)^*^ |
| 95+ years | 6692.19 (4809.09-8706.92) | 0.31 (0.26 to 0.36)^*^ |  | 177.77 (136.76-202.14) | 2.5 (0.47 to 4.57)^*^ |  | 1456.98 (1122.2-1655.61) | 2.49 (0.44 to 4.58)^*^ |
| **Eastern Europe** | | | | | | | | |
| 65-69 years | 13094.04 (10561.11-15304.83) | 0.11 (0.05 to 0.17)^*^ |  | 15.02 (13.12-17.07) | 0.64 (-0.29 to 1.58) |  | 373.36 (327.44-423.83) | 0.6 (-0.28 to 1.49) |
| 70-74 years | 10507.07 (8632.7-12251.37) | 0.12 (0.09 to 0.15)^*^ |  | 23.3 (19.89-27.77) | 1.69 (0.43 to 2.97)^*^ |  | 472.65 (404.05-561.26) | 1.58 (0.55 to 2.63)^*^ |
| 75-79 years | 8186.38 (6960.92-9636.42) | 0.32 (0.22 to 0.42)^*^ |  | 34.06 (30.7-36.88) | 2.57 (1.86 to 3.28)^*^ |  | 542.75 (490.18-586.56) | 2.5 (1.81 to 3.2)^*^ |
| 80-84 years | 5990.24 (4960.58-7471.5) | 0.49 (0.39 to 0.6)^*^ |  | 52.55 (45.58-58.05) | 4.6 (3.69 to 5.51)^*^ |  | 656.35 (569.99-724.46) | 4.57 (3.57 to 5.57)^*^ |
| 85-89 years | 3689.28 (2963.94-4404.81) | 0.33 (0.21 to 0.45)^*^ |  | 65.4 (56.28-73.84) | 5.95 (4.39 to 7.54)^*^ |  | 651.13 (560.22-734.79) | 5.89 (4.33 to 7.47)^*^ |
| 90-94 years | 2352.09 (1725.99-2883.79) | 0.3 (0.21 to 0.39)^*^ |  | 118.62 (95.9-133.58) | 5.73 (3.99 to 7.49)^*^ |  | 1023.08 (827.8-1151.71) | 5.71 (3.97 to 7.47)^*^ |
| 95+ years | 1969.84 (1338.01-2738.08) | 0.53 (0.44 to 0.62)^*^ |  | 177.61 (136.98-200.3) | 5.48 (2.47 to 8.58)^*^ |  | 1445.34 (1116.04-1629.61) | 5.43 (2.38 to 8.58)^*^ |
| **Australasia** | | | | | | | | |
| 65-69 years | 10396.81 (7166.49-14211.18) | -0.34 (-0.5 to -0.17)^*^ |  | 2.65 (2.3-3.08) | -0.87 (-2.43 to 0.71) |  | 70.72 (61.07-82.03) | -1.09 (-2.7 to 0.55) |
| 70-74 years | 10936.67 (7635.77-14609.13) | -0.24 (-0.43 to -0.04)^*^ |  | 5.52 (4.62-6.46) | -1.47 (-3.41 to 0.5) |  | 116.05 (97.81-135.99) | -1.41 (-3.26 to 0.47) |
| 75-79 years | 13995.61 (9779.5-18653.33) | 0.24 (0.2 to 0.28)^*^ |  | 15.16 (12.4-17.84) | 0.12 (-1.51 to 1.77) |  | 249.11 (205.44-292.33) | 0.11 (-1.48 to 1.73) |
| 80-84 years | 15398.75 (11262.29-20402.26) | 0.43 (0.4 to 0.47)^*^ |  | 36.97 (27.83-45.21) | 0.95 (-0.64 to 2.56) |  | 466.57 (355.03-568.71) | 0.92 (-0.67 to 2.53) |
| 85-89 years | 15012.26 (11077.67-19341.32) | 0.51 (0.46 to 0.56)^*^ |  | 92.83 (68.35-113.63) | 1.93 (0.41 to 3.48)^*^ |  | 919.57 (682.51-1123.35) | 1.89 (0.4 to 3.39)^*^ |
| 90-94 years | 13743.19 (10440.29-17234.82) | 0.59 (0.54 to 0.64)^*^ |  | 224.67 (161.79-272.61) | 2.66 (0.69 to 4.68)^*^ |  | 1940.45 (1401.61-2352.97) | 2.65 (0.69 to 4.64)^*^ |
| 95+ years | 11583.8 (9383.54-14792.57) | 0.7 (0.64 to 0.75)^*^ |  | 438.08 (311.35-524.3) | 2.84 (0.61 to 5.12)^*^ |  | 3538.12 (2517.96-4232.87) | 2.81 (0.56 to 5.11)^*^ |
| **High-income Asia Pacific** | | | | | | | | |
| 65-69 years | 6831.27 (5351.71-8423.61) | 0.13 (0.03 to 0.23)^*^ |  | 2.05 (1.76-2.35) | 0.29 (-0.9 to 1.49) |  | 53.94 (46.62-61.07) | 0.27 (-0.79 to 1.34) |
| 70-74 years | 7032.8 (5517.03-8735.85) | 0.29 (0.2 to 0.38)^*^ |  | 4.27 (3.55-5.18) | 0.04 (-0.92 to 1) |  | 88.81 (74.48-106.75) | 0.04 (-0.86 to 0.94) |
| 75-79 years | 8091.77 (5947.87-10662.21) | 0.33 (0.21 to 0.45)^*^ |  | 10.4 (8.21-12.16) | 0.01 (-0.9 to 0.93) |  | 168.13 (133.82-197.08) | 0 (-0.86 to 0.85) |
| 80-84 years | 9335.84 (6758.9-12414.09) | 0.47 (0.31 to 0.63)^*^ |  | 22.72 (16.45-28.69) | 0.54 (-0.17 to 1.25) |  | 286.42 (209.21-359.34) | 0.51 (-0.18 to 1.19) |
| 85-89 years | 10737.16 (7283.9-14296.65) | 0.68 (0.46 to 0.9)^*^ |  | 54.18 (36.35-68.25) | 1.09 (0.29 to 1.89)^*^ |  | 536.96 (363-675.99) | 1.06 (0.27 to 1.85)^*^ |
| 90-94 years | 11850.92 (7911.2-15634.06) | 0.96 (0.79 to 1.12)^*^ |  | 129.59 (84.95-159.06) | 2.24 (1.74 to 2.74)^*^ |  | 1121.24 (737.3-1375.08) | 2.26 (1.78 to 2.75)^*^ |
| 95+ years | 12680.15 (8639.72-17999.82) | 1.32 (1.23 to 1.42)^*^ |  | 289.83 (181.38-360.32) | 3.25 (2.62 to 3.89)^*^ |  | 2264.39 (1420.27-2816.98) | 3.13 (2.51 to 3.77)^*^ |
| **High-income North America** | | | | | | | | |
| 65-69 years | 16702.2 (12993.61-20635.16) | 0.12 (0 to 0.24) |  | 7.39 (6.85-7.92) | 0.9 (0.46 to 1.33)^*^ |  | 189.48 (175.83-203.23) | 0.86 (0.46 to 1.26)^*^ |
| 70-74 years | 20516.93 (16791.61-24902.45) | 0.32 (0.09 to 0.55)^*^ |  | 14.77 (13.5-16.07) | 0.76 (0.21 to 1.31)^*^ |  | 305.91 (279.67-332.91) | 0.74 (0.24 to 1.24)^*^ |
| 75-79 years | 26206.21 (20873.27-32603.97) | 0.48 (0.17 to 0.78)^*^ |  | 30.84 (27.16-33.16) | 0.33 (-0.33 to 0.99) |  | 505.33 (447.58-542.18) | 0.34 (-0.29 to 0.98) |
| 80-84 years | 31262.38 (26069.92-38208.74) | 0.49 (0.15 to 0.84)^*^ |  | 57.03 (45.64-65.09) | 0.22 (-0.38 to 0.82) |  | 726.87 (586.85-825.9) | 0.2 (-0.6 to 1) |
| 85-89 years | 35685.66 (28129.91-44596.93) | 0.42 (0.06 to 0.78)^*^ |  | 101.27 (77.12-114.58) | 0.06 (-0.53 to 0.66) |  | 1016.5 (778.17-1146.02) | 0.05 (-0.54 to 0.65) |
| 90-94 years | 36948.3 (27749.83-45999.5) | 0.37 (-0.05 to 0.79) |  | 182.79 (133.48-209.63) | 0.24 (-0.51 to 1) |  | 1592.7 (1168.56-1825.8) | 0.24 (-0.5 to 0.99) |
| 95+ years | 35106.63 (24071.77-46711.77) | 0.23 (-0.04 to 0.5) |  | 317.64 (224.68-368.49) | 0.48 (-0.37 to 1.33) |  | 2595.66 (1838.82-3005.59) | 0.48 (-0.41 to 1.39) |
| **Southern Latin America** | | | | | | | | |
| 65-69 years | 13760.2 (10979.66-16624.15) | 0.57 (0.49 to 0.64)^*^ |  | 16.42 (14.43-18.69) | 4.35 (2.94 to 5.78)^*^ |  | 407.43 (359.47-463.34) | 4.21 (2.87 to 5.57)^*^ |
| 70-74 years | 13789.64 (10930.53-16593.02) | 0.74 (0.68 to 0.79)^*^ |  | 31.85 (28.01-36.04) | 4.05 (2.55 to 5.57)^*^ |  | 643.96 (565.83-725.37) | 3.98 (2.53 to 5.45)^*^ |
| 75-79 years | 14246.56 (11207.35-17571.28) | 0.75 (0.61 to 0.89)^*^ |  | 70.72 (61.23-80.9) | 4.43 (2.7 to 6.18)^*^ |  | 1135.17 (985.42-1297.69) | 4.42 (2.8 to 6.07)^*^ |
| 80-84 years | 13907.05 (10801.01-17694.36) | 0.88 (0.8 to 0.96)^*^ |  | 137.79 (113.37-161.99) | 4.79 (3.72 to 5.87)^*^ |  | 1720.08 (1416.4-2017.86) | 4.74 (3.68 to 5.81)^*^ |
| 85-89 years | 12793.84 (10333.84-15602.06) | 1.09 (1.01 to 1.18)^*^ |  | 297.08 (237.65-343.92) | 5.04 (4.22 to 5.87)^*^ |  | 2935.47 (2351.64-3395.39) | 5 (4.19 to 5.82)^*^ |
| 90-94 years | 11785.28 (9735.01-14179.77) | 1.36 (1.23 to 1.49)^*^ |  | 515 (406.66-601.21) | 5.22 (3.7 to 6.77)^*^ |  | 4441.81 (3508.35-5182.78) | 5.21 (3.69 to 6.75)^*^ |
| 95+ years | 10612.87 (8637.75-13006.84) | 1.56 (1.46 to 1.66)^*^ |  | 878.69 (663.62-1015.65) | 5.82 (4.21 to 7.45)^*^ |  | 7185.62 (5429.25-8303.99) | 5.79 (4.21 to 7.4)^*^ |
| **Western Europe** | | | | | | | | |
| 65-69 years | 7126.37 (5644.8-8554.53) | -0.47 (-0.57 to -0.38)^*^ |  | 3.65 (3.31-4.02) | 1.08 (0.48 to 1.68)^*^ |  | 93.02 (84.67-102.38) | 0.99 (0.42 to 1.56)^*^ |
| 70-74 years | 5882.38 (4662.21-7240.79) | -0.45 (-0.56 to -0.34)^*^ |  | 7.74 (6.86-8.64) | 1.07 (0.39 to 1.76)^*^ |  | 157.43 (139.88-175.07) | 1.02 (0.36 to 1.68)^*^ |
| 75-79 years | 5390.82 (4315.51-6266.02) | -0.19 (-0.33 to -0.05)^*^ |  | 19.82 (16.94-22.24) | 1.6 (0.97 to 2.22)^*^ |  | 317.93 (272.1-356.32) | 1.58 (0.98 to 2.17)^*^ |
| 80-84 years | 5059.3 (4118.08-6036.08) | 0.38 (0.28 to 0.48)^*^ |  | 41.83 (33.41-47.97) | 2.02 (1.35 to 2.7)^*^ |  | 519.96 (416.27-595.52) | 2 (1.32 to 2.67)^*^ |
| 85-89 years | 4658.59 (3589.03-5935.4) | 1.29 (1.08 to 1.51)^*^ |  | 97.42 (73.14-113.53) | 2.71 (1.87 to 3.55)^*^ |  | 959.58 (721.29-1118.14) | 2.69 (1.9 to 3.48)^*^ |
| 90-94 years | 4381.7 (3182.7-5815.18) | 2.01 (1.92 to 2.1)^*^ |  | 219.81 (162.74-251.65) | 3.32 (2.48 to 4.16)^*^ |  | 1894.06 (1402.8-2168.66) | 3.31 (2.47 to 4.15)^*^ |
| 95+ years | 4313.41 (2772.94-6190.98) | 2.38 (2.21 to 2.54)^*^ |  | 395.53 (286.11-457.17) | 3.7 (3.15 to 4.25)^*^ |  | 3202.71 (2318.16-3700.87) | 3.68 (3.11 to 4.25)^*^ |
| **Andean Latin America** | | | | | | | | |
| 65-69 years | 17331.43 (13585.28-21154.84) | 0.4 (0.32 to 0.49)^*^ |  | 10.94 (6.77-15.19) | 1.62 (0.37 to 2.89)^*^ |  | 277.11 (175.61-382.84) | 1.57 (0.39 to 2.76)^*^ |
| 70-74 years | 15688.98 (12188.69-19278.31) | 0.45 (0.39 to 0.51)^*^ |  | 19.83 (12.2-28.92) | 1.9 (0.67 to 3.15)^*^ |  | 406.61 (253.91-586.82) | 1.86 (0.63 to 3.11)^*^ |
| 75-79 years | 14903.71 (11536.75-18616.8) | 0.55 (0.44 to 0.65)^*^ |  | 38.59 (23.79-54.87) | 1.92 (0.94 to 2.91)^*^ |  | 626.16 (391.17-885.16) | 1.9 (1.09 to 2.71)^*^ |
| 80-84 years | 13948.62 (10960.44-18073.38) | 0.59 (0.51 to 0.67)^*^ |  | 69.25 (45.08-97.2) | 2.15 (0.56 to 3.76)^*^ |  | 874.21 (572.65-1223.16) | 2.15 (0.57 to 3.75)^*^ |
| 85-89 years | 12629.04 (10073.02-15830.29) | 0.52 (0.47 to 0.57)^*^ |  | 144.7 (95.74-205.6) | 1.22 (0.41 to 2.04)^*^ |  | 1437.53 (953.56-2038.67) | 1.22 (0.4 to 2.05)^*^ |
| 90-94 years | 11361.12 (8961.01-14586.26) | 0.49 (0.45 to 0.53)^*^ |  | 247.8 (171.34-348.51) | 1.12 (0.3 to 1.95)^*^ |  | 2141.06 (1483.85-3007.58) | 1.11 (0.3 to 1.94)^*^ |
| 95+ years | 9980.32 (7603.48-13885.96) | 0.43 (0.39 to 0.46)^*^ |  | 376.98 (245.08-536.24) | 1.41 (0.7 to 2.14)^*^ |  | 3102.6 (2020.86-4408.29) | 1.48 (0.42 to 2.55)^*^ |
| **Caribbean** | | | | | | | | |
| 65-69 years | 9869.42 (8015.42-12027.32) | 0.03 (0.01 to 0.06)^*^ |  | 5.86 (4.87-7.06) | 2.83 (2.24 to 3.43)^*^ |  | 148.71 (125.45-179.02) | 2.65 (2.1 to 3.21)^*^ |
| 70-74 years | 8421.33 (6839.7-10263.73) | 0.06 (0.04 to 0.09)^*^ |  | 10.03 (8.44-12.11) | 2.74 (1.97 to 3.52)^*^ |  | 205.4 (174-246.85) | 2.63 (1.89 to 3.38)^*^ |
| 75-79 years | 7775.12 (6157.23-9647.89) | 0.08 (0.05 to 0.1)^*^ |  | 19.17 (16.25-22.79) | 2.99 (2.33 to 3.67)^*^ |  | 310.74 (263.33-368.43) | 2.92 (2.27 to 3.58)^*^ |
| 80-84 years | 6977.12 (5501.82-9115.55) | 0.05 (0.01 to 0.09)^*^ |  | 35.98 (30.52-41.16) | 3.21 (2.54 to 3.9)^*^ |  | 452.18 (384.28-516.98) | 3.17 (2.5 to 3.84)^*^ |
| 85-89 years | 6052.02 (4616.42-7830.64) | -0.03 (-0.07 to 0.01) |  | 72.77 (58.31-87.82) | 3 (2.34 to 3.67)^*^ |  | 721.42 (578.44-870.98) | 2.95 (2.3 to 3.61)^*^ |
| 90-94 years | 5077.12 (3743.43-6441.63) | -0.08 (-0.1 to -0.07)^*^ |  | 114.16 (90.89-133.31) | 2.65 (1.38 to 3.93)^*^ |  | 985.64 (784.62-1149.39) | 2.63 (1.36 to 3.91)^*^ |
| 95+ years | 4231.26 (3071.3-5551.69) | 0.11 (0.07 to 0.15)^*^ |  | 179.63 (127.52-232.21) | 2.83 (1.34 to 4.35)^*^ |  | 1377.2 (981.68-1766.35) | 2.64 (1.11 to 4.2)^*^ |
| **Central Latin America** | | | | | | | | |
| 65-69 years | 12968.83 (10483.06-15673.51) | 0.49 (0.37 to 0.62)^*^ |  | 19.05 (16.12-23.08) | 3.37 (2.77 to 3.96)^*^ |  | 472.27 (400.48-570.12) | 3.29 (2.72 to 3.88)^*^ |
| 70-74 years | 11451.23 (9411.82-13804.01) | 0.59 (0.44 to 0.74)^*^ |  | 28.86 (25.25-33.68) | 2.91 (2.37 to 3.45)^*^ |  | 584.21 (512.02-681.19) | 2.88 (2.35 to 3.41)^*^ |
| 75-79 years | 10836.37 (9088.13-12938.9) | 0.8 (0.59 to 1.01)^*^ |  | 47.45 (41.69-54.57) | 2.61 (2.08 to 3.15)^*^ |  | 764.91 (671.62-878.37) | 2.59 (2.06 to 3.12)^*^ |
| 80-84 years | 9904.9 (8228.27-12540.28) | 0.78 (0.57 to 0.98)^*^ |  | 75.24 (64.82-84.18) | 2.59 (1.96 to 3.23)^*^ |  | 944.07 (813.57-1056.47) | 2.61 (1.98 to 3.23)^*^ |
| 85-89 years | 8700.34 (6979.64-10576.01) | 0.52 (0.34 to 0.7)^*^ |  | 125.41 (105.27-141.61) | 0.73 (0.2 to 1.26)^*^ |  | 1245.48 (1046.68-1404.57) | 0.73 (0.21 to 1.25)^*^ |
| 90-94 years | 7661.82 (6016.82-9378.12) | 0.44 (0.28 to 0.59)^*^ |  | 183.16 (152-205.08) | 1.64 (0.77 to 2.51)^*^ |  | 1581.68 (1313.75-1770.59) | 1.62 (0.76 to 2.49)^*^ |
| 95+ years | 6534.46 (4997.01-8519.28) | 0.3 (0.23 to 0.36)^*^ |  | 199.4 (148.03-240.69) | 2.85 (1.81 to 3.9)^*^ |  | 1593.63 (1190.06-1927.16) | 3.07 (2.26 to 3.89)^*^ |
| **Tropical Latin America** | | | | | | | | |
| 65-69 years | 16487.71 (13321.78-19643.42) | 0.11 (0.07 to 0.16)^*^ |  | 23.82 (21.41-25.81) | 3.89 (2.79 to 5)^*^ |  | 589.63 (531.89-638) | 3.77 (2.72 to 4.84)^*^ |
| 70-74 years | 16754.86 (14026.48-19917.97) | 0.36 (0.3 to 0.42)^*^ |  | 45.06 (40.7-48.79) | 3.75 (2.69 to 4.81)^*^ |  | 910.8 (825.46-984.98) | 3.69 (2.65 to 4.74)^*^ |
| 75-79 years | 18016.64 (14795.06-22195.99) | 0.57 (0.53 to 0.61)^*^ |  | 94.07 (81.86-103.84) | 4.04 (3.37 to 4.71)^*^ |  | 1512.23 (1317.18-1667.92) | 4 (3.34 to 4.65)^*^ |
| 80-84 years | 18689.49 (15496.26-23054.02) | 0.8 (0.71 to 0.88)^*^ |  | 181.01 (148.33-202.97) | 4.28 (3.6 to 4.95)^*^ |  | 2265.74 (1856.06-2538.15) | 4.24 (3.57 to 4.91)^*^ |
| 85-89 years | 18829.03 (15003.07-22511.46) | 1.08 (1.04 to 1.12)^*^ |  | 364.55 (277.89-420.96) | 4.91 (4.14 to 5.69)^*^ |  | 3612.39 (2759.11-4169.34) | 4.87 (4.12 to 5.63)^*^ |
| 90-94 years | 17987.86 (13842.16-21892.79) | 1.37 (1.3 to 1.45)^*^ |  | 640.8 (468.17-745.52) | 5.22 (3.96 to 6.49)^*^ |  | 5527.66 (4040.65-6430.85) | 5.2 (3.95 to 6.46)^*^ |
| 95+ years | 15725.87 (11734.96-21243.84) | 1.65 (1.55 to 1.74)^*^ |  | 1022.02 (712.1-1219.24) | 6.11 (5.01 to 7.22)^*^ |  | 8222.3 (5748.85-9792.65) | 6.09 (4.97 to 7.22)^*^ |
| **North Africa and Middle East** | | | | | | | | |
| 65-69 years | 4014.03 (3234.02-4914.1) | 0.17 (0.15 to 0.19)^*^ |  | 4.42 (3.52-5.68) | 0.1 (-0.04 to 0.25) |  | 110.09 (87.82-140.07) | 0.11 (-0.03 to 0.25) |
| 70-74 years | 3494.46 (2805.41-4259.32) | 0.2 (0.19 to 0.22)^*^ |  | 8.88 (6.7-11.54) | 0.2 (0.04 to 0.36)^*^ |  | 179.82 (135.39-233.83) | 0.2 (0.04 to 0.36)^*^ |
| 75-79 years | 3389.03 (2758.69-4134.86) | 0.29 (0.23 to 0.34)^*^ |  | 18.89 (14.63-24.43) | 0.39 (0.19 to 0.59)^*^ |  | 303.41 (235.47-392.12) | 0.39 (0.19 to 0.59)^*^ |
| 80-84 years | 3061.67 (2516.26-3838.88) | 0.33 (0.3 to 0.36)^*^ |  | 38.85 (30.07-49.78) | 0.46 (0.22 to 0.71)^*^ |  | 485.97 (376.48-622.1) | 0.45 (0.21 to 0.7)^*^ |
| 85-89 years | 2506.54 (2042.62-3031.12) | 0.31 (0.26 to 0.36)^*^ |  | 82.38 (61.14-103.13) | 1.46 (0.93 to 1.99)^*^ |  | 818.93 (607.95-1024.74) | 1.42 (0.86 to 1.98)^*^ |
| 90-94 years | 2165.05 (1791.65-2633.98) | 0.32 (0.29 to 0.35)^*^ |  | 120.23 (89.23-151.76) | 1.75 (1.36 to 2.14)^*^ |  | 1038.09 (770.5-1310.31) | 1.71 (1.36 to 2.05)^*^ |
| 95+ years | 2027.03 (1639.63-2628.85) | 0.39 (0.34 to 0.43)^*^ |  | 171.29 (115.07-238.26) | 1.17 (0.4 to 1.94)^*^ |  | 1404.89 (944.37-1953.69) | 1.18 (0.33 to 2.05)^*^ |
| **South Asia** | | | | | | | | |
| 65-69 years | 16856.54 (13737.24-20311.08) | 0.37 (0.35 to 0.4)^*^ |  | 20.37 (15.03-25.33) | 0.43 (-0.38 to 1.24) |  | 504.78 (375.56-625.88) | 0.42 (-0.38 to 1.22)^*^ |
| 70-74 years | 14232.59 (11733.69-17359.46) | 0.45 (0.43 to 0.47)^*^ |  | 30.08 (21.58-36.9) | 0.77 (0.08 to 1.47)^*^ |  | 611.33 (441.81-749.42) | 0.77 (0.08 to 1.46)^*^ |
| 75-79 years | 12019.4 (9844.31-14502.03) | 0.5 (0.44 to 0.56)^*^ |  | 46.79 (35.41-58.83) | 0.89 (0.32 to 1.47)^*^ |  | 755.94 (572.83-948.3) | 0.89 (0.32 to 1.46)^*^ |
| 80-84 years | 9835.03 (8011.88-12416.62) | 0.5 (0.43 to 0.56)^*^ |  | 64.75 (50.3-79.88) | 1.31 (0.22 to 2.41)^*^ |  | 816.18 (634.31-1005.78) | 1.3 (0.21 to 2.4)^*^ |
| 85-89 years | 7610.74 (6172.83-9285.43) | 0.39 (0.34 to 0.45)^*^ |  | 96.14 (71.89-120.74) | 1.22 (0.07 to 2.37)^*^ |  | 962.55 (720.55-1208.77) | 1.2 (0.07 to 2.35)^*^ |
| 90-94 years | 6128.69 (4603.68-7696.09) | 0.35 (0.29 to 0.41)^*^ |  | 152.91 (113.54-194.62) | 1.58 (0.9 to 2.27)^*^ |  | 1321.31 (980.94-1680.62) | 1.58 (0.9 to 2.27)^*^ |
| 95+ years | 5448.6 (3907.86-7576.65) | 0.43 (0.37 to 0.49)^*^ |  | 226.87 (160.77-312.9) | 1.62 (0.13 to 3.14)^*^ |  | 1848.05 (1312.78-2550.38) | 1.6 (0.08 to 3.15)^*^ |
| **East Asia** | | | | | | | | |
| 65-69 years | 3015.06 (2418.37-3633.19) | 0.15 (0.14 to 0.16)^*^ |  | 1.16 (0.84-1.92) | -2.24 (-2.82 to -1.65)^*^ |  | 30.07 (22.35-48.6) | -2.13 (-2.69 to -1.58)^*^ |
| 70-74 years | 2482.17 (1979.94-3040.02) | 0.14 (0.07 to 0.22)^*^ |  | 2.12 (1.62-3.2) | -1.95 (-2.48 to -1.42)^*^ |  | 43.94 (33.87-65.43) | -1.89 (-2.4 to -1.38)^*^ |
| 75-79 years | 1991.6 (1575.14-2440.13) | -0.09 (-0.18 to 0)^*^ |  | 3.84 (2.92-6.15) | -1.53 (-2.11 to -0.94)^*^ |  | 62.14 (47.72-99.02) | -1.53 (-2.09 to -0.96)^*^ |
| 80-84 years | 1541.71 (1155.74-2010.93) | -0.29 (-0.32 to -0.26)^*^ |  | 7.35 (5.64-10.4) | -0.55 (-1.03 to -0.07)^*^ |  | 92.17 (70.79-129.86) | -0.58 (-1.05 to -0.12)^*^ |
| 85-89 years | 1120.23 (850.76-1379.81) | -0.47 (-0.53 to -0.41)^*^ |  | 15.37 (11.65-21.37) | -0.55 (-1.28 to 0.19) |  | 152.28 (115.56-211.52) | -0.58 (-1.3 to 0.14) |
| 90-94 years | 897.03 (660.82-1147.54) | -0.7 (-0.77 to -0.62)^*^ |  | 33.36 (23.82-44.24) | 0 (-0.82 to 0.82) |  | 287.77 (205.5-381.56) | -0.01 (-0.83 to 0.81) |
| 95+ years | 876.63 (620.35-1224.1) | -0.76 (-0.86 to -0.67)^*^ |  | 69.11 (46.31-90.05) | 0.39 (-0.58 to 1.38) |  | 538.98 (362.03-709.76) | 0.2 (-0.83 to 1.24) |
| **Oceania** | | | | | | | | |
| 65-69 years | 4369.91 (3483.12-5502.43) | 0.13 (0.11 to 0.15)^*^ |  | 6.56 (4.12-12.33) | -0.24 (-0.5 to 0.02) |  | 162.49 (103.18-304.38) | -0.24 (-0.47 to 0) |
| 70-74 years | 3724.45 (2897.61-4695.02) | 0.15 (0.1 to 0.19)^*^ |  | 7.59 (4.78-12.33) | -0.19 (-0.32 to -0.06)^*^ |  | 153.85 (97.56-248.21) | -0.2 (-0.33 to -0.08)^*^ |
| 75-79 years | 3180.03 (2488.64-3936.91) | 0.12 (0.07 to 0.16)^*^ |  | 9.72 (6.15-16.19) | -0.36 (-0.54 to -0.17)^*^ |  | 157.81 (100.32-262.34) | -0.34 (-0.61 to -0.07)^*^ |
| 80-84 years | 2789.36 (2131.59-3564.72) | 0.12 (0.11 to 0.14)^*^ |  | 25.04 (16.3-44.13) | -0.46 (-0.68 to -0.24)^*^ |  | 315.36 (205.79-554.81) | -0.46 (-0.68 to -0.25)^*^ |
| 85-89 years | 2532.16 (1980.51-3204.67) | 0.14 (0.11 to 0.16)^*^ |  | 48.25 (31.25-75.71) | -1.05 (-1.34 to -0.76)^*^ |  | 480.23 (311.35-753.16) | -1.05 (-1.33 to -0.77)^*^ |
| 90-94 years | 2319.42 (1876.68-2872.66) | 0.17 (0.14 to 0.2)^*^ |  | 59.03 (37.8-99.94) | -1.02 (-1.62 to -0.42)^*^ |  | 510.03 (327.18-863.03) | -1.03 (-1.63 to -0.43)^*^ |
| 95+ years | 2232.69 (1710.89-2899.64) | 0.36 (0.33 to 0.38)^*^ |  | 135.68 (88.92-208.34) | -0.1 (-0.69 to 0.49) |  | 1057.78 (697.77-1674.48) | -0.31 (-0.86 to 0.24) |
| **Southeast Asia** | | | | | | | | |
| 65-69 years | 7123.41 (5907.56-8585.7) | -0.09 (-0.21 to 0.03) |  | 9.67 (6.91-12.46) | 0.71 (0.59 to 0.84)^*^ |  | 239.54 (173.14-307.14) | 0.7 (0.58 to 0.81)^*^ |
| 70-74 years | 5994.66 (5021.57-7407.06) | -0.01 (-0.04 to 0.02) |  | 16.66 (11.76-21.07) | 0.98 (0.87 to 1.09)^*^ |  | 337.02 (238.95-425.44) | 0.96 (0.86 to 1.07)^*^ |
| 75-79 years | 5031.28 (4191.4-5994.35) | 0.03 (-0.06 to 0.12) |  | 30.49 (22.87-39.74) | 1.27 (1.08 to 1.46)^*^ |  | 489.9 (368.13-637.44) | 1.25 (1.1 to 1.41)^*^ |
| 80-84 years | 4269.87 (3499.06-5197.49) | 0.11 (0.04 to 0.17)^*^ |  | 53.09 (36.25-70.12) | 2.08 (1.89 to 2.27)^*^ |  | 664.28 (454.8-876.03) | 2.06 (1.88 to 2.25)^*^ |
| 85-89 years | 3620.66 (3004.93-4307.6) | 0.24 (0.14 to 0.35)^*^ |  | 97.94 (63.42-131.2) | 2.14 (1.96 to 2.33)^*^ |  | 971.43 (629.62-1301.28) | 2.11 (1.92 to 2.3)^*^ |
| 90-94 years | 3031.08 (2515.67-3637.4) | 0.47 (0.42 to 0.53)^*^ |  | 197.58 (118.49-284.58) | 3.79 (3.29 to 4.29)^*^ |  | 1703.38 (1022.47-2452.24) | 3.79 (3.49 to 4.09)^*^ |
| 95+ years | 2535.83 (2071-3251.17) | 0.57 (0.54 to 0.61)^*^ |  | 392.27 (211.32-611.05) | 4.87 (4.62 to 5.12)^*^ |  | 3120.52 (1692.84-4851.07) | 4.85 (4.5 to 5.21)^*^ |
| **Central Sub-Saharan Africa** | | | | | | | | |
| 65-69 years | 5828.48 (4652.93-7230.4) | 0.17 (0.14 to 0.2)^*^ |  | 6.7 (3.46-14.22) | -0.08 (-0.22 to 0.06) |  | 166.7 (88.21-349.98) | -0.07 (-0.19 to 0.06) |
| 70-74 years | 4983.07 (3893.59-6306.29) | 0.18 (0.16 to 0.19)^*^ |  | 10.23 (5.28-21.39) | -0.06 (-0.11 to -0.01)^*^ |  | 208.47 (108.49-432.21) | -0.06 (-0.11 to -0.01)^*^ |
| 75-79 years | 4276.4 (3422.67-5369.9) | 0.17 (0.15 to 0.19)^*^ |  | 15.91 (8.31-35.58) | 0.02 (-0.04 to 0.08) |  | 257.5 (136.7-572.9) | 0 (-0.06 to 0.05) |
| 80-84 years | 3414.57 (2660.45-4356.02) | 0.15 (0.12 to 0.18)^*^ |  | 25.43 (13.61-55.58) | 0.15 (0.04 to 0.27)^*^ |  | 321.55 (172.7-700.18) | 0.14 (0.04 to 0.24)^*^ |
| 85-89 years | 2395.2 (1962.28-2834.05) | 0.11 (0.1 to 0.11)^*^ |  | 38.16 (19.33-82.33) | 0.22 (0.12 to 0.32)^*^ |  | 381.46 (193.65-821.71) | 0.22 (0.12 to 0.32)^*^ |
| 90-94 years | 1865.89 (1548.58-2265.02) | 0.1 (0.09 to 0.11)^*^ |  | 60.04 (30.96-122.33) | 0.39 (0.31 to 0.47)^*^ |  | 518.8 (268.35-1056.32) | 0.39 (0.31 to 0.47)^*^ |
| 95+ years | 1830.77 (1464.59-2385.86) | 0.18 (0.13 to 0.22)^*^ |  | 96.74 (45.04-197.13) | 0.38 (0.32 to 0.43)^*^ |  | 801.16 (372.89-1631.38) | 0.44 (0.34 to 0.54)^*^ |
| **Eastern Sub-Saharan Africa** | | | | | | | | |
| 65-69 years | 6629.44 (5355.87-8137.84) | 0.1 (0.07 to 0.14)^*^ |  | 35.32 (27.83-45.43) | -0.33 (-0.61 to -0.04)^*^ |  | 863.49 (682.16-1110.74) | -0.32 (-0.59 to -0.06)^*^ |
| 70-74 years | 5646.25 (4581.13-6948.19) | 0.14 (0.12 to 0.15)^*^ |  | 36.08 (27.68-47.09) | -0.18 (-0.29 to -0.08)^*^ |  | 726.3 (558.48-946.97) | -0.18 (-0.31 to -0.06)^*^ |
| 75-79 years | 5095.03 (4153.42-6214.69) | 0.29 (0.27 to 0.31)^*^ |  | 92.52 (73.25-117.88) | 0.26 (0.08 to 0.44)^*^ |  | 1486.09 (1176.63-1892.67) | 0.22 (0.1 to 0.34)^*^ |
| 80-84 years | 3954.28 (3169.73-4932.86) | 0.35 (0.32 to 0.37)^*^ |  | 107.45 (81.65-136.34) | 0.77 (0.62 to 0.93)^*^ |  | 1351.36 (1027.52-1714.3) | 0.76 (0.6 to 0.91)^*^ |
| 85-89 years | 2222.57 (1778.21-2700.3) | 0.18 (0.14 to 0.21)^*^ |  | 75.29 (56.93-101.1) | 1.08 (0.99 to 1.17)^*^ |  | 751.34 (568.67-1008.82) | 1.07 (0.97 to 1.17)^*^ |
| 90-94 years | 1737.33 (1390.33-2114.17) | 0.2 (0.16 to 0.24)^*^ |  | 158.98 (112.2-219.02) | 1.45 (1.37 to 1.52)^*^ |  | 1371.7 (968.26-1889.58) | 1.44 (1.37 to 1.51)^*^ |
| 95+ years | 2499.76 (1970.62-3201.81) | 0.55 (0.51 to 0.58)^*^ |  | 547.88 (371.05-743.52) | 1.31 (1.21 to 1.41)^*^ |  | 4521.18 (3062.56-6134.81) | 1.34 (1.24 to 1.44)^*^ |
| **Southern Sub-Saharan Africa** | | | | | | | | |
| 65-69 years | 4874.75 (3884.86-6124.32) | -0.14 (-0.37 to 0.09) |  | 2.83 (1.75-4.58) | 1.38 (0.8 to 1.97)^*^ |  | 71.96 (45.12-114.88) | 1.31 (0.77 to 1.85)^*^ |
| 70-74 years | 3660.4 (2889.43-4550.47) | -0.19 (-0.47 to 0.09) |  | 4.09 (2.09-6.69) | 1.27 (1.04 to 1.5)^*^ |  | 84.24 (43.87-137.3) | 1.21 (0.99 to 1.42)^*^ |
| 75-79 years | 3123.56 (2504.94-3762.23) | -0.1 (-0.24 to 0.03) |  | 7.54 (4.3-12.99) | 0.96 (0.62 to 1.3)^*^ |  | 122.62 (71.18-209.26) | 0.95 (0.62 to 1.28)^*^ |
| 80-84 years | 2674.61 (2172.1-3363.74) | 0.01 (-0.03 to 0.05) |  | 14.3 (6.98-24.76) | 0.83 (0.53 to 1.13)^*^ |  | 180.56 (88.85-310.94) | 0.82 (0.46 to 1.17)^*^ |
| 85-89 years | 2333.44 (1876.69-2813.08) | 0.1 (0.08 to 0.13)^*^ |  | 30.49 (16.24-51.51) | 1.03 (0.39 to 1.68)^*^ |  | 304.53 (162.69-513.14) | 1.01 (0.46 to 1.57)^*^ |
| 90-94 years | 1967.41 (1515.88-2449.95) | 0.18 (0.16 to 0.21)^*^ |  | 45.97 (24.29-79.23) | 0.47 (0.07 to 0.87)^*^ |  | 397.81 (210.91-684.7) | 0.48 (0.08 to 0.88)^*^ |
| 95+ years | 1587.15 (1134.4-2216.31) | 0.25 (0.14 to 0.37)^*^ |  | 34.7 (14.19-66.27) | -1.09 (-1.47 to -0.71)^*^ |  | 288.37 (118.87-549.69) | -1.02 (-1.41 to -0.63)^*^ |
| **Western Sub-Saharan Africa** | | | | | | | | |
| 65-69 years | 7780.12 (6239.22-9574.82) | 0.11 (0.08 to 0.14)^*^ |  | 3.07 (1.96-5.89) | 0.29 (0.08 to 0.5)^*^ |  | 79.44 (52.07-148.33) | 0.28 (0.14 to 0.42)^*^ |
| 70-74 years | 7173.31 (5746.32-9030.62) | 0.12 (0.09 to 0.15)^*^ |  | 17.53 (13.25-24.91) | -0.03 (-0.13 to 0.07) |  | 356.11 (269.72-503.51) | -0.01 (-0.11 to 0.08) |
| 75-79 years | 5762.6 (4753.21-6983.53) | 0.13 (0.11 to 0.15)^*^ |  | 6.15 (4.36-11.4) | -0.01 (-0.18 to 0.16) |  | 101.67 (72.89-185.41) | -0.01 (-0.17 to 0.15) |
| 80-84 years | 4356.94 (3516.44-5540.73) | 0.1 (0.09 to 0.1)^*^ |  | 37.17 (28.35-56.89) | 0.25 (0.13 to 0.37)^*^ |  | 468.9 (357.55-716.27) | 0.25 (0.13 to 0.37)^*^ |
| 85-89 years | 2939.35 (2334.81-3600.96) | 0.03 (0.02 to 0.05)^*^ |  | 17.93 (12.17-33.56) | 0.41 (0.34 to 0.48)^*^ |  | 179.95 (122.32-335.67) | 0.41 (0.3 to 0.51)^*^ |
| 90-94 years | 2281.09 (1770.54-2804.67) | 0.01 (-0.01 to 0.03) |  | 87.7 (61.18-143.92) | 0.52 (0.43 to 0.61)^*^ |  | 757.13 (528.79-1241.58) | 0.52 (0.43 to 0.61)^*^ |
| 95+ years | 2412.97 (1842.69-3255.97) | 0.11 (0.07 to 0.14)^*^ |  | 136.87 (88.31-240.18) | 0.69 (0.59 to 0.79)^*^ |  | 1126.66 (727.62-1976.29) | 0.71 (0.61 to 0.82)^*^ |

Abbreviations: AAPC, average annual percentage change; DALYs, disability-adjusted life-years; ^*^AAPC is statistically significant.

**Table S4. The age-standardized rate of incidence, mortality, and DALYs of urinary tract infections from 1990 to 2021 at the regional level.**

| Location | Year | Rate (95% UI) per 100 000 population | | |
| --- | --- | --- | --- | --- |
|  |  | Incidence | Mortality | DALYs |
| Central Asia | 1990 | 7401.8 (5975.66-9159.51) | 9.18 (8.06-10.59) | 163.65 (145.95-185.92) |
| Central Asia | 1991 | 7384.74 (5987.49-9136.08) | 9.57 (8.44-10.97) | 170.89 (152.95-192.95) |
| Central Asia | 1992 | 7375.66 (5969.54-9066.18) | 10.12 (8.95-11.52) | 180.32 (161.62-202.9) |
| Central Asia | 1993 | 7372.92 (5963.62-9064.63) | 10.82 (9.61-12.17) | 192.25 (173.17-214.23) |
| Central Asia | 1994 | 7374.27 (5940.38-9080.58) | 11.04 (9.8-12.33) | 194.82 (175.01-215.47) |
| Central Asia | 1995 | 7377.13 (5938.9-9138.53) | 10.92 (9.71-12.21) | 193.59 (174.24-214.49) |
| Central Asia | 1996 | 7395.92 (5994.22-9104.18) | 10.72 (9.52-11.95) | 189.42 (170.46-209.33) |
| Central Asia | 1997 | 7435.59 (6029.62-9099.03) | 10.19 (9.06-11.34) | 179.39 (161.58-198.23) |
| Central Asia | 1998 | 7483.65 (6107.71-9102.43) | 9.92 (8.77-11.11) | 173 (155.39-192.31) |
| Central Asia | 1999 | 7525.65 (6183.51-9115.05) | 10.18 (8.99-11.41) | 177.84 (158.95-198.36) |
| Central Asia | 2000 | 7553.38 (6221.05-9069.16) | 10.21 (9-11.47) | 178.51 (159.49-199.08) |
| Central Asia | 2001 | 7577.12 (6243.53-9100.88) | 10.52 (9.24-11.85) | 183.89 (163.6-205.72) |
| Central Asia | 2002 | 7610.35 (6277.52-9142.01) | 11.06 (9.71-12.43) | 192.5 (170.64-214.88) |
| Central Asia | 2003 | 7646.01 (6303.66-9256.01) | 11.72 (10.27-13.15) | 203.1 (179.51-226.22) |
| Central Asia | 2004 | 7679.17 (6324.81-9356.61) | 12.28 (10.75-13.81) | 213.05 (188.79-237.97) |
| Central Asia | 2005 | 7705.58 (6350.16-9453.56) | 14.36 (12.74-16.03) | 249.26 (223.69-275.93) |
| Central Asia | 2006 | 7716.27 (6383.38-9371.14) | 14.92 (13.15-16.8) | 255.89 (228.47-285.08) |
| Central Asia | 2007 | 7712.74 (6361.27-9288.35) | 15.08 (13.31-16.96) | 256.34 (228.74-285.18) |
| Central Asia | 2008 | 7703.64 (6358.05-9250.11) | 15.99 (14.15-17.88) | 269.43 (241.34-298.53) |
| Central Asia | 2009 | 7698.18 (6329.21-9226.41) | 17.64 (15.65-19.66) | 295.57 (264.91-326.48) |
| Central Asia | 2010 | 7704.76 (6275.07-9263.09) | 17.86 (15.85-19.76) | 299.15 (267.99-329.05) |
| Central Asia | 2011 | 7728.23 (6318.7-9264.97) | 19.01 (16.97-20.94) | 317.77 (286.13-348.45) |
| Central Asia | 2012 | 7764.38 (6386.56-9354.04) | 19.61 (17.45-21.64) | 321.13 (288.65-352.59) |
| Central Asia | 2013 | 7802.61 (6388.95-9423.78) | 24.6 (22-27.07) | 393.58 (355.65-430.89) |
| Central Asia | 2014 | 7836.09 (6356.53-9502.94) | 26.11 (23.32-28.74) | 415.42 (375.18-455.42) |
| Central Asia | 2015 | 7860.51 (6321.41-9616.81) | 26.39 (23.55-29.01) | 419.38 (378.2-459.61) |
| Central Asia | 2016 | 7891.35 (6401.03-9606.99) | 23.86 (21.04-26.47) | 379.83 (337.74-419.97) |
| Central Asia | 2017 | 7931.94 (6505.85-9610.46) | 22.23 (19.48-24.81) | 357.96 (316.82-398.48) |
| Central Asia | 2018 | 7968.7 (6555.69-9652.95) | 18.17 (15.72-20.54) | 297.49 (260.3-335.19) |
| Central Asia | 2019 | 7991.79 (6528.73-9714.66) | 16.8 (14.39-19.16) | 276.93 (239.89-314.3) |
| Central Asia | 2020 | 7917.74 (6478.12-9539.14) | 16.57 (14.1-19.03) | 274.31 (236.64-313.17) |
| Central Asia | 2021 | 8018.22 (6415.96-9887.25) | 16.49 (13.78-19.17) | 274.05 (231.78-317.2) |
| Central Europe | 1990 | 10111.92 (8239.97-12438.64) | 21.65 (19.83-23.23) | 352.34 (326-376.38) |
| Central Europe | 1991 | 10051.9 (8214.71-12290.35) | 19.19 (17.6-20.6) | 311.1 (287.81-332.84) |
| Central Europe | 1992 | 9995.79 (8171.48-12194.15) | 17.76 (16.27-19.05) | 286.33 (264.47-306.17) |
| Central Europe | 1993 | 9942.74 (8122.01-12133.9) | 16.55 (15.14-17.75) | 264.24 (243.7-282.26) |
| Central Europe | 1994 | 9895.73 (8075.18-12051.56) | 15.51 (14.17-16.64) | 246.05 (226.98-262.56) |
| Central Europe | 1995 | 9862.81 (8012.69-12054.56) | 14.07 (12.83-15.12) | 222.58 (204.79-238.02) |
| Central Europe | 1996 | 9837.58 (8036.35-11951.73) | 13.35 (12.15-14.37) | 210.95 (193.81-225.72) |
| Central Europe | 1997 | 9810.08 (8052.66-11883.62) | 12.93 (11.76-13.89) | 204.23 (187.26-218.53) |
| Central Europe | 1998 | 9778.88 (8042.3-11865.43) | 11.71 (10.62-12.62) | 185.17 (169.67-198.62) |
| Central Europe | 1999 | 9745.22 (7987.3-11844.01) | 11.13 (10.11-12.03) | 175.18 (160.63-188.67) |
| Central Europe | 2000 | 9714.23 (7933.02-11852) | 10.23 (9.3-11.11) | 162.26 (148.8-174.95) |
| Central Europe | 2001 | 9664.44 (7906.61-11720.31) | 9.53 (8.65-10.38) | 150.06 (137.3-162.87) |
| Central Europe | 2002 | 9586.09 (7848.75-11573.51) | 9.05 (8.21-9.91) | 141.78 (129.55-154.35) |
| Central Europe | 2003 | 9500.91 (7771.17-11509.01) | 8.75 (7.93-9.6) | 135.22 (123.38-147.93) |
| Central Europe | 2004 | 9432.11 (7699.53-11429.22) | 8.56 (7.71-9.41) | 130.38 (118.36-143.5) |
| Central Europe | 2005 | 9403.22 (7660.4-11436.17) | 8.76 (7.86-9.64) | 133.22 (120.68-146.44) |
| Central Europe | 2006 | 9448.86 (7717-11473.4) | 8.45 (7.55-9.33) | 128.88 (116.34-141.95) |
| Central Europe | 2007 | 9562.23 (7836.85-11586.72) | 7.89 (7.02-8.73) | 120.29 (108.26-132.86) |
| Central Europe | 2008 | 9703.02 (7981.1-11762.35) | 7.85 (6.98-8.69) | 119.43 (107.52-131.77) |
| Central Europe | 2009 | 9831.27 (8104.75-11894.84) | 8.21 (7.3-9.08) | 124.7 (112.46-137.38) |
| Central Europe | 2010 | 9907.19 (8138.9-11982.07) | 8.84 (7.83-9.73) | 132.55 (118.97-145.53) |
| Central Europe | 2011 | 9929.2 (8209.91-12017.12) | 9.45 (8.33-10.38) | 138.69 (124.24-152.02) |
| Central Europe | 2012 | 9933.5 (8224.79-12008.59) | 12.33 (10.88-13.42) | 177.23 (159.19-192.23) |
| Central Europe | 2013 | 9926.13 (8195.93-12011.18) | 14.68 (12.96-15.95) | 208.06 (186.73-225.01) |
| Central Europe | 2014 | 9920.49 (8168.87-12020.62) | 16.47 (14.51-17.99) | 230 (206.14-249.67) |
| Central Europe | 2015 | 9922.18 (8135.11-12045.78) | 18.6 (16.4-20.12) | 257.74 (231.37-277.53) |
| Central Europe | 2016 | 9926.98 (8121.98-12020.75) | 19.54 (17.2-21.37) | 268.19 (240.24-291.34) |
| Central Europe | 2017 | 9929.99 (8107-12034.72) | 20.95 (18.49-22.71) | 288.51 (258.99-310.91) |
| Central Europe | 2018 | 9935.63 (8057.66-12055.04) | 22.65 (20.02-24.49) | 309.81 (278.33-333.3) |
| Central Europe | 2019 | 9945.37 (8004.43-12117.03) | 23.16 (20.3-25.13) | 314.92 (281.3-339.68) |
| Central Europe | 2020 | 9986.21 (8118.29-12177.92) | 23.41 (20.28-26.05) | 322.18 (282.08-360.82) |
| Central Europe | 2021 | 10044.47 (8088.62-12403.82) | 23.74 (20.11-27.3) | 329.72 (282.5-382.3) |
| Eastern Europe | 1990 | 9106.29 (7342.38-11057.7) | 13.56 (12.7-14.26) | 254.95 (239.8-266.97) |
| Eastern Europe | 1991 | 9109.48 (7344.41-11058.57) | 13.68 (12.79-14.37) | 256.74 (241.34-268.89) |
| Eastern Europe | 1992 | 9112.46 (7347.56-11080.34) | 14.08 (13.15-14.8) | 261.57 (245.7-274.08) |
| Eastern Europe | 1993 | 9115.2 (7364.68-11074.79) | 14.15 (13.35-14.8) | 264.03 (250.33-275.6) |
| Eastern Europe | 1994 | 9115.72 (7369.44-11077.7) | 13.98 (13.2-14.59) | 261.71 (248.72-272.49) |
| Eastern Europe | 1995 | 9115.8 (7346.19-11089.53) | 13.64 (12.85-14.27) | 254.68 (241.47-266.04) |
| Eastern Europe | 1996 | 9116.86 (7373-11034.81) | 12.97 (12.21-13.59) | 241.25 (228.38-252.15) |
| Eastern Europe | 1997 | 9113.63 (7377.41-11000.33) | 12.41 (11.68-13) | 230.23 (218.51-240.22) |
| Eastern Europe | 1998 | 9112.03 (7383.01-10980.59) | 12.24 (11.5-12.84) | 225.1 (213.27-235.28) |
| Eastern Europe | 1999 | 9106.05 (7389.85-10998.36) | 12.8 (12.07-13.39) | 234.45 (222.65-244.29) |
| Eastern Europe | 2000 | 9101.46 (7375.09-11031.14) | 12.87 (12.13-13.48) | 234.32 (222.76-244.54) |
| Eastern Europe | 2001 | 9092.76 (7369.06-11002.17) | 12.41 (11.66-13.04) | 225.9 (213.73-236.59) |
| Eastern Europe | 2002 | 9077.41 (7336.75-10956.45) | 11.99 (11.27-12.58) | 217.54 (206.07-227.49) |
| Eastern Europe | 2003 | 9065.83 (7301.19-10946.08) | 11.43 (10.73-11.98) | 208 (196.73-217.52) |
| Eastern Europe | 2004 | 9062.68 (7302.58-10941.54) | 11.05 (10.34-11.65) | 201.42 (190.07-211.72) |
| Eastern Europe | 2005 | 9075 (7310.5-10991.85) | 11.58 (10.84-12.16) | 209.13 (197.64-219.11) |
| Eastern Europe | 2006 | 9112.33 (7353.3-10968.54) | 11.21 (10.47-11.81) | 201.88 (190.02-211.88) |
| Eastern Europe | 2007 | 9179.09 (7441.56-11023.12) | 11.2 (10.46-11.82) | 201.48 (189.51-212.08) |
| Eastern Europe | 2008 | 9252.4 (7556.09-11076.83) | 11.47 (10.71-12.09) | 203.3 (191.28-213.69) |
| Eastern Europe | 2009 | 9312.75 (7625.35-11153.22) | 11.73 (10.92-12.35) | 205.96 (193.43-216.38) |
| Eastern Europe | 2010 | 9349.87 (7667.86-11196.95) | 12.41 (11.55-13.08) | 217.26 (204.05-228.33) |
| Eastern Europe | 2011 | 9343.81 (7688.43-11102.36) | 12.15 (11.25-12.82) | 211.37 (197.6-222.21) |
| Eastern Europe | 2012 | 9318.08 (7660.68-11024.12) | 12.7 (11.71-13.41) | 217.17 (202.42-228.47) |
| Eastern Europe | 2013 | 9285.37 (7647.44-10951.57) | 14.24 (13.13-15.03) | 238.45 (222.14-251.3) |
| Eastern Europe | 2014 | 9259.33 (7645.54-10935.42) | 17.44 (16.06-18.39) | 283.4 (264.7-297.34) |
| Eastern Europe | 2015 | 9261.88 (7636.21-10949.45) | 21.54 (19.83-22.67) | 342.42 (319.74-358.62) |
| Eastern Europe | 2016 | 9306.2 (7665.25-10968.04) | 24.09 (22.12-25.39) | 377.89 (352.19-395.99) |
| Eastern Europe | 2017 | 9385.5 (7701.9-11038.61) | 26.24 (23.9-27.86) | 405.32 (374.86-428.18) |
| Eastern Europe | 2018 | 9470.09 (7763.3-11100.41) | 29.69 (26.94-31.66) | 452.93 (417.51-481.03) |
| Eastern Europe | 2019 | 9524.98 (7835.63-11167.3) | 31.64 (28.62-33.74) | 479.38 (440.33-508.69) |
| Eastern Europe | 2020 | 9514.64 (7822-11156.89) | 31.69 (28.14-34.59) | 487.06 (437.53-534.66) |
| Eastern Europe | 2021 | 9634.42 (7883.75-11337.72) | 32.95 (28.36-37.16) | 510.65 (443.02-577.45) |
| Australasia | 1990 | 11947.35 (8901.37-15405.77) | 18.3 (15.07-21.11) | 246.82 (206.13-283.65) |
| Australasia | 1991 | 11824.16 (8933.69-14999.14) | 16.49 (13.48-19.1) | 222.58 (185.24-256.45) |
| Australasia | 1992 | 11717.72 (8935.47-14793.33) | 15.17 (12.35-17.61) | 203 (168.41-234.22) |
| Australasia | 1993 | 11635.18 (8883.04-14640.83) | 15.17 (12.34-17.61) | 201.54 (166.52-232.46) |
| Australasia | 1994 | 11582.64 (8757.69-14712.3) | 15.62 (12.67-18.08) | 208.37 (172.04-240.03) |
| Australasia | 1995 | 11566.94 (8546.42-14821.44) | 15.21 (12.37-17.66) | 202.36 (167.63-233.47) |
| Australasia | 1996 | 11586.75 (8674.87-14783.38) | 15.65 (12.7-18.2) | 205.71 (170.02-238.29) |
| Australasia | 1997 | 11628.73 (8732.74-14855.16) | 19.27 (15.46-22.43) | 242.27 (198.59-280.8) |
| Australasia | 1998 | 11682.35 (8739.73-15025.48) | 20.83 (16.56-24.35) | 259.77 (210.2-302.41) |
| Australasia | 1999 | 11737.33 (8681.51-15301.94) | 21.12 (16.74-24.75) | 261.94 (212.02-305.29) |
| Australasia | 2000 | 11782.9 (8558.15-15666.21) | 21.01 (16.64-24.62) | 259.82 (209.87-302.75) |
| Australasia | 2001 | 11816.56 (8734.26-15463.34) | 22.55 (17.81-26.48) | 278.58 (224.9-325.68) |
| Australasia | 2002 | 11845.28 (8859.55-15382.78) | 22.27 (17.6-26.14) | 275.67 (222.49-321.94) |
| Australasia | 2003 | 11871.77 (8921.73-15394.75) | 22.57 (17.74-26.54) | 276.51 (221.57-322.98) |
| Australasia | 2004 | 11898.77 (8899.84-15528.38) | 22.81 (17.7-26.95) | 275.07 (218.4-322.96) |
| Australasia | 2005 | 11929.16 (8846.44-15691.44) | 22.86 (17.73-26.88) | 272.8 (216.43-319.36) |
| Australasia | 2006 | 11965.42 (8965.23-15499.59) | 22.99 (17.82-27.08) | 275.44 (217.95-322.35) |
| Australasia | 2007 | 12003.77 (9018.93-15383.58) | 20.14 (15.67-23.64) | 242.04 (192.62-282.44) |
| Australasia | 2008 | 12036.37 (8986.49-15217.3) | 17.06 (13.37-20.01) | 205.56 (164.9-239.33) |
| Australasia | 2009 | 12057.47 (8899.9-15338.06) | 16.06 (12.55-18.86) | 193.68 (154.96-226.28) |
| Australasia | 2010 | 12061.63 (8708.98-15558.28) | 19.05 (14.87-22.5) | 225.17 (179.22-264.52) |
| Australasia | 2011 | 12026.51 (8783.94-15455.94) | 22.55 (17.55-26.53) | 266.2 (212.01-311.54) |
| Australasia | 2012 | 11951.76 (8745.81-15341.1) | 25.1 (19.59-29.7) | 297.13 (237.13-349.58) |
| Australasia | 2013 | 11866.8 (8730.48-15353.75) | 25.69 (20.03-30.37) | 304 (242.04-356.81) |
| Australasia | 2014 | 11798.23 (8647.96-15393.81) | 26.28 (20.52-31.1) | 308.61 (246.66-362.97) |
| Australasia | 2015 | 11774.81 (8534.82-15611.21) | 26.1 (20.31-30.81) | 304.05 (241.5-356.41) |
| Australasia | 2016 | 11807.77 (8619.31-15406.51) | 26.17 (20.14-31.07) | 303.89 (238.51-358.05) |
| Australasia | 2017 | 11869.37 (8693.4-15526.35) | 27.25 (20.89-32.36) | 317.63 (248.9-374.58) |
| Australasia | 2018 | 11927.68 (8668.49-15651.14) | 26.82 (20.42-31.91) | 312.09 (243.02-369.36) |
| Australasia | 2019 | 11953.2 (8605.77-15795.62) | 27 (20.49-32.22) | 313.87 (243.85-371.65) |
| Australasia | 2020 | 11566.79 (8529.13-15533) | 25.18 (18.78-30.37) | 292.78 (224.08-350.27) |
| Australasia | 2021 | 12218.76 (8640.06-16316.47) | 26.04 (19.54-31.44) | 303.8 (234.1-364.6) |
| High-income Asia Pacific | 1990 | 7006.73 (5192.13-8996.99) | 11.48 (9.31-13.46) | 149.97 (125.41-176.24) |
| High-income Asia Pacific | 1991 | 7043.98 (5242.66-9021.14) | 10.77 (8.72-12.61) | 141.36 (118.08-166.02) |
| High-income Asia Pacific | 1992 | 7075.3 (5278.27-9059.27) | 9.97 (8.02-11.68) | 131.07 (108.61-153.93) |
| High-income Asia Pacific | 1993 | 7099.96 (5310.94-9106.17) | 9.44 (7.6-11.04) | 126.03 (104.77-147.24) |
| High-income Asia Pacific | 1994 | 7116.21 (5316.91-9145.3) | 10.24 (8.2-11.83) | 135 (111.85-155.74) |
| High-income Asia Pacific | 1995 | 7123.19 (5306.49-9176.49) | 10.78 (8.59-12.36) | 141.57 (116.94-161.51) |
| High-income Asia Pacific | 1996 | 7117.99 (5310.71-9151.45) | 10.24 (8.09-11.75) | 134.67 (110.66-153.65) |
| High-income Asia Pacific | 1997 | 7101.14 (5283-9142.1) | 9.64 (7.62-11.09) | 128.25 (105.3-146.38) |
| High-income Asia Pacific | 1998 | 7082.05 (5257.71-9119.64) | 9.61 (7.59-11.04) | 127.72 (105.06-145.87) |
| High-income Asia Pacific | 1999 | 7066.03 (5227.55-9122.79) | 9.69 (7.63-11.18) | 128.24 (105.26-147.02) |
| High-income Asia Pacific | 2000 | 7061.01 (5225.53-9147.18) | 9.47 (7.41-10.94) | 125.6 (102.29-144.29) |
| High-income Asia Pacific | 2001 | 7119.83 (5318.22-9130.27) | 9.39 (7.28-10.88) | 124.22 (100.43-142.83) |
| High-income Asia Pacific | 2002 | 7257.39 (5465.61-9191.37) | 9.41 (7.28-10.91) | 125.4 (101.19-143.97) |
| High-income Asia Pacific | 2003 | 7428.31 (5639.22-9338.55) | 9.39 (7.2-10.9) | 124.2 (99.67-142.64) |
| High-income Asia Pacific | 2004 | 7587.3 (5790.41-9492.02) | 9.94 (7.61-11.53) | 130.96 (104.93-150.07) |
| High-income Asia Pacific | 2005 | 7691.22 (5876.76-9633.77) | 10.51 (8.01-12.21) | 137 (109.21-157.07) |
| High-income Asia Pacific | 2006 | 7760.26 (5960.01-9669.61) | 10.82 (8.16-12.6) | 139.91 (110.42-160.91) |
| High-income Asia Pacific | 2007 | 7836.16 (6051.31-9735.33) | 11.24 (8.43-13.14) | 144.3 (113.19-166.38) |
| High-income Asia Pacific | 2008 | 7906.53 (6132.44-9810.93) | 11.82 (8.84-13.8) | 151.48 (118.71-174.51) |
| High-income Asia Pacific | 2009 | 7958.04 (6182.83-9844.73) | 12.56 (9.36-14.65) | 159.76 (124.83-183.55) |
| High-income Asia Pacific | 2010 | 7974.9 (6204.94-9860.87) | 13.45 (10-15.7) | 168.82 (131.6-194.33) |
| High-income Asia Pacific | 2011 | 7944.08 (6165.59-9812.12) | 14.65 (10.88-17.06) | 182.78 (142.02-209.77) |
| High-income Asia Pacific | 2012 | 7881.33 (6115.59-9741.36) | 15.07 (11.11-17.63) | 186.14 (143.47-214.41) |
| High-income Asia Pacific | 2013 | 7806.13 (6067.44-9671.11) | 15.53 (11.38-18.22) | 190.25 (145.84-220) |
| High-income Asia Pacific | 2014 | 7740.19 (5987.15-9612.82) | 16.12 (11.76-19.03) | 196.43 (149.73-228.36) |
| High-income Asia Pacific | 2015 | 7705.17 (5932.99-9601.59) | 16.37 (11.84-19.49) | 198.16 (149.85-232.34) |
| High-income Asia Pacific | 2016 | 7711.37 (5922.24-9630.08) | 16.9 (12.15-20.28) | 204.84 (153.85-241.96) |
| High-income Asia Pacific | 2017 | 7738.1 (5906.45-9702.54) | 15.97 (11.43-19.3) | 192.78 (144.15-229.66) |
| High-income Asia Pacific | 2018 | 7768.52 (5892.18-9787.97) | 16.12 (11.47-19.59) | 193.18 (143.51-231.75) |
| High-income Asia Pacific | 2019 | 7781.37 (5841.75-9864.53) | 16.46 (11.68-20.05) | 195.8 (144.99-235.89) |
| High-income Asia Pacific | 2020 | 7747.53 (5804.31-9843.54) | 15.94 (11.1-19.73) | 189.78 (138.48-231.84) |
| High-income Asia Pacific | 2021 | 7876.58 (5907.04-10084.82) | 16.32 (11.45-20.11) | 194.26 (142.43-236.88) |
| High-income North America | 1990 | 21039.96 (16777.06-26029.84) | 30.58 (25.36-33.46) | 407.23 (349.6-440.75) |
| High-income North America | 1991 | 19873.11 (15940.53-24518) | 30.04 (24.89-32.88) | 400.76 (343.33-433.61) |
| High-income North America | 1992 | 18895.48 (15243.55-23233.91) | 29.53 (24.44-32.39) | 392.63 (336.17-425.35) |
| High-income North America | 1993 | 18148.13 (14741.27-22282.47) | 30.19 (25.03-32.97) | 398.95 (341.91-431.01) |
| High-income North America | 1994 | 17672.67 (14404.28-21648.41) | 31.01 (25.69-33.87) | 407.31 (348.67-440.33) |
| High-income North America | 1995 | 17509.16 (14305-21453.46) | 31.4 (26.06-34.31) | 411.93 (353.37-445.52) |
| High-income North America | 1996 | 17662.66 (14387.66-21619.37) | 31.62 (26.22-34.55) | 414.3 (355.54-448.02) |
| High-income North America | 1997 | 18034.82 (14687.21-22067.8) | 32.54 (26.99-35.64) | 425.43 (365.04-461.02) |
| High-income North America | 1998 | 18512 (15044.23-22624.9) | 34.34 (28.45-37.64) | 448.63 (384.68-486.33) |
| High-income North America | 1999 | 18982.27 (15417.16-23146.12) | 36.98 (30.63-40.42) | 479.9 (410.73-519) |
| High-income North America | 2000 | 19333.7 (15699.34-23573.37) | 38.07 (31.55-41.66) | 493.61 (422.16-534.47) |
| High-income North America | 2001 | 19565.45 (15926.9-23764.25) | 39.25 (32.58-42.99) | 508.78 (435.39-551.03) |
| High-income North America | 2002 | 19768 (16143.08-23974.02) | 39.64 (32.78-43.41) | 513.25 (438.16-555.7) |
| High-income North America | 2003 | 19967.7 (16333.84-24147.42) | 39.89 (32.9-43.71) | 516.05 (439.49-559.34) |
| High-income North America | 2004 | 20190.82 (16537.55-24335.49) | 40.17 (33-44.2) | 518.4 (439.78-563.9) |
| High-income North America | 2005 | 20463.52 (16765.39-24611.27) | 41.52 (33.97-45.59) | 534.19 (451.67-579.99) |
| High-income North America | 2006 | 20962.94 (17317.83-24994.23) | 41.29 (33.71-45.51) | 531.52 (447.95-578.61) |
| High-income North America | 2007 | 21711.8 (18083.5-25720.32) | 39.82 (32.41-43.89) | 515.77 (434.4-561.49) |
| High-income North America | 2008 | 22505.29 (18868.21-26521.81) | 35.3 (28.7-38.81) | 462.89 (390.66-503.22) |
| High-income North America | 2009 | 23139.7 (19489.76-27174.98) | 34.26 (27.83-37.78) | 449.79 (378.87-490.86) |
| High-income North America | 2010 | 23410.53 (19782.66-27462.4) | 33.72 (27.33-37.17) | 444.43 (373.83-484.06) |
| High-income North America | 2011 | 23389.55 (19814.67-27405.31) | 34.07 (27.61-37.56) | 450.25 (379.41-490.8) |
| High-income North America | 2012 | 23311.59 (19759.11-27235.92) | 34.12 (27.57-37.66) | 451.5 (379.68-492.47) |
| High-income North America | 2013 | 23212.24 (19681.88-27072.45) | 34.11 (27.59-37.63) | 454.22 (382.1-495.1) |
| High-income North America | 2014 | 23126.23 (19606.06-26940.76) | 35.11 (28.42-38.77) | 468.98 (394.65-511.55) |
| High-income North America | 2015 | 23089.15 (19601.95-26926.23) | 35.22 (28.56-38.86) | 473.7 (398.5-516.54) |
| High-income North America | 2016 | 23169.29 (19546.35-27154.4) | 35.44 (28.77-39.09) | 478.91 (403.56-521.99) |
| High-income North America | 2017 | 23351.75 (19556.45-27592.44) | 34.87 (28.3-38.44) | 472.91 (397.94-515.05) |
| High-income North America | 2018 | 23543.18 (19476.4-28086.41) | 33.84 (27.38-37.39) | 460.86 (387.26-502.44) |
| High-income North America | 2019 | 23649.2 (19318.95-28497.3) | 33.15 (26.82-36.65) | 450.99 (378.21-491.91) |
| High-income North America | 2020 | 23417.82 (18946.82-28502.61) | 33.43 (27.04-37.54) | 455.12 (381.68-505.65) |
| High-income North America | 2021 | 23205.5 (18537.34-28619.47) | 33.65 (27.23-37.67) | 459.11 (385.4-508.44) |
| Southern Latin America | 1990 | 10888.24 (8416.65-13807.91) | 19.13 (16.29-21.7) | 263.67 (227.21-297.99) |
| Southern Latin America | 1991 | 11038.81 (8682.15-13798.47) | 22.73 (19.26-25.86) | 310.91 (266.57-352.32) |
| Southern Latin America | 1992 | 11190.7 (8928.62-13813.77) | 23.09 (19.58-26.25) | 315.95 (271.83-357.27) |
| Southern Latin America | 1993 | 11339.44 (9124.36-13994.48) | 22.55 (19.24-25.59) | 310.62 (268.37-350.29) |
| Southern Latin America | 1994 | 11480.8 (9318.59-14185.39) | 22.12 (18.72-25.25) | 302.58 (259.81-342.43) |
| Southern Latin America | 1995 | 11610.58 (9393.92-14332.4) | 22.2 (18.89-25.19) | 301.89 (260.55-340.05) |
| Southern Latin America | 1996 | 11763.1 (9638.21-14312.93) | 24.44 (20.68-27.82) | 326.11 (280.37-368.81) |
| Southern Latin America | 1997 | 11957.51 (9942.8-14481.58) | 33.41 (28.27-37.93) | 444.25 (381.94-502.18) |
| Southern Latin America | 1998 | 12164.54 (10095.14-14778.66) | 38.54 (32.69-43.69) | 510.4 (439.59-575.66) |
| Southern Latin America | 1999 | 12357.83 (10210.52-15159.16) | 42.9 (36.34-48.7) | 566.45 (486.72-639.9) |
| Southern Latin America | 2000 | 12510.52 (10268.46-15481.3) | 48.45 (40.69-54.99) | 634.31 (541.53-716.85) |
| Southern Latin America | 2001 | 12635.22 (10402.09-15451.61) | 50.56 (42.48-57.42) | 663.41 (566.08-750.42) |
| Southern Latin America | 2002 | 12760.89 (10490.17-15461.83) | 52.37 (44-59.5) | 686.92 (586.64-776.59) |
| Southern Latin America | 2003 | 12883.61 (10553.62-15526.43) | 56.35 (47.39-64.06) | 737.79 (630.14-835.21) |
| Southern Latin America | 2004 | 12997.58 (10575.5-15761.58) | 59.4 (49.89-67.46) | 776.19 (661.83-878.18) |
| Southern Latin America | 2005 | 13097.17 (10506.63-16188.3) | 61.01 (51.2-69.32) | 795.62 (678.13-899.7) |
| Southern Latin America | 2006 | 13192.38 (10662.05-16167.49) | 61.99 (52.2-70.59) | 805.91 (688.58-913.43) |
| Southern Latin America | 2007 | 13293.6 (10705-16256.78) | 63.11 (53.04-72.02) | 816.35 (697.18-928.69) |
| Southern Latin America | 2008 | 13395.88 (10725.55-16469.56) | 61.24 (51.28-70.06) | 792.32 (673.86-903.29) |
| Southern Latin America | 2009 | 13495.43 (10730.95-16845.15) | 64.41 (53.68-73.57) | 831.6 (704.3-945.97) |
| Southern Latin America | 2010 | 13590.36 (10680.89-17243.16) | 70.25 (58.85-80.15) | 908.57 (773.3-1032.7) |
| Southern Latin America | 2011 | 13701.86 (10874.99-17211.6) | 72.53 (60.95-82.36) | 935.86 (799.37-1057.56) |
| Southern Latin America | 2012 | 13831.74 (11016.95-17197.6) | 73.96 (61.87-84.19) | 954.13 (811.19-1081.27) |
| Southern Latin America | 2013 | 13951.38 (11128.36-17276.59) | 76.1 (63.66-86.45) | 977.86 (831.23-1107.62) |
| Southern Latin America | 2014 | 14031.4 (11140.81-17224.24) | 80.19 (66.95-91.17) | 1030.93 (873.16-1167.55) |
| Southern Latin America | 2015 | 14042.79 (11099.46-17151.47) | 84.46 (70.7-96.43) | 1082.8 (918.09-1232.28) |
| Southern Latin America | 2016 | 13978.6 (11295.86-17007.2) | 90.75 (76.11-103.74) | 1158.93 (983.7-1320.52) |
| Southern Latin America | 2017 | 13872.56 (11357.39-16920.36) | 93.1 (77.86-106.31) | 1187.84 (1006.53-1352.59) |
| Southern Latin America | 2018 | 13753.59 (11321.64-16809.13) | 94.04 (78.46-107.42) | 1202.35 (1017.11-1368.98) |
| Southern Latin America | 2019 | 13650.8 (11174.65-16835.54) | 94.2 (78.35-107.95) | 1208.1 (1017.2-1383.89) |
| Southern Latin America | 2020 | 13729.38 (10929.49-16944.24) | 89.2 (73.66-102.87) | 1151 (964.47-1324.75) |
| Southern Latin America | 2021 | 13724.86 (10886.72-16749.46) | 85.74 (70.4-99.2) | 1105.39 (923.56-1272.86) |
| Western Europe | 1990 | 6400.15 (5226.42-7753.56) | 13.9 (11.93-15.12) | 183.78 (161.17-198.48) |
| Western Europe | 1991 | 6277.48 (5130.43-7604.45) | 13.25 (11.34-14.44) | 173.16 (151.55-187.4) |
| Western Europe | 1992 | 6168.44 (5047.99-7492.46) | 13.3 (11.36-14.5) | 171.61 (149.87-185.64) |
| Western Europe | 1993 | 6082.87 (4939.36-7412.01) | 14.66 (12.48-15.94) | 187.62 (163.77-202.22) |
| Western Europe | 1994 | 6027.85 (4881.64-7351.75) | 15.62 (13.3-17.01) | 198.96 (173.71-214.75) |
| Western Europe | 1995 | 6013.71 (4833.93-7325.28) | 16.83 (14.31-18.29) | 215.2 (187.54-231.93) |
| Western Europe | 1996 | 6018.86 (4851.27-7326.5) | 17.31 (14.7-18.83) | 220.66 (192.16-238.14) |
| Western Europe | 1997 | 6020.31 (4849.66-7313.91) | 18.07 (15.29-19.66) | 228.92 (198.67-247.33) |
| Western Europe | 1998 | 6021.68 (4835.42-7317.35) | 19.15 (16.2-20.88) | 241.9 (209.6-261.75) |
| Western Europe | 1999 | 6029.49 (4826.65-7328.06) | 20.19 (17.1-22.07) | 254.7 (220.96-276.62) |
| Western Europe | 2000 | 6046.5 (4843.98-7369.76) | 20.96 (17.71-22.9) | 263.69 (228.2-286.35) |
| Western Europe | 2001 | 6119.58 (4897.66-7457.1) | 22.17 (18.67-24.29) | 277.26 (239.14-301.71) |
| Western Europe | 2002 | 6261.57 (4982.31-7662.89) | 23.93 (20.21-26.14) | 297.71 (257.31-323.17) |
| Western Europe | 2003 | 6426.17 (5076.11-7950.28) | 25.63 (21.59-28.04) | 317.82 (273.89-345.27) |
| Western Europe | 2004 | 6566.45 (5136.37-8263.52) | 25.28 (21.22-27.8) | 313.15 (269.13-341.56) |
| Western Europe | 2005 | 6635.99 (5151.35-8445.02) | 26.09 (21.77-28.64) | 322.18 (275.64-350.82) |
| Western Europe | 2006 | 6650.18 (5172.78-8439.81) | 27.37 (22.73-30.09) | 336.62 (286.87-367.12) |
| Western Europe | 2007 | 6647.54 (5160.74-8413.89) | 27.96 (23.14-30.78) | 342.53 (290.69-374.21) |
| Western Europe | 2008 | 6639.72 (5145.54-8409.13) | 29.01 (23.92-31.99) | 353.62 (299.15-386.74) |
| Western Europe | 2009 | 6628.67 (5120.07-8412.5) | 29.24 (23.93-32.41) | 355.33 (298.55-390.36) |
| Western Europe | 2010 | 6609.77 (5083.01-8398.9) | 29.21 (23.83-32.37) | 354.24 (296.93-389.29) |
| Western Europe | 2011 | 6595.29 (5130.39-8375.14) | 27.64 (22.42-30.77) | 334.37 (278.77-369.17) |
| Western Europe | 2012 | 6569.22 (5150.91-8343.7) | 27.34 (22.23-30.34) | 329.86 (275.47-363.1) |
| Western Europe | 2013 | 6539.97 (5155.23-8299) | 27.56 (22.34-30.61) | 332.83 (277.09-366.61) |
| Western Europe | 2014 | 6511.9 (5139.73-8254.34) | 28.71 (23.08-32) | 345.46 (285.7-381.77) |
| Western Europe | 2015 | 6484.96 (5131.27-8247.04) | 29.64 (23.94-32.96) | 355.85 (294.94-392.45) |
| Western Europe | 2016 | 6401.43 (5114.63-8016.1) | 30.02 (24.05-33.45) | 359.12 (295.41-396.66) |
| Western Europe | 2017 | 6253.05 (5038.04-7701.64) | 29.88 (23.8-33.35) | 356.61 (291.85-394.76) |
| Western Europe | 2018 | 6102.09 (4937.85-7448.59) | 29.71 (23.66-33.24) | 354.44 (290.05-393.07) |
| Western Europe | 2019 | 6012.84 (4844.18-7339.67) | 29.23 (23.18-32.93) | 349.61 (285.32-390.15) |
| Western Europe | 2020 | 5965.89 (4763.7-7282.31) | 29.22 (23.07-33.23) | 349.09 (282.96-395.11) |
| Western Europe | 2021 | 5971.6 (4732.09-7220.42) | 28.19 (22.08-32.26) | 339.33 (273.26-386.07) |
| Andean Latin America | 1990 | 13419.36 (10625.66-16527.68) | 27.5 (19.98-40.93) | 366.01 (267.73-537.65) |
| Andean Latin America | 1991 | 13434.72 (10819.47-16387.94) | 26.43 (19.21-40.14) | 356.71 (261.65-529.39) |
| Andean Latin America | 1992 | 13461.8 (10894.37-16415.73) | 28.01 (20.48-40.7) | 377.44 (279.03-542.53) |
| Andean Latin America | 1993 | 13493.28 (10942.82-16420.08) | 28.21 (20.49-40.29) | 378.32 (277.77-534.27) |
| Andean Latin America | 1994 | 13529.9 (10971.03-16492.84) | 28.94 (21.2-41.06) | 388.54 (287.88-545.19) |
| Andean Latin America | 1995 | 13570.57 (10891.02-16581.95) | 30.08 (22.34-41.46) | 401.74 (300.78-550.12) |
| Andean Latin America | 1996 | 13642.64 (11207.42-16527.33) | 29.06 (21.71-39.66) | 386.63 (292.1-523.71) |
| Andean Latin America | 1997 | 13760.54 (11448.96-16554.76) | 31.18 (23.46-41.39) | 414.06 (315.48-545.8) |
| Andean Latin America | 1998 | 13897.4 (11610.2-16678.26) | 32.84 (24.62-43.15) | 434.87 (328.47-569.29) |
| Andean Latin America | 1999 | 14023.21 (11716.67-16801.2) | 34.58 (25.52-45.16) | 457.52 (341.96-594.35) |
| Andean Latin America | 2000 | 14110.17 (11667.19-16972.59) | 36.29 (26.15-47.72) | 480.26 (350.54-628.11) |
| Andean Latin America | 2001 | 14172.22 (11822.12-17013.47) | 37.64 (26.42-50.11) | 497.26 (353.37-656.42) |
| Andean Latin America | 2002 | 14240.01 (11896.12-17066.44) | 40.63 (28.06-54.3) | 535.42 (371.49-712.38) |
| Andean Latin America | 2003 | 14301.44 (11925.19-17220.08) | 41.94 (28.25-56.9) | 553.5 (374.27-747.56) |
| Andean Latin America | 2004 | 14369.41 (11922.89-17383.81) | 41.17 (27.33-55.52) | 543.61 (362.75-730.92) |
| Andean Latin America | 2005 | 14448.48 (11883.25-17647.29) | 39.51 (26.48-53.12) | 523.38 (351.52-701.22) |
| Andean Latin America | 2006 | 14618.17 (12098.44-17634.57) | 37.39 (25.4-50.62) | 497.64 (338.63-669.65) |
| Andean Latin America | 2007 | 14896.78 (12367.15-17838.28) | 36.13 (25.05-48.2) | 480.68 (334.27-638.36) |
| Andean Latin America | 2008 | 15193.65 (12489.19-18143.47) | 36.54 (25.19-48.94) | 488.27 (337.78-648.75) |
| Andean Latin America | 2009 | 15438.23 (12625.16-18454.2) | 37.41 (25.61-49.81) | 502.86 (343.95-667.95) |
| Andean Latin America | 2010 | 15549.41 (12525.2-18714.58) | 39.54 (26.79-52.22) | 531.96 (359.91-701.5) |
| Andean Latin America | 2011 | 15551.6 (12667.89-18580.91) | 39.39 (26.91-52.04) | 529.87 (360.61-700.17) |
| Andean Latin America | 2012 | 15532.5 (12679.29-18553.62) | 38.48 (26.58-50.91) | 517.14 (355.78-682.23) |
| Andean Latin America | 2013 | 15484.54 (12586.71-18642.13) | 37.96 (26.74-49.87) | 509.44 (356.69-667.74) |
| Andean Latin America | 2014 | 15428.35 (12455.18-18759.98) | 37.55 (26.44-49.48) | 503.3 (352.54-661.96) |
| Andean Latin America | 2015 | 15362.07 (12295.07-18880.69) | 38.81 (27.25-50.82) | 519.4 (363.39-678.97) |
| Andean Latin America | 2016 | 15280.22 (12278.35-18663.81) | 40.93 (28.4-53.8) | 547.09 (378.02-715.75) |
| Andean Latin America | 2017 | 15168.39 (12156.78-18570.05) | 44.9 (30.08-59.85) | 599.76 (399.85-793.78) |
| Andean Latin America | 2018 | 15075.18 (12038.72-18620.98) | 46.92 (31.16-62.96) | 625.38 (412.2-835.69) |
| Andean Latin America | 2019 | 15043.34 (11815.27-18923.49) | 49.43 (32.54-68.36) | 655.59 (430.16-899.66) |
| Andean Latin America | 2020 | 15268.08 (11967.74-18931.66) | 45.78 (30.01-64.32) | 618.41 (404.17-863.27) |
| Andean Latin America | 2021 | 15505.67 (12114.84-19228.46) | 44.33 (28.7-62.82) | 602.67 (389.16-850.57) |
| Caribbean | 1990 | 8194.66 (6462.57-10249.8) | 9.01 (7.68-11.07) | 126.73 (108.01-158.07) |
| Caribbean | 1991 | 8195.69 (6518.56-10163.43) | 8.5 (7.19-10.48) | 119.7 (101.46-149.81) |
| Caribbean | 1992 | 8199.44 (6573.83-10100.29) | 8.22 (6.94-10.14) | 115.88 (97.84-145.53) |
| Caribbean | 1993 | 8206.21 (6584.91-10077.39) | 9.06 (7.71-10.97) | 126.78 (107.96-156.29) |
| Caribbean | 1994 | 8216.41 (6609.91-10078.14) | 11.63 (9.94-13.84) | 159.7 (137.01-192.94) |
| Caribbean | 1995 | 8229.48 (6610.54-10138.12) | 13.67 (11.71-16.03) | 185.82 (160.04-219.74) |
| Caribbean | 1996 | 8249.35 (6664.99-10112.42) | 14.86 (12.73-17.28) | 201.37 (174.12-236.53) |
| Caribbean | 1997 | 8276.9 (6721.99-10156.79) | 16.14 (13.86-18.73) | 219.61 (190.51-257) |
| Caribbean | 1998 | 8307.6 (6753.91-10172.81) | 16.93 (14.62-19.54) | 233.94 (204.13-271.4) |
| Caribbean | 1999 | 8335.83 (6748.92-10201.8) | 16.47 (14.22-19.01) | 228.13 (198.96-265.16) |
| Caribbean | 2000 | 8357.42 (6737.3-10258.59) | 16.92 (14.61-19.58) | 234.03 (203.9-272.74) |
| Caribbean | 2001 | 8379.28 (6795.87-10268.53) | 17.49 (15.14-20.15) | 241.3 (210.76-280.09) |
| Caribbean | 2002 | 8409.28 (6834.91-10273.43) | 17.72 (15.29-20.53) | 244.02 (212.29-284.17) |
| Caribbean | 2003 | 8440.03 (6857.84-10303.42) | 18.01 (15.57-20.85) | 247.66 (215.68-288.46) |
| Caribbean | 2004 | 8468.04 (6881.39-10360.48) | 18.17 (15.66-20.92) | 250.45 (217.62-290.41) |
| Caribbean | 2005 | 8487.08 (6847.98-10401.02) | 17.9 (15.36-20.64) | 244.88 (211.37-284.15) |
| Caribbean | 2006 | 8501.05 (6895.86-10401.88) | 18.85 (16.15-21.79) | 257.69 (222.26-299.42) |
| Caribbean | 2007 | 8514.41 (6925.63-10400.89) | 18.86 (16.12-21.73) | 258.37 (222.69-298.95) |
| Caribbean | 2008 | 8527.16 (6925.72-10430.52) | 18.59 (15.82-21.42) | 255.77 (219.69-296.03) |
| Caribbean | 2009 | 8537 (6923.56-10486.33) | 18.52 (15.78-21.43) | 255.29 (219.71-297.18) |
| Caribbean | 2010 | 8545.04 (6918.66-10521.17) | 19.09 (16.21-22.13) | 262.28 (225.37-306.16) |
| Caribbean | 2011 | 8544.25 (6934.96-10472.95) | 19.39 (16.49-22.38) | 265.55 (228.12-308.07) |
| Caribbean | 2012 | 8531.66 (6902.24-10383.32) | 20.06 (17.02-23.16) | 275.62 (237.06-319.96) |
| Caribbean | 2013 | 8514.24 (6847.34-10331.18) | 21.15 (18.04-24.26) | 291.1 (251.68-335.81) |
| Caribbean | 2014 | 8494.48 (6815.15-10380.34) | 21.62 (18.49-24.76) | 297.75 (258.23-343.15) |
| Caribbean | 2015 | 8475.5 (6764.57-10424.93) | 21.72 (18.61-24.83) | 300.68 (261.2-345.46) |
| Caribbean | 2016 | 8438.19 (6798.74-10311.39) | 21.82 (18.62-24.89) | 303.38 (262.74-348.18) |
| Caribbean | 2017 | 8379.44 (6733.87-10264.21) | 22.35 (19.07-25.51) | 309.7 (267.91-354.57) |
| Caribbean | 2018 | 8322.12 (6661.41-10270.84) | 21.53 (18.31-24.68) | 297.18 (256.7-342.07) |
| Caribbean | 2019 | 8288.76 (6588.83-10258.49) | 21.85 (18.53-25.2) | 300.36 (258.16-347.21) |
| Caribbean | 2020 | 8267.9 (6658.43-10348.44) | 21.73 (18.25-25.51) | 298.07 (254.2-349.56) |
| Caribbean | 2021 | 8322.11 (6676.06-10290.45) | 22.15 (18.12-26.4) | 303.01 (251.42-360.36) |
| Central Latin America | 1990 | 9446.44 (7690.42-11587.24) | 24.37 (22.02-26.96) | 341.05 (312.42-373.3) |
| Central Latin America | 1991 | 9254.75 (7550.97-11283.56) | 23.43 (21.21-25.59) | 326.86 (299.52-354.31) |
| Central Latin America | 1992 | 9082.75 (7424.89-11016.44) | 22.66 (20.48-24.71) | 318.04 (291.1-344.43) |
| Central Latin America | 1993 | 8939.14 (7313.87-10818.45) | 21.47 (19.44-23.23) | 303.58 (278.41-326.33) |
| Central Latin America | 1994 | 8831.96 (7226.14-10678.45) | 21.15 (19.17-22.77) | 298.17 (273.68-319.65) |
| Central Latin America | 1995 | 8770.56 (7166.91-10601.67) | 21.26 (19.37-22.89) | 300.52 (277.07-321.73) |
| Central Latin America | 1996 | 8736.04 (7195.48-10545.37) | 21.16 (19.13-22.89) | 296.76 (272.13-318.85) |
| Central Latin America | 1997 | 8706.78 (7193.94-10505.08) | 21.18 (19.08-22.97) | 299.71 (273.99-322.84) |
| Central Latin America | 1998 | 8686.19 (7220.31-10467.71) | 20.8 (18.76-22.55) | 297.69 (272.55-320.74) |
| Central Latin America | 1999 | 8677.14 (7237.44-10440.1) | 19.78 (17.8-21.37) | 284.78 (260.19-306.22) |
| Central Latin America | 2000 | 8683.99 (7248.06-10447.54) | 20.15 (18.1-21.84) | 288.89 (263.27-311.8) |
| Central Latin America | 2001 | 8710.98 (7283.46-10453.49) | 20.58 (18.45-22.33) | 296.7 (270-320.01) |
| Central Latin America | 2002 | 8756.9 (7321.7-10490.42) | 21.83 (19.52-23.71) | 313.61 (284.54-338.16) |
| Central Latin America | 2003 | 8816.3 (7335.09-10538.17) | 22.52 (20.1-24.46) | 324.47 (294.04-350.15) |
| Central Latin America | 2004 | 8882.96 (7348.78-10591.81) | 22.82 (20.24-24.88) | 327.4 (295.17-354.49) |
| Central Latin America | 2005 | 8952.51 (7394.12-10664.6) | 24.6 (21.75-26.81) | 350.92 (315.84-379.73) |
| Central Latin America | 2006 | 9066.04 (7511.59-10803.14) | 25.87 (22.85-28.23) | 369.68 (332.48-400) |
| Central Latin America | 2007 | 9245.46 (7659.15-11029.52) | 26.64 (23.49-29.15) | 381.71 (342.46-414.26) |
| Central Latin America | 2008 | 9455.41 (7847.97-11304.72) | 28.01 (24.71-30.51) | 402.71 (362.86-434.94) |
| Central Latin America | 2009 | 9659.98 (8029.71-11588.09) | 28.76 (25.39-31.34) | 412.53 (371.44-445.39) |
| Central Latin America | 2010 | 9823.41 (8171.82-11814.04) | 29.88 (26.48-32.42) | 429.72 (388.71-462.76) |
| Central Latin America | 2011 | 9935.76 (8272.59-11948.24) | 31.67 (28.12-34.2) | 457.75 (414.78-491.49) |
| Central Latin America | 2012 | 10022.72 (8360.67-12021.85) | 33.33 (29.6-35.99) | 485.17 (439.44-520.59) |
| Central Latin America | 2013 | 10101.97 (8428.26-12096.15) | 36.15 (32.25-38.88) | 527.71 (480.01-564.02) |
| Central Latin America | 2014 | 10189.57 (8477.96-12184.24) | 39.53 (35.33-42.45) | 579.08 (527.35-617.96) |
| Central Latin America | 2015 | 10305.36 (8561.76-12346.43) | 42.04 (37.55-45.06) | 614.17 (558.71-654.19) |
| Central Latin America | 2016 | 10507.1 (8738.71-12572.27) | 43.55 (38.91-46.7) | 638.26 (580.82-680.11) |
| Central Latin America | 2017 | 10778.61 (9003.25-12884.04) | 43.63 (38.99-46.74) | 642.47 (584.72-684.35) |
| Central Latin America | 2018 | 11027.21 (9191.37-13225.67) | 44.97 (40.34-48.22) | 665.58 (606.75-709.58) |
| Central Latin America | 2019 | 11160.11 (9268.77-13461.95) | 46.54 (41.83-49.88) | 686.5 (627.2-731.4) |
| Central Latin America | 2020 | 11133.97 (9280.42-13294.64) | 45.85 (39.66-51.04) | 687.56 (601.59-765.67) |
| Central Latin America | 2021 | 11323.61 (9268.45-13731.21) | 47.02 (40.09-54.07) | 705.27 (605.22-816.3) |
| Tropical Latin America | 1990 | 15015.69 (11821.5-18709.74) | 28.32 (24.61-31.11) | 404.48 (358.45-441.55) |
| Tropical Latin America | 1991 | 15014.26 (11839.53-18676.76) | 28.29 (24.4-31.35) | 401.51 (354.06-441.78) |
| Tropical Latin America | 1992 | 15018.93 (11882.09-18599.22) | 29.32 (25.36-32.48) | 415.39 (367.08-456.18) |
| Tropical Latin America | 1993 | 15027.62 (11883.41-18621.06) | 30.18 (26.12-33.17) | 430.2 (380.72-469.35) |
| Tropical Latin America | 1994 | 15039.9 (11918.89-18594.88) | 29.86 (25.75-32.9) | 423.51 (373.92-463.42) |
| Tropical Latin America | 1995 | 15055.05 (11958.39-18595.46) | 29.93 (25.7-33.06) | 423.55 (372.24-463.62) |
| Tropical Latin America | 1996 | 15082.76 (12088.85-18477.04) | 29.88 (25.55-32.88) | 426.55 (373.66-465.45) |
| Tropical Latin America | 1997 | 15126.7 (12224.29-18424.6) | 30.14 (25.59-33.31) | 428.27 (373.05-468.34) |
| Tropical Latin America | 1998 | 15182.28 (12369.39-18378.06) | 31.29 (26.45-34.49) | 442.22 (383.73-483.22) |
| Tropical Latin America | 1999 | 15243.49 (12504.08-18342.05) | 32.37 (27.3-35.82) | 453.78 (392.98-496.92) |
| Tropical Latin America | 2000 | 15304.33 (12638.56-18393.74) | 36.32 (30.61-40.2) | 506.4 (437.9-554.63) |
| Tropical Latin America | 2001 | 15390.79 (12735.26-18406.41) | 40.41 (34.05-44.75) | 558.66 (482.73-611.79) |
| Tropical Latin America | 2002 | 15517.53 (12883.27-18520.81) | 43.15 (36.32-47.77) | 593.76 (512.56-650.38) |
| Tropical Latin America | 2003 | 15658.96 (13003.18-18672.61) | 45.82 (38.54-50.76) | 630.06 (543.73-691.31) |
| Tropical Latin America | 2004 | 15790.19 (13107.56-18841.25) | 47.31 (39.59-52.5) | 649.13 (557.87-713.34) |
| Tropical Latin America | 2005 | 15886.93 (13160.71-18952.98) | 48.23 (40.29-53.59) | 661.22 (567.95-727.66) |
| Tropical Latin America | 2006 | 15961.46 (13260.18-19033.76) | 45.89 (38.18-50.99) | 622.82 (533.09-685.61) |
| Tropical Latin America | 2007 | 16040.95 (13369.11-19102.03) | 46.96 (39.02-52.17) | 634.1 (541.61-697.95) |
| Tropical Latin America | 2008 | 16125.46 (13490.34-19194.84) | 49.84 (41.31-55.48) | 673.84 (575-742.92) |
| Tropical Latin America | 2009 | 16213.34 (13550.62-19332.97) | 56.63 (46.88-62.98) | 761.81 (648.25-839.94) |
| Tropical Latin America | 2010 | 16302.21 (13605.23-19514.58) | 64.9 (53.9-72.29) | 868.35 (739.71-957.83) |
| Tropical Latin America | 2011 | 16420.69 (13738.71-19590.41) | 70.9 (59.05-78.79) | 942.57 (805.63-1037.63) |
| Tropical Latin America | 2012 | 16587.82 (13856.14-19757.22) | 76.14 (63.36-84.79) | 1009.33 (861.65-1113.51) |
| Tropical Latin America | 2013 | 16771.84 (14023.86-19939.94) | 79.97 (66.67-89.11) | 1056 (900.91-1165.17) |
| Tropical Latin America | 2014 | 16941.12 (14149.06-20186.39) | 87.85 (72.94-97.86) | 1153.48 (981.25-1272.74) |
| Tropical Latin America | 2015 | 17064.08 (14228.28-20405.89) | 95.9 (79.74-107.04) | 1257.8 (1070.79-1390.31) |
| Tropical Latin America | 2016 | 17149.48 (14195.59-20526.57) | 102.41 (85.15-114.32) | 1342.63 (1145.27-1483.32) |
| Tropical Latin America | 2017 | 17220.44 (14233.28-20665.85) | 103.6 (85.44-115.94) | 1351.33 (1143.53-1496.69) |
| Tropical Latin America | 2018 | 17267.93 (14229.78-20833) | 106.6 (87.47-119.27) | 1388.41 (1169.4-1537.44) |
| Tropical Latin America | 2019 | 17283.21 (14229.24-21005.59) | 113.44 (92.74-126.97) | 1475.13 (1240.1-1634.42) |
| Tropical Latin America | 2020 | 17164.93 (14025.93-20798.29) | 113.61 (92.22-128.08) | 1487.16 (1243.13-1658.82) |
| Tropical Latin America | 2021 | 17297.78 (14150.66-20866.7) | 110.51 (88.9-125.1) | 1455.58 (1207.28-1629.42) |
| North Africa and Middle East | 1990 | 3250.34 (2623.45-4014.56) | 17.6 (12.67-34.2) | 242.95 (176.91-469.47) |
| North Africa and Middle East | 1991 | 3260.75 (2644.57-4004.11) | 17.21 (12.41-32.49) | 240.22 (174.94-449.94) |
| North Africa and Middle East | 1992 | 3271.41 (2649.84-4005.57) | 17.21 (12.43-32.08) | 241.6 (176.17-444.59) |
| North Africa and Middle East | 1993 | 3281.91 (2663.39-4025.12) | 17.26 (12.49-31.75) | 243.13 (177.94-441.16) |
| North Africa and Middle East | 1994 | 3291.78 (2668.82-4036.66) | 17.27 (12.56-31.44) | 244.67 (179.93-438.99) |
| North Africa and Middle East | 1995 | 3300.49 (2660.57-4037.95) | 17.44 (12.69-31.44) | 247.84 (182.26-440.36) |
| North Africa and Middle East | 1996 | 3309.98 (2671.16-4058.34) | 17.63 (12.84-31.5) | 250.89 (185.16-441.87) |
| North Africa and Middle East | 1997 | 3321.55 (2687.98-4068.82) | 17.64 (12.88-31.37) | 251.91 (185.77-441.27) |
| North Africa and Middle East | 1998 | 3334.13 (2707.68-4069.1) | 17.53 (12.82-30.45) | 252.23 (186.51-431.5) |
| North Africa and Middle East | 1999 | 3346.28 (2723.65-4105.74) | 17.59 (12.87-29.86) | 253.53 (187.22-423.67) |
| North Africa and Middle East | 2000 | 3356.65 (2725.16-4124.91) | 17.61 (12.97-29.43) | 251.9 (187.35-415.29) |
| North Africa and Middle East | 2001 | 3388.63 (2757.68-4148.99) | 17.45 (12.89-28.5) | 249.45 (186.39-403.11) |
| North Africa and Middle East | 2002 | 3453.39 (2798.96-4236.9) | 17.27 (12.84-27.46) | 246.55 (185.45-388.12) |
| North Africa and Middle East | 2003 | 3530.39 (2842.1-4345.66) | 17.21 (12.8-27.27) | 244.42 (183.79-383.2) |
| North Africa and Middle East | 2004 | 3598.24 (2876.28-4444.08) | 17.19 (12.86-27.15) | 243.57 (184.23-379.96) |
| North Africa and Middle East | 2005 | 3634.59 (2895.6-4528.6) | 17.21 (12.92-26.99) | 243.79 (184.97-376.69) |
| North Africa and Middle East | 2006 | 3649.37 (2913.35-4556.64) | 17.3 (12.97-26.66) | 244.28 (185.36-371.66) |
| North Africa and Middle East | 2007 | 3664.36 (2928.38-4576.26) | 17.46 (13.1-26.22) | 245.69 (186.79-364.96) |
| North Africa and Middle East | 2008 | 3677.63 (2916.26-4597.6) | 17.81 (13.39-25.97) | 249.7 (190.43-361.39) |
| North Africa and Middle East | 2009 | 3686.89 (2905.11-4617.44) | 18.31 (13.85-26.4) | 255.29 (195.91-363.76) |
| North Africa and Middle East | 2010 | 3690.97 (2902.32-4634.35) | 18.42 (13.97-26.33) | 256.34 (197.12-361.36) |
| North Africa and Middle East | 2011 | 3691.27 (2912.26-4636.04) | 18.68 (14.25-25.76) | 259.2 (199.98-355.47) |
| North Africa and Middle East | 2012 | 3689.94 (2931.7-4646.2) | 19.06 (14.41-26.07) | 263.2 (201.17-358.21) |
| North Africa and Middle East | 2013 | 3686.77 (2926.91-4652.05) | 19.74 (14.88-27) | 270.23 (206.07-366.28) |
| North Africa and Middle East | 2014 | 3682.94 (2937.55-4643.85) | 20.79 (15.69-27.83) | 281.79 (215.14-374.9) |
| North Africa and Middle East | 2015 | 3678.73 (2937.63-4644.81) | 21.61 (16.41-28.6) | 289.9 (222.26-382.33) |
| North Africa and Middle East | 2016 | 3641.9 (2931.67-4495.73) | 22.19 (16.9-29.01) | 295.61 (227.37-385.57) |
| North Africa and Middle East | 2017 | 3566.3 (2907.21-4363.63) | 22.36 (17.07-29.15) | 296.06 (228.19-385.4) |
| North Africa and Middle East | 2018 | 3492.01 (2856-4262.92) | 22.37 (16.95-29.06) | 295.11 (225.81-382.28) |
| North Africa and Middle East | 2019 | 3458.24 (2835.66-4230.98) | 22.79 (17.26-29.38) | 298.99 (228.66-384.81) |
| North Africa and Middle East | 2020 | 3474.35 (2852.07-4261.14) | 22.76 (17.19-29.27) | 298.01 (227.43-382.95) |
| North Africa and Middle East | 2021 | 3479.51 (2814.97-4261.91) | 22.43 (16.91-28.76) | 294.69 (224.21-377.8) |
| South Asia | 1990 | 11719.2 (9432.41-14336.82) | 32.77 (24.16-44.41) | 529.81 (392.59-708.52) |
| South Asia | 1991 | 11759.64 (9508.18-14349.18) | 32.49 (23.77-44.06) | 524.91 (386.5-703.45) |
| South Asia | 1992 | 11795.76 (9570.99-14376.9) | 33.47 (24.48-44.75) | 534.65 (393.04-705.79) |
| South Asia | 1993 | 11826.48 (9628.67-14413.16) | 33.6 (24.75-44.85) | 536.58 (397.29-708.46) |
| South Asia | 1994 | 11850.42 (9666.23-14444.19) | 34.75 (25.48-46.49) | 552.85 (407.57-730.06) |
| South Asia | 1995 | 11865.88 (9673.85-14482.08) | 37.36 (27.48-50.1) | 581.53 (430.59-770.27) |
| South Asia | 1996 | 11876.89 (9705-14472.26) | 36.82 (27.23-49.88) | 577.09 (430.8-771.47) |
| South Asia | 1997 | 11888.84 (9730.98-14453.87) | 35.62 (26.18-48.08) | 568.38 (421.31-757.13) |
| South Asia | 1998 | 11900.94 (9729.75-14464.21) | 35.68 (26.3-47.3) | 573.89 (426.21-750.23) |
| South Asia | 1999 | 11912.98 (9736.52-14485.61) | 34.56 (25.31-45.76) | 560.85 (413.01-731.15) |
| South Asia | 2000 | 11925.02 (9716.39-14498.21) | 33.49 (24.7-44.76) | 544.84 (404.58-715.45) |
| South Asia | 2001 | 11937.58 (9747.02-14510.13) | 33.62 (25.06-44.32) | 547.27 (411.16-708.58) |
| South Asia | 2002 | 11952.18 (9752.92-14524.27) | 34.47 (25.69-45.06) | 559.51 (419.17-719.28) |
| South Asia | 2003 | 11970.2 (9761.13-14566.67) | 35.6 (26.62-46.43) | 571 (430.06-733.05) |
| South Asia | 2004 | 11992.89 (9754.84-14617.12) | 36.55 (27.21-49.25) | 578.33 (434.68-767.43) |
| South Asia | 2005 | 12021.93 (9757.15-14645.26) | 36.81 (27.21-48.43) | 582.7 (434.63-754.89) |
| South Asia | 2006 | 12106.13 (9845.16-14726.16) | 38.42 (28.64-49.61) | 602.96 (455.31-766.79) |
| South Asia | 2007 | 12261.76 (10002.15-14916.22) | 40.05 (29.76-51.15) | 623.41 (468.19-785.37) |
| South Asia | 2008 | 12443.19 (10171.23-15142.69) | 40.59 (29.93-51.13) | 630.81 (469.36-784.95) |
| South Asia | 2009 | 12603.97 (10288.41-15331.61) | 39.23 (29.16-50.12) | 616.99 (462.61-775.92) |
| South Asia | 2010 | 12698.1 (10357.94-15433.16) | 38.27 (28.39-49.39) | 606.39 (453.49-771.79) |
| South Asia | 2011 | 12742.65 (10427.6-15492.39) | 38.3 (28.51-48.99) | 608.94 (457.2-767.19) |
| South Asia | 2012 | 12787.85 (10479.54-15517.44) | 39.4 (29.02-50.64) | 622.46 (462.29-784.89) |
| South Asia | 2013 | 12830.08 (10532.99-15545.4) | 42.23 (31.1-52.83) | 647.29 (480.64-804.3) |
| South Asia | 2014 | 12867.07 (10580.16-15546.46) | 43.96 (32.25-54.06) | 658.3 (486.84-808.66) |
| South Asia | 2015 | 12894.78 (10601.7-15615.56) | 44.09 (32.51-55.1) | 664.13 (493.69-821.15) |
| South Asia | 2016 | 12943.09 (10649.8-15602.46) | 44.28 (32.37-55.58) | 672.85 (496.41-830.16) |
| South Asia | 2017 | 13013.57 (10727.09-15647.3) | 45.49 (33.47-56.89) | 696.89 (515.5-857.36) |
| South Asia | 2018 | 13083.73 (10796.4-15705.92) | 45.48 (33.63-57.12) | 706.86 (525.33-875.65) |
| South Asia | 2019 | 13130.74 (10785.33-15772.03) | 45.61 (33.47-57.65) | 708.84 (523.37-884.74) |
| South Asia | 2020 | 13171.89 (10747.67-15987.44) | 44.74 (33.02-56.09) | 695.37 (515.14-862.05) |
| South Asia | 2021 | 13419.06 (10961.14-16321.66) | 43.76 (32.63-54.81) | 681.45 (508.59-848.46) |
| East Asia | 1990 | 2281.2 (1825.06-2790.93) | 6.69 (4.32-9.16) | 96.66 (63.2-131.34) |
| East Asia | 1991 | 2279.46 (1831.4-2786.57) | 6.63 (4.26-8.84) | 95.66 (62.27-127) |
| East Asia | 1992 | 2278.94 (1841.73-2774.87) | 6.46 (4.27-8.51) | 93.24 (62.64-121.83) |
| East Asia | 1993 | 2279.4 (1847.44-2766.56) | 6.48 (4.27-8.32) | 93.04 (62.34-118.66) |
| East Asia | 1994 | 2280.54 (1849.35-2767.95) | 6.4 (4.29-8.27) | 91.61 (62.33-118.56) |
| East Asia | 1995 | 2282.04 (1855.38-2770.3) | 6.38 (4.3-8.14) | 91.26 (62.43-116.56) |
| East Asia | 1996 | 2286.26 (1860.14-2775.96) | 6.29 (4.31-8) | 90.06 (62.33-114.54) |
| East Asia | 1997 | 2294.07 (1866.97-2782.84) | 6.2 (4.19-7.84) | 88.61 (60.69-111.66) |
| East Asia | 1998 | 2302.98 (1877.47-2801.02) | 6.32 (4.29-8.06) | 90.12 (62-114.52) |
| East Asia | 1999 | 2310.5 (1886.6-2819.49) | 6.37 (4.26-8.12) | 90.73 (61.71-115.04) |
| East Asia | 2000 | 2314.3 (1890.47-2824.82) | 6.62 (4.38-8.29) | 93.68 (63.1-116.82) |
| East Asia | 2001 | 2315.73 (1888.45-2822.17) | 7.01 (4.48-8.86) | 99.06 (64.15-124.39) |
| East Asia | 2002 | 2317.44 (1881.82-2820.33) | 6.81 (4.41-8.58) | 96.1 (63.32-120.12) |
| East Asia | 2003 | 2319.23 (1878.03-2827.33) | 6.44 (4.44-7.97) | 90.79 (63.16-111.53) |
| East Asia | 2004 | 2320.85 (1873.64-2835.2) | 6.32 (4.55-7.73) | 88.59 (63.98-107.95) |
| East Asia | 2005 | 2322.09 (1861.25-2836.47) | 6.05 (4.53-7.37) | 84.19 (63.31-102.6) |
| East Asia | 2006 | 2324.42 (1868.63-2826.59) | 5.55 (4.27-6.8) | 76.87 (59.57-94.43) |
| East Asia | 2007 | 2328.46 (1881.66-2825.34) | 5.33 (4.19-6.74) | 73.2 (58.28-93.62) |
| East Asia | 2008 | 2332.57 (1889.03-2831.06) | 6.17 (4.92-7.88) | 82.48 (66.79-106.48) |
| East Asia | 2009 | 2334.92 (1894-2832.05) | 6.38 (5.1-8.22) | 84.6 (69.1-110.22) |
| East Asia | 2010 | 2333.56 (1891.08-2824.59) | 6.23 (4.98-8.21) | 82.32 (66.9-110.29) |
| East Asia | 2011 | 2324.35 (1886.77-2814.43) | 6.02 (4.82-8.11) | 79.23 (64.57-108.38) |
| East Asia | 2012 | 2308.24 (1871.88-2799.93) | 5.63 (4.45-7.68) | 74.4 (59.99-103.38) |
| East Asia | 2013 | 2290.45 (1851.42-2786.23) | 5.4 (4.24-7.43) | 71.21 (56.78-99.94) |
| East Asia | 2014 | 2275.09 (1832.09-2771.56) | 5.36 (4.18-7.44) | 70.2 (55.73-99.2) |
| East Asia | 2015 | 2266.22 (1818.09-2768.79) | 5.18 (4.01-7.27) | 68.07 (53.69-97.55) |
| East Asia | 2016 | 2262.76 (1821.29-2758.16) | 5.25 (4.07-7.35) | 68.45 (53.93-97.95) |
| East Asia | 2017 | 2260.55 (1826.94-2754.47) | 5.29 (4.08-7.43) | 68.73 (53.83-98.74) |
| East Asia | 2018 | 2259.96 (1833.04-2748.23) | 5.25 (4.01-7.39) | 68.56 (53.07-98.5) |
| East Asia | 2019 | 2261.46 (1838.59-2755.01) | 5.18 (3.93-7.36) | 67.82 (52-98.35) |
| East Asia | 2020 | 2282.81 (1850.2-2790.62) | 5.04 (3.73-7.27) | 66.25 (49.81-97.25) |
| East Asia | 2021 | 2305.79 (1827.03-2822.56) | 5.12 (3.78-7.34) | 67.05 (50.29-98.22) |
| Oceania | 1990 | 3452.05 (2708.91-4378.91) | 17.71 (10.92-28.84) | 249.33 (152.96-405) |
| Oceania | 1991 | 3453.47 (2745.93-4345.02) | 17.94 (11.11-28.87) | 251.85 (155.6-404.8) |
| Oceania | 1992 | 3454.01 (2772.35-4340.75) | 17.88 (11.06-28.53) | 251.21 (154.58-399.63) |
| Oceania | 1993 | 3454.55 (2776.83-4341.18) | 17.57 (10.89-28.14) | 248.31 (152.98-395.78) |
| Oceania | 1994 | 3455.11 (2767.52-4352.15) | 17.33 (10.78-27.73) | 245.7 (151.91-391.21) |
| Oceania | 1995 | 3455.77 (2735.71-4364.01) | 17.54 (10.97-27.82) | 247.87 (153.92-391.11) |
| Oceania | 1996 | 3457.43 (2768.49-4334.86) | 17.73 (11.14-28.35) | 250.17 (156.91-398.49) |
| Oceania | 1997 | 3457.38 (2772.29-4307.71) | 17.86 (11.28-28.49) | 251.57 (158.79-400.38) |
| Oceania | 1998 | 3457.47 (2762.14-4278.98) | 17.89 (11.45-27.81) | 251.48 (160.72-391.89) |
| Oceania | 1999 | 3458.62 (2751.97-4283.1) | 17.82 (11.52-27.11) | 250.55 (161.44-381.34) |
| Oceania | 2000 | 3461.67 (2728.41-4286.66) | 17.7 (11.57-26.8) | 249.32 (163.05-376.93) |
| Oceania | 2001 | 3474.72 (2756.92-4290.1) | 17.24 (11.45-26.29) | 243.59 (161.86-372.76) |
| Oceania | 2002 | 3497.72 (2827.12-4301.6) | 16.85 (11.28-26.11) | 239.09 (159.98-371.91) |
| Oceania | 2003 | 3523.61 (2866.36-4358.98) | 16.56 (11.13-25.96) | 235.98 (158.42-370.51) |
| Oceania | 2004 | 3545.09 (2882.41-4416.26) | 16.31 (10.96-25.97) | 233.43 (156.47-371.3) |
| Oceania | 2005 | 3554.83 (2866.81-4457.59) | 16.48 (10.92-27.15) | 236.05 (156.13-388.48) |
| Oceania | 2006 | 3553.88 (2888.15-4403.83) | 16.62 (10.94-28.14) | 237.38 (155.93-402.02) |
| Oceania | 2007 | 3546.37 (2903.59-4359.43) | 16.4 (10.69-28.1) | 233.98 (151.95-400.56) |
| Oceania | 2008 | 3536.48 (2901.23-4331.49) | 16 (10.38-27.47) | 228.88 (148.64-393.23) |
| Oceania | 2009 | 3527.45 (2876.02-4325.24) | 15.6 (10.1-26.49) | 223.41 (144.95-379.81) |
| Oceania | 2010 | 3522.57 (2836.38-4338.61) | 15.54 (10.05-26.23) | 222.18 (143.48-375.03) |
| Oceania | 2011 | 3524.65 (2876.13-4300.39) | 15.8 (10.09-26.14) | 225.05 (143.87-371.62) |
| Oceania | 2012 | 3529.2 (2899.13-4291.26) | 15.75 (9.99-26.8) | 224.27 (142.43-382.69) |
| Oceania | 2013 | 3536.12 (2895.42-4301.18) | 15.52 (9.87-26.9) | 221.66 (140.89-385.66) |
| Oceania | 2014 | 3543.62 (2892.86-4357.49) | 15.49 (9.89-26.75) | 221.59 (141.47-385.08) |
| Oceania | 2015 | 3549.4 (2870.09-4395.47) | 15.31 (9.79-26.65) | 220.12 (141.08-383.3) |
| Oceania | 2016 | 3547.31 (2886.03-4385.61) | 15.39 (9.85-26.56) | 220.54 (141.34-382.29) |
| Oceania | 2017 | 3543.14 (2881.09-4375.04) | 15.46 (9.87-26.41) | 221.47 (141.4-380.13) |
| Oceania | 2018 | 3539.59 (2864.63-4386.35) | 15.49 (9.89-26.26) | 221.53 (141.6-377.2) |
| Oceania | 2019 | 3539.43 (2828.32-4429.47) | 15.47 (9.93-25.96) | 221.5 (142.19-372.08) |
| Oceania | 2020 | 3585.18 (2898.79-4479.62) | 14.82 (9.35-25.44) | 214.66 (135.66-370.47) |
| Oceania | 2021 | 3594.48 (2824.88-4522.33) | 15.12 (9.7-25.44) | 217.56 (139.58-371.66) |
| Southeast Asia | 1990 | 5777.3 (4721.9-6989.23) | 18.96 (13.59-29.98) | 288.7 (211.7-443.51) |
| Southeast Asia | 1991 | 5783.6 (4743.06-6974.49) | 19.15 (13.71-30.06) | 291.15 (213.39-444.46) |
| Southeast Asia | 1992 | 5788.7 (4740.96-6993.31) | 19.4 (14.06-30.08) | 294.71 (218.74-444.76) |
| Southeast Asia | 1993 | 5793.36 (4760.88-6994.07) | 19.69 (14.25-30.47) | 299.07 (221.22-449.91) |
| Southeast Asia | 1994 | 5797.36 (4780.71-7006.01) | 19.99 (14.51-30.51) | 302.9 (224.75-449.12) |
| Southeast Asia | 1995 | 5800.81 (4780.39-7000.92) | 20.29 (14.75-31.03) | 306.89 (227.69-456.1) |
| Southeast Asia | 1996 | 5807.71 (4789.82-7001.12) | 20.76 (15.1-31.58) | 313.39 (232.73-463.67) |
| Southeast Asia | 1997 | 5816.56 (4809.33-7002.62) | 20.9 (15.26-31.74) | 315.17 (234.89-464.8) |
| Southeast Asia | 1998 | 5828.13 (4819.45-6980.75) | 20.89 (15.29-32.14) | 315.14 (236.28-468.71) |
| Southeast Asia | 1999 | 5840.62 (4839.01-6972.01) | 21.34 (15.68-32.4) | 320.99 (241.37-473.37) |
| Southeast Asia | 2000 | 5852.48 (4841.56-6985.98) | 21.65 (15.88-33.15) | 324.86 (243.76-480.89) |
| Southeast Asia | 2001 | 5869.79 (4866.94-7010.89) | 21.72 (15.99-32.97) | 325.28 (245.06-477.67) |
| Southeast Asia | 2002 | 5897.73 (4883.45-7070.84) | 22.57 (16.58-33.9) | 336.41 (252.48-489.94) |
| Southeast Asia | 2003 | 5927.96 (4916.6-7136.14) | 23.19 (17.09-34.37) | 344.26 (259.2-496.57) |
| Southeast Asia | 2004 | 5953.58 (4950.72-7204.34) | 23.88 (17.65-34.75) | 353.25 (267.02-500.84) |
| Southeast Asia | 2005 | 5966.81 (4959.96-7256.67) | 24.35 (18.08-35.34) | 359.04 (272.64-507.86) |
| Southeast Asia | 2006 | 5938.79 (4939.27-7179.6) | 25.27 (18.7-35.93) | 370.11 (279.47-515.39) |
| Southeast Asia | 2007 | 5868.73 (4869.02-7070.63) | 25.61 (18.91-36.31) | 373.23 (281.68-518.91) |
| Southeast Asia | 2008 | 5785.07 (4802.91-6974.42) | 25.92 (19.19-36.58) | 376.54 (284.59-520.75) |
| Southeast Asia | 2009 | 5715.79 (4726.8-6876.86) | 26.15 (19.35-37.07) | 378.71 (287.39-526.31) |
| Southeast Asia | 2010 | 5687.61 (4686.54-6846.39) | 26.34 (19.29-37.54) | 380.1 (286.26-530.69) |
| Southeast Asia | 2011 | 5697.23 (4724.4-6847.25) | 26.51 (19.3-37.8) | 381.63 (285.76-532.74) |
| Southeast Asia | 2012 | 5718.88 (4768.67-6865.93) | 26.87 (19.65-37.78) | 385.69 (288.63-530.66) |
| Southeast Asia | 2013 | 5747.24 (4811.96-6884.98) | 27.64 (20.46-37.92) | 394.68 (298.66-531.21) |
| Southeast Asia | 2014 | 5772.83 (4832.56-6937.59) | 28.68 (21.15-38.71) | 407.24 (305.53-540.88) |
| Southeast Asia | 2015 | 5787.3 (4846.56-6970.58) | 29.65 (21.57-39.14) | 418.33 (310.62-544.2) |
| Southeast Asia | 2016 | 5791.2 (4851.24-6932.99) | 31.08 (22.25-40.32) | 434.42 (318.58-555.41) |
| Southeast Asia | 2017 | 5789.24 (4854.93-6915.49) | 32.41 (22.53-42.34) | 448.72 (319.66-576.47) |
| Southeast Asia | 2018 | 5787.74 (4832.66-6922.64) | 33.55 (22.97-44.06) | 461.13 (324.73-595.14) |
| Southeast Asia | 2019 | 5792.12 (4804.71-6948.72) | 34.68 (23.45-46.31) | 473.16 (328.98-618.52) |
| Southeast Asia | 2020 | 5848.73 (4854.4-6944.32) | 34.59 (23.26-46.85) | 470.4 (326.12-622.87) |
| Southeast Asia | 2021 | 5712.97 (4748.78-6923.35) | 35.19 (23.4-47.64) | 478.03 (328-634.66) |
| Central Sub-Saharan Africa | 1990 | 4426.25 (3460.15-5556.72) | 15.47 (8.19-37.93) | 238.84 (129.04-559.63) |
| Central Sub-Saharan Africa | 1991 | 4425.46 (3505.53-5472.4) | 15.52 (8.23-38.22) | 239.71 (129.65-562.24) |
| Central Sub-Saharan Africa | 1992 | 4426.43 (3532.11-5439.14) | 15.52 (8.27-37.79) | 239.68 (130.08-558.08) |
| Central Sub-Saharan Africa | 1993 | 4428.83 (3546.72-5423.54) | 15.6 (8.3-37.96) | 240.87 (130.88-560.15) |
| Central Sub-Saharan Africa | 1994 | 4432.38 (3555.06-5460.61) | 15.73 (8.44-38.05) | 242.69 (132.48-560.72) |
| Central Sub-Saharan Africa | 1995 | 4436.72 (3538.02-5510.62) | 15.78 (8.47-37.62) | 243.58 (133.03-555.8) |
| Central Sub-Saharan Africa | 1996 | 4444.92 (3578.04-5494.19) | 16.03 (8.66-37.47) | 247.79 (136.02-556.95) |
| Central Sub-Saharan Africa | 1997 | 4458.12 (3626.26-5527.5) | 15.65 (8.42-35.94) | 241.55 (132.99-535.33) |
| Central Sub-Saharan Africa | 1998 | 4473.06 (3651.43-5563.36) | 15.62 (8.38-35.74) | 241.29 (131.69-534.95) |
| Central Sub-Saharan Africa | 1999 | 4486.4 (3621.74-5618.62) | 15.53 (8.35-35.79) | 239.96 (131.38-538.49) |
| Central Sub-Saharan Africa | 2000 | 4494.84 (3573.03-5673.05) | 15.54 (8.3-35.74) | 240.19 (131.05-537.97) |
| Central Sub-Saharan Africa | 2001 | 4500.42 (3621.56-5628.12) | 15.48 (8.29-35.45) | 239.44 (130.92-534.16) |
| Central Sub-Saharan Africa | 2002 | 4506.78 (3653.08-5612.57) | 15.33 (8.1-35.46) | 237.26 (127.97-534.43) |
| Central Sub-Saharan Africa | 2003 | 4512.99 (3645.49-5608.94) | 15.38 (8.07-35.64) | 238.08 (127.45-539.53) |
| Central Sub-Saharan Africa | 2004 | 4518.14 (3611.61-5622.58) | 15.26 (8.04-35.44) | 236.18 (127.05-539.62) |
| Central Sub-Saharan Africa | 2005 | 4521.29 (3549.12-5661.06) | 15.05 (7.85-35.1) | 232.75 (124.05-536.68) |
| Central Sub-Saharan Africa | 2006 | 4523.58 (3599.36-5631.65) | 14.98 (7.84-34.49) | 231.56 (123.02-524.77) |
| Central Sub-Saharan Africa | 2007 | 4526.57 (3623.82-5576.15) | 14.85 (7.7-33.93) | 229.59 (121.14-516.93) |
| Central Sub-Saharan Africa | 2008 | 4530.03 (3656.57-5583.81) | 14.9 (7.7-33.76) | 230.12 (121.16-511.98) |
| Central Sub-Saharan Africa | 2009 | 4533.7 (3642.54-5600.12) | 14.89 (7.68-33.49) | 229.74 (120.68-507.97) |
| Central Sub-Saharan Africa | 2010 | 4537.4 (3586.03-5623.57) | 14.87 (7.68-33.57) | 229.42 (120.32-510.28) |
| Central Sub-Saharan Africa | 2011 | 4544.98 (3664.68-5602.44) | 14.94 (7.72-34.02) | 230.21 (120.93-518.35) |
| Central Sub-Saharan Africa | 2012 | 4558.12 (3697.2-5610.43) | 15 (7.71-34.03) | 231 (120.51-519.98) |
| Central Sub-Saharan Africa | 2013 | 4573.44 (3705.22-5624.47) | 15.14 (7.73-34.69) | 232.71 (120.75-529.06) |
| Central Sub-Saharan Africa | 2014 | 4587.45 (3688.96-5653.88) | 15.21 (7.78-34.82) | 233.54 (120.96-531.99) |
| Central Sub-Saharan Africa | 2015 | 4596.48 (3669.12-5717.18) | 15.29 (7.82-35.35) | 234.27 (121.26-539.84) |
| Central Sub-Saharan Africa | 2016 | 4608.25 (3725.65-5680.54) | 15.38 (7.92-35.06) | 235.32 (122.43-534.65) |
| Central Sub-Saharan Africa | 2017 | 4625.96 (3775.7-5650.88) | 15.49 (7.99-35.18) | 236.56 (123.32-536.03) |
| Central Sub-Saharan Africa | 2018 | 4643.11 (3741.62-5733.37) | 15.58 (8.04-34.75) | 237.43 (123.5-526.81) |
| Central Sub-Saharan Africa | 2019 | 4653.15 (3669.7-5861.89) | 15.68 (8.13-34.69) | 238.31 (125.56-524.55) |
| Central Sub-Saharan Africa | 2020 | 4647.71 (3640.71-5840.11) | 15.89 (8.24-34.55) | 241.33 (127.16-520.73) |
| Central Sub-Saharan Africa | 2021 | 4655.49 (3693.55-5823.91) | 16 (8.27-34.27) | 242.88 (127.27-517.49) |
| Eastern Sub-Saharan Africa | 1990 | 5041.12 (4105.41-6165.85) | 58.79 (44-85.16) | 989 (747.93-1410.98) |
| Eastern Sub-Saharan Africa | 1991 | 5031.89 (4111.48-6146.03) | 58.36 (43.71-85.24) | 980.25 (742.35-1416.46) |
| Eastern Sub-Saharan Africa | 1992 | 5024.76 (4110.55-6125.07) | 58.21 (43.48-84.95) | 976.45 (739.42-1407.1) |
| Eastern Sub-Saharan Africa | 1993 | 5019.92 (4108.68-6128.57) | 58.07 (43.61-84.33) | 973.63 (740.27-1396.98) |
| Eastern Sub-Saharan Africa | 1994 | 5017.81 (4102-6131.12) | 57.92 (43.67-83.7) | 970.51 (740.17-1383.51) |
| Eastern Sub-Saharan Africa | 1995 | 5017.8 (4104.33-6141.63) | 57.74 (43.63-83.28) | 966.13 (739.62-1378.85) |
| Eastern Sub-Saharan Africa | 1996 | 5022.39 (4114.81-6131.4) | 57.45 (43.27-82.38) | 959.73 (731.68-1359.59) |
| Eastern Sub-Saharan Africa | 1997 | 5032.81 (4118.74-6128.07) | 57.26 (43.24-81.47) | 955.45 (730.72-1344.19) |
| Eastern Sub-Saharan Africa | 1998 | 5045.16 (4119.28-6141.86) | 57.2 (43.1-81.06) | 953.2 (726.9-1336.09) |
| Eastern Sub-Saharan Africa | 1999 | 5056.52 (4121.42-6181.16) | 56.88 (43.15-80.7) | 946.29 (728.03-1327.89) |
| Eastern Sub-Saharan Africa | 2000 | 5063.45 (4108.28-6221.96) | 56.55 (42.84-80.58) | 938.62 (721.15-1324.18) |
| Eastern Sub-Saharan Africa | 2001 | 5066.34 (4123.03-6187.87) | 55.6 (42.19-79.12) | 921.03 (708.66-1298.13) |
| Eastern Sub-Saharan Africa | 2002 | 5069.04 (4128.79-6167.37) | 56.04 (42.89-79.47) | 926.04 (718.61-1302.97) |
| Eastern Sub-Saharan Africa | 2003 | 5071.17 (4139.51-6163.26) | 56.2 (42.95-78.68) | 927.19 (718.33-1288.67) |
| Eastern Sub-Saharan Africa | 2004 | 5074.14 (4127.52-6179.92) | 56.37 (43.34-78.08) | 928.08 (722.55-1276.66) |
| Eastern Sub-Saharan Africa | 2005 | 5079.03 (4114.82-6183.38) | 56.31 (43.5-77.75) | 924.68 (721.72-1268.64) |
| Eastern Sub-Saharan Africa | 2006 | 5091.33 (4145.95-6196.68) | 56.13 (43.4-78.21) | 919.26 (719.55-1274.58) |
| Eastern Sub-Saharan Africa | 2007 | 5112.37 (4177.99-6223.33) | 55.58 (43.28-76.49) | 908.52 (714.69-1244.99) |
| Eastern Sub-Saharan Africa | 2008 | 5136.4 (4211.13-6248.22) | 55.48 (43.14-76.34) | 904.88 (711.29-1238.53) |
| Eastern Sub-Saharan Africa | 2009 | 5158.72 (4238.32-6275.6) | 55.69 (43.5-76.22) | 905.83 (713.36-1233.37) |
| Eastern Sub-Saharan Africa | 2010 | 5174.65 (4243.81-6295.55) | 56.05 (43.95-76.49) | 908.71 (719.84-1231.34) |
| Eastern Sub-Saharan Africa | 2011 | 5186.08 (4258.77-6299.7) | 58.07 (45.55-77.17) | 939.66 (744.13-1241.84) |
| Eastern Sub-Saharan Africa | 2012 | 5198.57 (4263.65-6305.93) | 59.45 (46.69-76.93) | 958.46 (760.58-1234.4) |
| Eastern Sub-Saharan Africa | 2013 | 5213.09 (4274.75-6335.62) | 60.01 (47-77.74) | 964.85 (763.24-1242.65) |
| Eastern Sub-Saharan Africa | 2014 | 5226.7 (4266.29-6374.2) | 60.76 (47.46-78.62) | 974.65 (767.36-1252.41) |
| Eastern Sub-Saharan Africa | 2015 | 5236.18 (4249.24-6420.79) | 61.55 (48.15-79.51) | 984.6 (776.14-1261.79) |
| Eastern Sub-Saharan Africa | 2016 | 5251.3 (4274.1-6410.81) | 62.7 (49.02-79.63) | 1001.8 (787.89-1266.52) |
| Eastern Sub-Saharan Africa | 2017 | 5270.91 (4278.83-6422.28) | 63.15 (48.84-80.62) | 1006.71 (783.32-1278.02) |
| Eastern Sub-Saharan Africa | 2018 | 5289.77 (4275.3-6441.2) | 63.77 (49.24-81.84) | 1013.61 (788.38-1291.51) |
| Eastern Sub-Saharan Africa | 2019 | 5302.68 (4269.1-6467.12) | 64.34 (50.06-83.11) | 1019.96 (801.26-1308.27) |
| Eastern Sub-Saharan Africa | 2020 | 5313 (4367.14-6427.44) | 65.18 (50.17-83.94) | 1033.26 (802.91-1318.72) |
| Eastern Sub-Saharan Africa | 2021 | 5298.85 (4287.52-6510.28) | 65.42 (49.99-84.93) | 1039.71 (803.58-1343.47) |
| Southern Sub-Saharan Africa | 1990 | 3792.77 (3011.83-4741.98) | 6.87 (3.43-10.57) | 94.03 (46.88-141.6) |
| Southern Sub-Saharan Africa | 1991 | 3765.99 (3014.94-4669.09) | 6.89 (3.42-10.41) | 93.84 (46.78-139.42) |
| Southern Sub-Saharan Africa | 1992 | 3744.79 (3014.95-4615.73) | 7.38 (3.68-11.07) | 99.77 (49.71-147.09) |
| Southern Sub-Saharan Africa | 1993 | 3729.54 (3007.37-4568.55) | 7.38 (3.67-11.11) | 99.57 (49.41-147.38) |
| Southern Sub-Saharan Africa | 1994 | 3720.74 (2997.2-4537.03) | 7.83 (3.9-11.66) | 105.41 (52.46-154.12) |
| Southern Sub-Saharan Africa | 1995 | 3718.91 (2997.55-4506.03) | 7.8 (3.88-11.65) | 104.84 (52.39-153.73) |
| Southern Sub-Saharan Africa | 1996 | 3730.95 (3018.88-4530.39) | 8.44 (4.17-12.62) | 113.69 (56.21-166.79) |
| Southern Sub-Saharan Africa | 1997 | 3757.39 (3043.66-4568.83) | 9.17 (4.55-13.44) | 123.74 (60.77-177.7) |
| Southern Sub-Saharan Africa | 1998 | 3788.75 (3063.92-4610.93) | 9.41 (4.59-13.66) | 127.32 (61.61-181.04) |
| Southern Sub-Saharan Africa | 1999 | 3815.61 (3087.11-4657.99) | 9.63 (4.64-13.77) | 130.58 (62.51-183.12) |
| Southern Sub-Saharan Africa | 2000 | 3828.65 (3091.09-4689.05) | 9.87 (4.72-14) | 134.67 (63.59-187.27) |
| Southern Sub-Saharan Africa | 2001 | 3819.23 (3103.02-4658.31) | 10.08 (4.75-14.2) | 137.4 (63.93-190.08) |
| Southern Sub-Saharan Africa | 2002 | 3792.05 (3087.69-4600.81) | 10.23 (4.77-14.32) | 140.18 (64.61-192.7) |
| Southern Sub-Saharan Africa | 2003 | 3757.67 (3064.99-4556.77) | 10.28 (4.76-14.35) | 141.44 (64.76-194.61) |
| Southern Sub-Saharan Africa | 2004 | 3727.23 (3044.15-4519.36) | 10.14 (4.7-14.28) | 140.43 (64.72-195.32) |
| Southern Sub-Saharan Africa | 2005 | 3711.9 (3023.86-4503.21) | 9.85 (4.53-14.01) | 137.34 (63.68-193.25) |
| Southern Sub-Saharan Africa | 2006 | 3711.51 (3036.77-4494.27) | 9.62 (4.4-14) | 135.08 (62.53-194.56) |
| Southern Sub-Saharan Africa | 2007 | 3716.56 (3039.88-4491.9) | 9.37 (4.28-13.9) | 132.06 (61.64-193.71) |
| Southern Sub-Saharan Africa | 2008 | 3722.74 (3045.43-4485.36) | 9.27 (4.31-14.07) | 131.32 (62.52-197.47) |
| Southern Sub-Saharan Africa | 2009 | 3726.76 (3056.93-4492.47) | 9.05 (4.25-14.08) | 128.81 (62.34-198.11) |
| Southern Sub-Saharan Africa | 2010 | 3725.14 (3042.92-4496.34) | 8.93 (4.29-14.09) | 127.67 (63.18-198.99) |
| Southern Sub-Saharan Africa | 2011 | 3695.95 (3032.69-4449.57) | 8.73 (4.28-14.18) | 124.66 (63.16-199.74) |
| Southern Sub-Saharan Africa | 2012 | 3635.32 (2993.92-4382.11) | 8.69 (4.34-14.3) | 123.94 (64.25-200.84) |
| Southern Sub-Saharan Africa | 2013 | 3564.51 (2935.42-4308.47) | 8.64 (4.39-14.42) | 122.97 (64.66-202.18) |
| Southern Sub-Saharan Africa | 2014 | 3505.35 (2888.35-4255.78) | 8.73 (4.54-14.78) | 124.27 (66.45-207.05) |
| Southern Sub-Saharan Africa | 2015 | 3478.42 (2854.46-4248.84) | 8.81 (4.6-15.02) | 125.34 (67.45-210.15) |
| Southern Sub-Saharan Africa | 2016 | 3493.77 (2871.27-4267.61) | 8.79 (4.64-15.16) | 125.07 (67.83-212.16) |
| Southern Sub-Saharan Africa | 2017 | 3530.99 (2878.48-4327.96) | 8.73 (4.57-15) | 123.86 (66.71-209.25) |
| Southern Sub-Saharan Africa | 2018 | 3570.08 (2902.05-4381.89) | 8.76 (4.61-14.96) | 123.91 (67.23-208.04) |
| Southern Sub-Saharan Africa | 2019 | 3590.92 (2900.69-4423.89) | 8.65 (4.56-14.76) | 121.83 (66.28-204.09) |
| Southern Sub-Saharan Africa | 2020 | 3548.11 (2821.22-4333.06) | 8.94 (4.7-15.21) | 125.92 (68.26-210.83) |
| Southern Sub-Saharan Africa | 2021 | 3694.55 (2943.66-4593.85) | 8.78 (4.66-14.95) | 124.46 (68.17-208.64) |
| Western Sub-Saharan Africa | 1990 | 6050.19 (4879.39-7470.24) | 14.93 (10.21-21.97) | 223.92 (153.93-331.91) |
| Western Sub-Saharan Africa | 1991 | 6049.61 (4898.91-7461.72) | 14.94 (10.24-21.78) | 224.08 (154.59-328.15) |
| Western Sub-Saharan Africa | 1992 | 6046.7 (4920.2-7459.6) | 15.04 (10.35-21.97) | 225.35 (155.63-329.88) |
| Western Sub-Saharan Africa | 1993 | 6043.11 (4930.44-7440.93) | 15.08 (10.43-22.44) | 225.85 (156.74-336.28) |
| Western Sub-Saharan Africa | 1994 | 6038.59 (4935.28-7425.32) | 15.11 (10.32-22.48) | 226.18 (155.46-336.84) |
| Western Sub-Saharan Africa | 1995 | 6033.31 (4928.45-7409.94) | 15.03 (10.36-22.57) | 225.03 (156.19-337.62) |
| Western Sub-Saharan Africa | 1996 | 6028.84 (4921.73-7416.49) | 14.88 (10.25-22.79) | 222.76 (154.06-340.61) |
| Western Sub-Saharan Africa | 1997 | 6026.8 (4918.53-7430.19) | 14.77 (10.22-22.67) | 221.08 (154.46-339.73) |
| Western Sub-Saharan Africa | 1998 | 6024.94 (4920.29-7436.57) | 14.61 (10.13-22.6) | 218.86 (153.61-337.87) |
| Western Sub-Saharan Africa | 1999 | 6023.2 (4930.51-7446.48) | 14.36 (10.05-22.81) | 215.08 (151.73-340.67) |
| Western Sub-Saharan Africa | 2000 | 6021.41 (4929.83-7442.01) | 14.21 (9.81-22.56) | 212.69 (149.37-335.58) |
| Western Sub-Saharan Africa | 2001 | 6021.67 (4937.82-7428.21) | 14.01 (9.77-22.62) | 210.22 (148.34-336.48) |
| Western Sub-Saharan Africa | 2002 | 6024.78 (4945.07-7416.37) | 14.04 (9.84-23.02) | 210.15 (149.88-341.57) |
| Western Sub-Saharan Africa | 2003 | 6029.54 (4957.88-7411.77) | 13.94 (9.76-22.82) | 208.72 (148.25-338.04) |
| Western Sub-Saharan Africa | 2004 | 6034.33 (4967.6-7406.67) | 13.96 (9.87-22.81) | 208.71 (150.08-337.75) |
| Western Sub-Saharan Africa | 2005 | 6036.98 (4965.69-7403.18) | 13.99 (9.81-23.39) | 208.87 (148.8-345.63) |
| Western Sub-Saharan Africa | 2006 | 6038.75 (4963.44-7394.12) | 14.12 (9.98-23.87) | 210.26 (151.73-350.61) |
| Western Sub-Saharan Africa | 2007 | 6041.97 (4962.9-7385.83) | 14.04 (9.8-23.49) | 208.96 (147.88-344.72) |
| Western Sub-Saharan Africa | 2008 | 6045.95 (4956.69-7379.61) | 14.16 (10.01-23.79) | 209.92 (150.67-350.1) |
| Western Sub-Saharan Africa | 2009 | 6049.46 (4953.37-7393.38) | 14.23 (10.1-23.85) | 210.99 (151.89-349.08) |
| Western Sub-Saharan Africa | 2010 | 6049.44 (4935.69-7406.72) | 14.4 (10.29-24.29) | 213.55 (154.58-356.06) |
| Western Sub-Saharan Africa | 2011 | 6051.1 (4954.86-7393.76) | 14.64 (10.44-24.64) | 217.05 (156.35-361.65) |
| Western Sub-Saharan Africa | 2012 | 6057.43 (4970.78-7384.21) | 14.84 (10.69-24.68) | 219.9 (160.55-360.81) |
| Western Sub-Saharan Africa | 2013 | 6067.36 (4986.81-7385.01) | 15.1 (10.8-25.06) | 223.74 (162.25-367.06) |
| Western Sub-Saharan Africa | 2014 | 6078.43 (5003.86-7415.19) | 15.26 (10.97-25.2) | 226.4 (165.11-369.85) |
| Western Sub-Saharan Africa | 2015 | 6090.42 (5015.6-7442.77) | 15.43 (11.18-25.25) | 228.25 (167.34-368.61) |
| Western Sub-Saharan Africa | 2016 | 6111.55 (5025.62-7459.69) | 15.69 (11.36-25.33) | 232.05 (169.86-369.13) |
| Western Sub-Saharan Africa | 2017 | 6142.2 (5038.64-7512.47) | 15.92 (11.52-25.31) | 235.35 (172.45-370.16) |
| Western Sub-Saharan Africa | 2018 | 6172.14 (5046.73-7570.15) | 15.97 (11.61-25.67) | 235.63 (173.47-372.76) |
| Western Sub-Saharan Africa | 2019 | 6191.39 (5069.14-7615.07) | 15.95 (11.62-25.39) | 235.02 (172.82-369.81) |
| Western Sub-Saharan Africa | 2020 | 6182.81 (5051.58-7601.41) | 15.99 (11.54-25.65) | 235.67 (171.74-372.94) |
| Western Sub-Saharan Africa | 2021 | 6308.78 (5080.23-7819.71) | 16.08 (11.64-25.84) | 237.16 (173.15-375.2) |

Abbreviations: DALYs, disability-adjusted life-years; UI, uncertainty interval.

**Table S5. The case number and age-standardized rate of incidence of urinary tract infections in 1990 and 2021, and the temporal trends between 1990 and 2021 at the national level.**

| Location | Number of cases, 1990 | Age-standardized rate per 100 000 population, 1990 | Number of cases, 2021 | Age-standardized rate per 100 000 population, 2021 | AAPC, 1990-2021 | p value |
| --- | --- | --- | --- | --- | --- | --- |
| Afghanistan | 7535 (5693-9802) | 2944.66 (2238.56-3821.71) | 11154 (8287-14534) | 3131.94 (2333.3-4076.64) | 0.21 (0.17 to 0.24) | < 0.001 |
| Albania | 9330 (7171-11858) | 10540.1 (8094.84-13397.5) | 23464 (17825-30642) | 10719.4 (8146.27-13992.58) | 0.06 (-0.03 to 0.14) | 0.186 |
| Algeria | 17481 (13232-22345) | 3270.81 (2479.16-4177.98) | 48102 (35795-63033) | 3362.35 (2505.67-4406.42) | 0.08 (0.06 to 0.1) | < 0.001 |
| American Samoa | 32 (25-41) | 3754.03 (2893.33-4828.08) | 77 (58-100) | 4046.03 (3044.81-5208.04) | 0.24 (0.22 to 0.25) | < 0.001 |
| Andorra | 183 (143-231) | 6981.63 (5475.63-8847.71) | 490 (373-631) | 7361.23 (5644.22-9437.66) | 0.18 (0.13 to 0.23) | < 0.001 |
| Angola | 6078 (4647-7793) | 4502.92 (3446.34-5757.9) | 20711 (15938-27142) | 4569.35 (3520.87-5980.93) | 0.08 (0.01 to 0.14) | 0.021 |
| Antigua and Barbuda | 226 (163-300) | 7727.89 (5600.21-10220.7) | 432 (334-556) | 9048.36 (6983.93-11639) | 0.52 (0.47 to 0.56) | < 0.001 |
| Argentina | 176924 (124335-233833) | 10513.86 (7380.37-13904.2) | 406504 (304415-516751) | 13421.58 (10071.73-17036.3) | 0.81 (0.75 to 0.87) | < 0.001 |
| Armenia | 8390 (6341-11113) | 7263.89 (5491.92-9611.79) | 22483 (16960-29925) | 9431.87 (7119.03-12559.51) | 0.85 (0.83 to 0.86) | < 0.001 |
| Australia | 130440 (94263-171402) | 12212.73 (8820.6-16033.53) | 272526 (180278-375070) | 11522.02 (7579.58-15910.91) | -0.19 (-0.23 to -0.15) | < 0.001 |
| Austria | 65224 (48615-88655) | 8679.87 (6532.01-11640.39) | 85091 (56182-155574) | 9135.34 (6201.49-15715.4) | 0.12 (0.02 to 0.23) | 0.025 |
| Azerbaijan | 16507 (12739-21045) | 7297.67 (5621.05-9307.81) | 33383 (25237-43340) | 7816.14 (5931.62-10155.1) | 0.22 (0.14 to 0.29) | < 0.001 |
| Bahamas | 605 (463-762) | 8355.72 (6388.93-10542.36) | 1661 (1272-2157) | 9095.41 (6976.07-11818.16) | 0.28 (0.22 to 0.34) | < 0.001 |
| Bahrain | 166 (124-211) | 3139.85 (2370.85-3988.17) | 865 (641-1145) | 3351.13 (2499.3-4418.82) | 0.21 (0.2 to 0.22) | < 0.001 |
| Bangladesh | 160708 (115685-210265) | 10312.83 (7433.25-13486.18) | 645735 (477527-842256) | 11415.73 (8456.86-14874.58) | 0.31 (0.25 to 0.37) | < 0.001 |
| Barbados | 1436 (1088-1847) | 8370.83 (6333.7-10747.51) | 2518 (1902-3249) | 9230.29 (6971.68-11907.06) | 0.3 (0.24 to 0.37) | < 0.001 |
| Belarus | 71335 (55086-93240) | 9377.33 (7225.88-12267.42) | 94445 (72944-120860) | 9727.32 (7534.2-12410.74) | 0.16 (-0.02 to 0.35) | 0.089 |
| Belgium | 52540 (39500-68762) | 6058.12 (4531.85-7941.11) | 86266 (60456-115300) | 7057.36 (5060.23-9386.05) | 0.49 (0.42 to 0.56) | < 0.001 |
| Belize | 313 (225-411) | 7701.34 (5546.05-10099.49) | 972 (734-1239) | 8773.38 (6628.96-11185.03) | 0.43 (0.41 to 0.45) | < 0.001 |
| Benin | 4920 (3649-6522) | 5834.4 (4330.32-7725.95) | 12833 (9706-16747) | 6104.69 (4607.91-7970.97) | 0.13 (0.08 to 0.19) | < 0.001 |
| Bermuda | 272 (190-361) | 8469.35 (5906.69-11268.99) | 604 (421-833) | 8245.65 (5727.17-11366.77) | -0.09 (-0.14 to -0.04) | < 0.001 |
| Bhutan | 968 (754-1218) | 10944.15 (8521.18-13782.96) | 2975 (2213-3839) | 11731.43 (8735.26-15139.16) | 0.23 (0.19 to 0.27) | < 0.001 |
| Bolivia (Plurinational State of) | 16026 (12331-20465) | 11884.58 (9132.07-15185.77) | 54026 (40437-70164) | 13115.99 (9828.13-17040.97) | 0.31 (0.2 to 0.42) | < 0.001 |
| Bosnia and Herzegovina | 18352 (14205-23676) | 10266.6 (7940-13242.8) | 33490 (24543-44967) | 9587.06 (7023.46-12864.52) | -0.22 (-0.32 to -0.13) | < 0.001 |
| Botswana | 1041 (799-1331) | 4053.11 (3115.71-5166.68) | 2742 (2041-3531) | 4209.52 (3139.44-5428.5) | 0.14 (0.06 to 0.23) | 0.001 |
| Brazil | 571191 (451084-709822) | 14997.56 (11786.29-18704.6) | 2137587 (1750270-2577907) | 17338 (14202.27-20909.53) | 0.47 (0.4 to 0.53) | < 0.001 |
| Brunei Darussalam | 262 (188-364) | 7678.22 (5527.82-10625.6) | 1075 (728-1618) | 8781.85 (6004.48-13082.85) | 0.46 (0.39 to 0.54) | < 0.001 |
| Bulgaria | 66010 (50078-86922) | 10285.93 (7815.36-13484.75) | 84257 (62317-112793) | 9426.88 (6972.15-12611.79) | -0.29 (-0.32 to -0.25) | < 0.001 |
| Burkina Faso | 10667 (8160-13538) | 5740.98 (4394.31-7307.19) | 23135 (17318-30324) | 6021.08 (4511.84-7892.59) | 0.15 (0.09 to 0.2) | < 0.001 |
| Burundi | 4923 (3727-6499) | 4724.07 (3574.21-6235.08) | 8006 (6091-10373) | 4797.76 (3652.2-6224.63) | 0.06 (-0.08 to 0.2) | 0.381 |
| Cabo Verde | 691 (532-873) | 5998.52 (4622.89-7557.93) | 1306 (998-1709) | 6365.67 (4877.12-8324.08) | 0.19 (0.16 to 0.22) | < 0.001 |
| Cambodia | 10900 (8305-13996) | 5448.26 (4151.9-6990.27) | 35992 (27392-47026) | 5895.52 (4496.05-7706.72) | 0.26 (0.18 to 0.34) | < 0.001 |
| Cameroon | 10067 (7472-13270) | 5950.14 (4431.37-7827.06) | 28659 (21346-36369) | 6011.33 (4481.8-7636.49) | 0.04 (0.02 to 0.05) | < 0.001 |
| Canada | 389003 (295614-500562) | 21687.95 (16495.18-27893.57) | 839450 (588714-1095709) | 21464.28 (14984.84-28144.2) | -0.05 (-0.14 to 0.03) | 0.194 |
| Central African Republic | 1874 (1436-2392) | 4303.41 (3303.14-5504.59) | 3589 (2664-4687) | 4406.96 (3291.87-5741.21) | 0.1 (0.07 to 0.12) | < 0.001 |
| Chad | 7079 (5331-9427) | 5725.18 (4317.65-7615.46) | 11378 (8254-15119) | 5964.57 (4330.76-7924.51) | 0.12 (0.08 to 0.16) | < 0.001 |
| Chile | 59427 (46505-74547) | 12396.24 (9704.41-15538.85) | 190218 (143530-239072) | 14496.41 (10932.04-18249.75) | 0.46 (0.23 to 0.68) | < 0.001 |
| China | 842913 (672353-1031475) | 2276.26 (1815.29-2790.19) | 2455698 (1949108-3005058) | 2293.57 (1816.91-2811.98) | 0.03 (0.01 to 0.04) | 0.001 |
| Colombia | 66722 (50397-88680) | 9454.91 (7141.16-12554.83) | 267115 (203147-349963) | 9931.86 (7548.18-13017.41) | 0.16 (0.15 to 0.17) | < 0.001 |
| Comoros | 383 (292-491) | 4846.45 (3690.38-6223.98) | 1128 (858-1477) | 5195.95 (3950.7-6820.36) | 0.26 (0.22 to 0.29) | < 0.001 |
| Congo | 2149 (1603-2799) | 4417.45 (3302.38-5752.18) | 4671 (3544-6189) | 4663.47 (3537.01-6178.93) | 0.18 (0.16 to 0.2) | < 0.001 |
| Cook Islands | 17 (13-22) | 3725.24 (2774.32-4796.3) | 45 (33-59) | 3743.77 (2783.76-4914.66) | 0.03 (-0.06 to 0.11) | 0.527 |
| Costa Rica | 7271 (5440-9554) | 9714.94 (7265.81-12759.23) | 24699 (19138-31461) | 9394.15 (7277.84-11971.06) | -0.12 (-0.17 to -0.06) | < 0.001 |
| Coted'Ivoire | 7144 (5371-9339) | 5852.61 (4399.95-7669.59) | 22822 (17200-30697) | 6100.22 (4597.45-8194.69) | 0.02 (-0.02 to 0.05) | 0.281 |
| Croatia | 36052 (27067-46495) | 10965.07 (8247.15-14153.83) | 61043 (47819-76394) | 11525.62 (9004.56-14453.8) | 0.2 (0.08 to 0.33) | 0.002 |
| Cuba | 40141 (29754-52398) | 8426.43 (6241.27-10999.69) | 74839 (53456-101569) | 7944.29 (5712.12-10742.28) | -0.19 (-0.22 to -0.16) | < 0.001 |
| Cyprus | 2012 (1559-2638) | 4476.44 (3461.72-5914.24) | 4853 (3399-6707) | 4759.43 (3303.79-6633.85) | 0.12 (-0.18 to 0.43) | 0.431 |
| Czechia | 97650 (74203-127631) | 11973.32 (9110.67-15586.14) | 136872 (101302-189837) | 10711.29 (7920.67-14826.34) | -0.35 (-0.37 to -0.33) | < 0.001 |
| Democratic People's Republic of Korea | 17826 (13595-23363) | 2241.5 (1714.36-2934.02) | 38731 (28557-50531) | 2278.72 (1684.3-2971.94) | 0.04 (-0.03 to 0.1) | 0.239 |
| Democratic Republic of the Congo | 27441 (20536-35408) | 4409.04 (3315.6-5691.72) | 68274 (52228-87854) | 4685.56 (3584.11-6022.22) | 0.19 (0.14 to 0.23) | < 0.001 |
| Denmark | 30842 (23534-39245) | 6757.47 (5151.72-8581.98) | 42624 (29762-57859) | 6880.03 (4820.55-9330.82) | 0.01 (-0.2 to 0.22) | 0.929 |
| Djibouti | 220 (174-282) | 4996.4 (3932.12-6417.11) | 1045 (795-1341) | 5255.41 (4000.33-6754.3) | 0.16 (0.14 to 0.18) | < 0.001 |
| Dominica | 279 (216-356) | 8011.47 (6200.32-10220.92) | 301 (216-396) | 7597.85 (5453.15-9984.49) | -0.21 (-0.3 to -0.12) | < 0.001 |
| Dominican Republic | 12315 (9490-15684) | 8256.29 (6354.33-10504.96) | 36850 (27437-47457) | 8262.5 (6153.38-10641.63) | 0 (-0.02 to 0.03) | 0.731 |
| Ecuador | 30831 (24265-38384) | 14194.64 (11165.09-17670.77) | 142937 (95962-191919) | 18870.28 (12666.31-25360.68) | 0.91 (0.66 to 1.15) | < 0.001 |
| Egypt | 28425 (21479-37250) | 2999.57 (2263.69-3929.23) | 68129 (50388-90063) | 3143.13 (2293.06-4185.64) | 0.13 (0.12 to 0.15) | < 0.001 |
| El Salvador | 12167 (9245-15799) | 9173.27 (6962.95-11920.92) | 31121 (24019-40017) | 9778.87 (7565.9-12543.24) | 0.22 (0.2 to 0.24) | < 0.001 |
| Equatorial Guinea | 360 (275-468) | 4396.88 (3352.48-5704.71) | 989 (758-1283) | 4747.98 (3653.87-6153.67) | 0.24 (0.21 to 0.27) | < 0.001 |
| Eritrea | 1873 (1442-2383) | 4621 (3566.41-5907.51) | 5700 (4420-7337) | 4900.69 (3794.29-6333.16) | 0.19 (0.11 to 0.27) | < 0.001 |
| Estonia | 13562 (10263-17563) | 10893.94 (8224.96-14110.72) | 15432 (11973-19551) | 9529.07 (7390.45-12048.76) | -0.44 (-0.5 to -0.38) | < 0.001 |
| Eswatini | 518 (392-687) | 4037.51 (3059.58-5358.13) | 1124 (848-1441) | 4057.4 (3066.32-5216.29) | 0 (-0.07 to 0.08) | 0.939 |
| Ethiopia | 38192 (30050-47707) | 5477.88 (4307.4-6866.58) | 91839 (74145-113318) | 5568.88 (4489.55-6878.29) | 0.04 (0.02 to 0.06) | < 0.001 |
| Fiji | 441 (335-574) | 3533.44 (2687.33-4593.49) | 1187 (892-1505) | 3608.07 (2711.63-4581.65) | 0.08 (-0.06 to 0.23) | 0.25 |
| Finland | 34581 (26806-45468) | 8038.62 (6242.95-10512.85) | 47973 (33476-65238) | 7072.64 (4935.38-9640.05) | -0.42 (-0.78 to -0.07) | 0.02 |
| France | 290417 (219065-379471) | 6342.11 (4769.09-8304.08) | 442963 (311537-587316) | 6049.18 (4282.19-7993.5) | -0.16 (-0.25 to -0.08) | < 0.001 |
| Gabon | 1254 (977-1572) | 4648.14 (3620.95-5835.09) | 2078 (1566-2645) | 4948.6 (3726.5-6317.98) | 0.2 (0.16 to 0.25) | < 0.001 |
| Gambia | 762 (574-972) | 5824.74 (4387.81-7424.5) | 2461 (1840-3225) | 6169.99 (4613.93-8081.94) | 0.19 (0.16 to 0.21) | < 0.001 |
| Georgia | 26551 (19963-34456) | 7859.39 (5908.05-10202.49) | 21324 (17217-25943) | 6135.86 (4960.59-7451.58) | -0.8 (-0.85 to -0.76) | < 0.001 |
| Germany | 542756 (422509-700845) | 7106.9 (5537.09-9161.53) | 674850 (525852-844372) | 6749.16 (5231.16-8473.23) | -0.14 (-0.31 to 0.02) | 0.092 |
| Ghana | 14354 (10965-18949) | 5752.86 (4400.91-7557.48) | 44293 (34407-58339) | 6029.74 (4679.28-7940.03) | 0.13 (0.06 to 0.2) | < 0.001 |
| Greece | 47056 (34475-61330) | 6035.29 (4419.27-7866.36) | 68281 (49169-91364) | 5747.17 (4171.66-7655.93) | -0.12 (-0.18 to -0.05) | < 0.001 |
| Greenland | 222 (170-285) | 19468.16 (14948.6-24919.21) | 464 (350-614) | 19735.52 (14978.1-26052.14) | 0.02 (-0.01 to 0.05) | 0.118 |
| Grenada | 299 (213-397) | 7453.9 (5365.27-9836.55) | 465 (356-614) | 9009.86 (6899.33-11879.06) | 0.62 (0.6 to 0.65) | < 0.001 |
| Guam | 113 (86-146) | 4045.54 (3090.51-5222.09) | 437 (321-573) | 4193.6 (3085.37-5499.81) | 0.05 (0.03 to 0.08) | < 0.001 |
| Guatemala | 11132 (8395-14094) | 8514.36 (6407.58-10784.65) | 41048 (30200-53230) | 8250.56 (6060.73-10728.13) | -0.08 (-0.17 to 0.01) | 0.066 |
| Guinea | 8220 (6420-10420) | 5626.29 (4397.5-7134.21) | 12397 (9537-16157) | 5875.34 (4514.78-7657.19) | 0.13 (0.11 to 0.15) | < 0.001 |
| Guinea-Bissau | 842 (633-1085) | 5508.62 (4154.1-7096.75) | 1701 (1268-2340) | 6097.44 (4548.44-8371.81) | 0.32 (0.28 to 0.36) | < 0.001 |
| Guyana | 1123 (827-1483) | 7070.67 (5203.62-9353.32) | 2323 (1761-2925) | 8225.31 (6244.67-10383.14) | 0.5 (0.46 to 0.54) | < 0.001 |
| Haiti | 9459 (7054-12213) | 7294.82 (5472.72-9408.23) | 21207 (16032-27009) | 7645.75 (5776.07-9746.73) | 0.13 (0.09 to 0.17) | < 0.001 |
| Honduras | 6856 (5087-9088) | 8300.39 (6137.17-11022.98) | 23455 (17222-30526) | 8372.72 (6156.64-10930.01) | 0.01 (-0.12 to 0.13) | 0.908 |
| Hungary | 87896 (65617-110987) | 10269.46 (7659.73-12960.02) | 117239 (81469-158925) | 9489.22 (6645.79-12811.15) | -0.24 (-0.37 to -0.1) | < 0.001 |
| Iceland | 885 (670-1160) | 6097.13 (4616.25-8012.57) | 1719 (1254-2280) | 6141.47 (4488.07-8152.07) | 0.03 (-0.02 to 0.07) | 0.296 |
| India | 1998676 (1611821-2430474) | 11945.35 (9629.47-14560.31) | 7358870 (6005803-8957660) | 13813.93 (11261.51-16830.75) | 0.45 (0.42 to 0.49) | < 0.001 |
| Indonesia | 188204 (151346-230162) | 4940.83 (3968.59-6050.45) | 414279 (329600-498884) | 4208.45 (3349.54-5080.89) | -0.35 (-0.67 to -0.03) | 0.03 |
| Iran (Islamic Republic of) | 30204 (24318-37271) | 3223.27 (2599.68-3972.97) | 102190 (81340-125769) | 3277.39 (2606.8-4037.34) | 0.05 (0.03 to 0.07) | < 0.001 |
| Iraq | 10871 (8234-14124) | 3328.52 (2518.65-4326.56) | 31021 (23506-40429) | 3384.14 (2561.02-4419.23) | 0.04 (-0.08 to 0.16) | 0.481 |
| Ireland | 14642 (11330-18566) | 6416.55 (4968.63-8139.26) | 24086 (16752-33204) | 6155.38 (4289.22-8447.19) | -0.13 (-0.16 to -0.09) | < 0.001 |
| Israel | 15974 (12142-20710) | 6114.62 (4635.64-7957.93) | 44491 (30856-62924) | 6807.33 (4759.23-9550.51) | 0.33 (0.29 to 0.36) | < 0.001 |
| Italy | 292292 (230316-359569) | 5842.54 (4599.63-7194.25) | 367320 (288401-456891) | 4755.44 (3740.72-5904.49) | -0.67 (-0.77 to -0.57) | < 0.001 |
| Jamaica | 7146 (5085-9490) | 7800.71 (5582.61-10329.46) | 11524 (8876-15039) | 8267.04 (6351.46-10784.47) | 0.17 (0.11 to 0.23) | < 0.001 |
| Japan | 630726 (467030-813838) | 7038.93 (5192.36-9105.45) | 1804268 (1311116-2349820) | 8121.28 (6060.09-10411.84) | 0.43 (0.32 to 0.53) | < 0.001 |
| Jordan | 1439 (1096-1872) | 3121.6 (2385.03-4052.26) | 11937 (8154-20165) | 4638.19 (3168.14-7820.31) | 1.3 (1.21 to 1.39) | < 0.001 |
| Kazakhstan | 49554 (37532-63737) | 7534.35 (5706.4-9694.27) | 77999 (58560-99351) | 8287.37 (6236.82-10558.26) | 0.32 (0.28 to 0.36) | < 0.001 |
| Kenya | 17158 (13880-20848) | 5171.53 (4181.14-6296.59) | 56246 (45240-69246) | 5730.18 (4608.7-7060) | 0.34 (0.29 to 0.39) | < 0.001 |
| Kiribati | 55 (42-72) | 3362.24 (2564.91-4354.3) | 108 (80-141) | 3522.16 (2632.97-4620.96) | 0.15 (0.13 to 0.16) | < 0.001 |
| Kuwait | 520 (398-681) | 3670.42 (2808.8-4810.41) | 2937 (2229-3787) | 4263.29 (3242.03-5484.93) | 0.47 (0.43 to 0.52) | < 0.001 |
| Kyrgyzstan | 11791 (9096-15061) | 7654.38 (5893.49-9784) | 17440 (13737-21477) | 8146.44 (6419.69-10081.59) | 0.19 (0.18 to 0.21) | < 0.001 |
| Lao People's Democratic Republic | 4705 (3668-6028) | 5364.18 (4190.14-6847.36) | 10319 (7671-13406) | 5715.64 (4246.35-7426.1) | 0.21 (0.19 to 0.22) | < 0.001 |
| Latvia | 22612 (17117-29769) | 10390.02 (7853.64-13699.12) | 23027 (17994-28902) | 9458.76 (7340.12-11877.86) | -0.3 (-0.49 to -0.11) | 0.002 |
| Lebanon | 3101 (2385-3914) | 3443.61 (2655.49-4348.19) | 10426 (7962-13868) | 3581.61 (2736.25-4767.74) | 0.12 (0.11 to 0.13) | < 0.001 |
| Lesotho | 1798 (1369-2327) | 3854.32 (2935.43-4983.08) | 2437 (1828-3113) | 4195.95 (3159.69-5358.81) | 0.27 (0.25 to 0.3) | < 0.001 |
| Liberia | 2749 (2067-3710) | 5951.64 (4485.56-7993.63) | 4473 (3272-6183) | 6239.32 (4575.27-8604.5) | 0.14 (0.1 to 0.19) | < 0.001 |
| Libya | 2328 (1806-2946) | 3403.21 (2639.57-4311.79) | 6431 (4819-8315) | 3568.72 (2675.36-4616.16) | 0.16 (0.13 to 0.19) | < 0.001 |
| Lithuania | 25228 (19597-31900) | 9862.54 (7664.49-12478.76) | 33077 (25590-42045) | 9772.3 (7567.48-12404.95) | -0.05 (-0.13 to 0.04) | 0.302 |
| Luxembourg | 1851 (1370-2381) | 6025.76 (4490.29-7733) | 2824 (2033-3791) | 5772.29 (4154.87-7739.8) | -0.17 (-0.2 to -0.13) | < 0.001 |
| Madagascar | 9111 (7031-11722) | 4753.75 (3677.48-6102.58) | 19143 (14518-24521) | 4873.53 (3696.97-6233.04) | 0.08 (0.03 to 0.13) | 0.001 |
| Malawi | 8035 (6117-10434) | 4780.15 (3640.69-6225.56) | 16786 (12455-22615) | 5011.41 (3722.35-6725.82) | 0.16 (0.14 to 0.19) | < 0.001 |
| Malaysia | 21938 (17333-28469) | 6044.09 (4776.43-7835.2) | 77998 (59583-98384) | 6244.28 (4776.63-7876.69) | 0.11 (0.09 to 0.14) | < 0.001 |
| Maldives | 141 (110-176) | 5859.64 (4589.36-7352.1) | 592 (451-775) | 5544.42 (4215.87-7263.94) | -0.17 (-0.22 to -0.12) | < 0.001 |
| Mali | 9153 (6811-12179) | 5929.33 (4422.84-7843.42) | 19439 (14649-25908) | 5943.48 (4486.12-7890.44) | -0.02 (-0.09 to 0.06) | 0.653 |
| Malta | 963 (746-1257) | 4310.07 (3339.15-5619.99) | 2334 (1763-2981) | 4434.35 (3363.65-5657.11) | 0.08 (-0.08 to 0.25) | 0.324 |
| Marshall Islands | 24 (18-31) | 3456.78 (2633.17-4384.45) | 45 (34-58) | 3715.75 (2826.87-4849.79) | 0.19 (0.07 to 0.31) | 0.002 |
| Mauritania | 2809 (2104-3680) | 5971.8 (4487.4-7802.64) | 5430 (4036-7201) | 6241.02 (4643.28-8273.06) | 0.13 (0.12 to 0.15) | < 0.001 |
| Mauritius | 1959 (1481-2515) | 5664 (4268.08-7267.28) | 5250 (4066-6783) | 5511.05 (4269.14-7113.45) | -0.13 (-0.25 to 0) | 0.045 |
| Mexico | 168521 (136196-204090) | 9621.84 (7760.49-11671.14) | 767147 (629888-926988) | 13320.09 (10936.75-16100.27) | 1.07 (0.8 to 1.34) | < 0.001 |
| Micronesia (Federated States of) | 76 (58-101) | 3515.58 (2665.27-4672.88) | 105 (80-134) | 3736.28 (2846.26-4780.73) | 0.2 (0.16 to 0.23) | < 0.001 |
| Monaco | 242 (189-307) | 6147.93 (4801.48-7795.83) | 288 (209-393) | 6004.68 (4420.32-8137.34) | -0.08 (-0.1 to -0.05) | < 0.001 |
| Mongolia | 3796 (2912-4898) | 7493.09 (5749.97-9673.5) | 6566 (4956-8584) | 7066.02 (5330.95-9235.65) | -0.21 (-0.32 to -0.11) | < 0.001 |
| Montenegro | 3103 (2360-3972) | 10463.39 (7951.73-13380.44) | 5477 (4160-7249) | 10122.47 (7693.87-13395.13) | -0.1 (-0.13 to -0.08) | < 0.001 |
| Morocco | 18023 (13844-23192) | 3165.53 (2430.19-4076.39) | 46840 (35504-59389) | 3323.25 (2521.57-4211.86) | 0.17 (0.11 to 0.24) | < 0.001 |
| Mozambique | 11873 (9029-15370) | 4874.87 (3707.34-6318.01) | 24107 (18107-31650) | 5095.7 (3836.11-6695.58) | 0.2 (0.15 to 0.24) | < 0.001 |
| Myanmar | 54208 (41212-69776) | 5431.49 (4138.54-6970.86) | 138354 (106256-176693) | 5956.31 (4564.17-7602.5) | 0.31 (0.21 to 0.4) | < 0.001 |
| Namibia | 1258 (973-1664) | 4047.83 (3128.26-5336.04) | 2574 (1918-3370) | 3953.81 (2955.65-5162.85) | -0.07 (-0.11 to -0.03) | < 0.001 |
| Nauru | 5 (4-7) | 3603.74 (2768.13-4639.77) | 9 (7-12) | 3670.12 (2844.2-4734.65) | 0.06 (0.05 to 0.08) | < 0.001 |
| Nepal | 30363 (23116-39134) | 8876.26 (6755.16-11445.55) | 89605 (68900-115940) | 8597.68 (6607.38-11107.32) | -0.11 (-0.21 to -0.01) | 0.025 |
| Netherlands | 71369 (54631-97095) | 6368.06 (4872.73-8611.42) | 119875 (80686-173084) | 6553.5 (4430.47-9287.41) | 0.11 (0.06 to 0.17) | < 0.001 |
| New Zealand | 23171 (16883-30503) | 10640.08 (7732.6-14035.03) | 69950 (51377-90535) | 16009.17 (11754.29-20739.92) | 1.3 (1.22 to 1.38) | < 0.001 |
| Nicaragua | 5967 (4493-7588) | 9358.94 (7042.39-11902.55) | 20503 (14745-27267) | 9336.59 (6708.84-12423.88) | -0.06 (-0.15 to 0.04) | 0.24 |
| Niger | 5374 (4064-6917) | 5605.82 (4243.9-7218.49) | 18832 (14006-24670) | 5955.85 (4421.8-7816.97) | 0.19 (0.18 to 0.2) | < 0.001 |
| Nigeria | 122333 (98372-151631) | 6265.9 (5036.34-7772.11) | 229925 (184046-284203) | 6605.34 (5289.89-8176.76) | 0.15 (0.12 to 0.18) | < 0.001 |
| Niue | 5 (3-6) | 3805.85 (2866.84-4942.62) | 4 (3-5) | 3878.79 (2929.44-4996.56) | 0.06 (0.04 to 0.09) | < 0.001 |
| North Macedonia | 7523 (5711-9912) | 9602.41 (7287.38-12667.84) | 16503 (12244-21606) | 9769.56 (7264.34-12794.78) | 0.07 (-0.01 to 0.16) | 0.093 |
| Northern Mariana Islands | 17 (13-22) | 4169.85 (3201.04-5355.85) | 80 (60-104) | 4237.65 (3177.49-5515.45) | 0.07 (0.05 to 0.09) | < 0.001 |
| Norway | 30198 (23160-37551) | 7481.8 (5753.87-9299.46) | 38201 (31184-46154) | 7165.43 (5857.11-8646.98) | -0.1 (-0.27 to 0.08) | 0.275 |
| Oman | 740 (568-935) | 3214.88 (2466.78-4060.43) | 2387 (1803-3129) | 3870.01 (2925.21-5068.44) | 0.6 (0.53 to 0.67) | < 0.001 |
| Pakistan | 229428 (182700-288744) | 11454.63 (9101.39-14434.05) | 529785 (415564-666950) | 12386.7 (9725.62-15614.42) | 0.29 (0.15 to 0.44) | < 0.001 |
| Palau | 16 (12-21) | 3841.37 (2902.82-4977.47) | 33 (25-46) | 3801.65 (2833.17-5168.13) | -0.1 (-0.18 to -0.02) | 0.015 |
| Palestine | 1363 (1030-1790) | 3578.74 (2708.12-4697.27) | 3553 (2758-4515) | 3714.83 (2883.34-4725.66) | 0.15 (0.04 to 0.26) | 0.006 |
| Panama | 6112 (4609-7795) | 9829.18 (7413.78-12540.23) | 19025 (13478-24775) | 9428.5 (6677.37-12261.43) | -0.14 (-0.19 to -0.09) | < 0.001 |
| Papua New Guinea | 2055 (1524-2695) | 3336.09 (2482.86-4350.16) | 5258 (3978-6807) | 3471.56 (2620.45-4502.67) | 0.13 (0.11 to 0.15) | < 0.001 |
| Paraguay | 15279 (11411-20311) | 15640.26 (11683.24-20789.91) | 39830 (29620-52246) | 15369.59 (11426.48-20160.19) | -0.06 (-0.09 to -0.02) | 0.003 |
| Peru | 66209 (50754-84235) | 13502.83 (10347.57-17182.03) | 215524 (166478-276975) | 14457.68 (11171.49-18564.9) | 0.23 (0.17 to 0.29) | < 0.001 |
| Philippines | 81330 (64086-102266) | 6885.59 (5407.23-8687.9) | 282983 (227043-349964) | 7732.01 (6196.49-9574.64) | 0.38 (0.33 to 0.42) | < 0.001 |
| Poland | 228607 (184491-283166) | 9347.84 (7526.05-11577.83) | 434012 (350960-529604) | 10071.33 (8160.97-12290.43) | 0.25 (0.22 to 0.28) | < 0.001 |
| Portugal | 43648 (32267-55904) | 5559.28 (4110.4-7125.41) | 92482 (63170-127949) | 6699.71 (4669.23-9082.6) | 0.61 (0.57 to 0.65) | < 0.001 |
| Puerto Rico | 15782 (11749-20656) | 8604.17 (6408.61-11263.15) | 38576 (29447-50322) | 9712.01 (7429.94-12670.01) | 0.39 (0.35 to 0.44) | < 0.001 |
| Qatar | 72 (54-95) | 3785.71 (2839-4976.39) | 686 (496-915) | 4463.81 (3221.63-5963.09) | 0.52 (0.43 to 0.61) | < 0.001 |
| Republic of Korea | 89593 (65418-112987) | 6807.96 (4959.25-8603.92) | 332553 (232454-447773) | 6844.68 (4780.7-9212.77) | 0.02 (-0.02 to 0.05) | 0.404 |
| Republic of Moldova | 22123 (17044-28270) | 9167.01 (7026.48-11719.59) | 33760 (25702-42523) | 9792.79 (7465.03-12318.06) | 0.21 (0.2 to 0.22) | < 0.001 |
| Romania | 146316 (112167-192987) | 10341.46 (7945.91-13594.72) | 233705 (170472-307550) | 10311.6 (7507.82-13558.91) | -0.02 (-0.07 to 0.03) | 0.437 |
| Russian Federation | 999169 (802226-1219812) | 9183.76 (7366.06-11216.68) | 1506223 (1235186-1770812) | 10078.8 (8282.7-11837.3) | 0.29 (0.22 to 0.37) | < 0.001 |
| Rwanda | 5996 (4639-7634) | 4872 (3765.18-6213.78) | 14606 (11177-18970) | 5041.79 (3864.44-6533.13) | 0.12 (0.11 to 0.13) | < 0.001 |
| Saint Kitts and Nevis | 176 (131-229) | 7733.36 (5791.08-10085.63) | 263 (198-334) | 8447.8 (6360.76-10701.16) | 0.28 (0.26 to 0.31) | < 0.001 |
| Saint Lucia | 358 (255-479) | 7669.67 (5442.64-10259.04) | 884 (644-1167) | 8036.07 (5852.34-10617.59) | 0.15 (0.12 to 0.18) | < 0.001 |
| Saint Vincent and the Grenadines | 317 (238-410) | 8189.49 (6166.26-10580.68) | 560 (427-717) | 8895.08 (6781.39-11395.24) | 0.27 (0.25 to 0.3) | < 0.001 |
| Samoa | 129 (96-168) | 3686.32 (2751.14-4823.74) | 227 (176-293) | 3838.99 (2968.21-4948.62) | 0.17 (0.11 to 0.23) | < 0.001 |
| San Marino | 118 (92-152) | 6380.48 (5008.78-8256.54) | 197 (148-262) | 5808.08 (4357.14-7740.91) | -0.31 (-0.35 to -0.26) | < 0.001 |
| Sao Tome and Principe | 181 (131-245) | 6059.73 (4385.17-8192.82) | 295 (218-405) | 6778.14 (5003.61-9289.49) | 0.36 (0.33 to 0.38) | < 0.001 |
| Saudi Arabia | 6368 (4775-8408) | 3599.92 (2700-4754.45) | 16151 (11843-21627) | 4001.24 (2923.03-5427.02) | 0.33 (0.29 to 0.38) | < 0.001 |
| Senegal | 7468 (5690-9654) | 5872.61 (4484.23-7588.34) | 19877 (14698-26257) | 6244.82 (4623.58-8247.33) | 0.19 (0.18 to 0.21) | < 0.001 |
| Serbia | 43157 (32877-55924) | 8500.5 (6443.13-11001.97) | 77076 (56519-101671) | 8211.33 (6008.92-10843.84) | -0.12 (-0.17 to -0.07) | < 0.001 |
| Seychelles | 182 (141-236) | 6044.93 (4671.83-7819.44) | 325 (250-434) | 6535.34 (5018.74-8735.82) | 0.24 (0.21 to 0.28) | < 0.001 |
| Sierra Leone | 4860 (3699-6323) | 5762.3 (4380.96-7493.96) | 8865 (6592-12052) | 6113.56 (4537.71-8307.04) | 0.18 (0.15 to 0.22) | < 0.001 |
| Singapore | 7370 (5592-10621) | 8043.93 (6119.29-11526) | 36299 (26776-49453) | 8992.65 (6607.57-12284.32) | 0.39 (0.34 to 0.44) | < 0.001 |
| Slovakia | 36624 (27534-47976) | 11031.78 (8290.34-14428.37) | 62523 (45720-83815) | 11122.86 (8133.87-14910.83) | 0.01 (-0.07 to 0.1) | 0.726 |
| Slovenia | 15509 (12081-19429) | 11031.48 (8597.67-13820.59) | 25672 (18621-33718) | 10300.87 (7452.49-13540.2) | -0.23 (-0.28 to -0.17) | < 0.001 |
| Solomon Islands | 152 (116-194) | 3799.17 (2899.23-4862.01) | 475 (365-609) | 4023.68 (3089.91-5158.78) | 0.19 (0.17 to 0.21) | < 0.001 |
| Somalia | 3508 (2726-4419) | 4646.4 (3607.3-5880.81) | 12653 (9458-16626) | 4837.09 (3603.72-6380.72) | 0.15 (0.06 to 0.24) | 0.001 |
| South Africa | 37317 (29427-46952) | 3755.2 (2958.54-4722.32) | 83212 (66850-103806) | 3612.97 (2900.86-4506.41) | -0.15 (-0.36 to 0.06) | 0.16 |
| South Sudan | 4283 (3248-5677) | 4827.74 (3662.82-6410.18) | 5939 (4468-7568) | 4937.62 (3720.55-6305.12) | 0.06 (0.03 to 0.1) | < 0.001 |
| Spain | 190706 (138299-249798) | 6216.63 (4496.33-8154.85) | 347531 (234484-487754) | 6820.06 (4731.6-9302.3) | 0.3 (0.28 to 0.32) | < 0.001 |
| Sri Lanka | 24571 (18541-31515) | 5774.74 (4359.95-7403.83) | 101822 (78271-130525) | 7028.52 (5413.83-9002.91) | 0.63 (0.61 to 0.65) | < 0.001 |
| Sudan | 11131 (8409-14409) | 3094.61 (2344.47-3999.9) | 20504 (15163-26615) | 3251.63 (2407.72-4220.67) | 0.18 (0.07 to 0.29) | 0.001 |
| Suriname | 856 (652-1088) | 8295.55 (6315.32-10551.03) | 2540 (1945-3339) | 8675.31 (6645.77-11404.78) | 0.14 (0.11 to 0.18) | < 0.001 |
| Sweden | 60854 (45400-77770) | 7161.58 (5346.8-9137.64) | 79853 (58476-106866) | 7268.03 (5293.95-9715.07) | 0.03 (-0.03 to 0.1) | 0.358 |
| Switzerland | 31608 (24645-41094) | 5544.03 (4307.18-7233.93) | 49709 (35753-65949) | 5584.24 (4031.6-7408.58) | 0.05 (0.01 to 0.09) | 0.027 |
| Syrian Arab Republic | 6348 (4823-8122) | 3520.51 (2679.1-4497.1) | 19850 (14831-26136) | 3856.29 (2885.7-5097.59) | 0.31 (0.25 to 0.36) | < 0.001 |
| Taiwan (Province of China) | 16138 (12108-21175) | 2619.24 (1965.79-3448.2) | 62358 (47876-79557) | 2858.66 (2194.26-3648.32) | 0.29 (0.24 to 0.34) | < 0.001 |
| Tajikistan | 9268 (7143-11954) | 7337.59 (5654.99-9472.28) | 15980 (12206-20642) | 7810.01 (5972.69-10082.99) | 0.22 (0.18 to 0.25) | < 0.001 |
| Thailand | 91462 (70499-116816) | 6402.83 (4937.72-8171.38) | 350707 (272162-450797) | 6558.95 (5088.34-8433.17) | 0.07 (0.04 to 0.1) | < 0.001 |
| Timor-Leste | 456 (355-574) | 5425.17 (4232.06-6840.26) | 2338 (1790-2954) | 5692.19 (4361.86-7186.41) | 0.18 (0.14 to 0.21) | < 0.001 |
| Togo | 2821 (2109-3684) | 5934.51 (4444.21-7734.59) | 10381 (7604-14414) | 6310.58 (4630.59-8736.08) | 0.13 (0.07 to 0.18) | < 0.001 |
| Tokelau | 2 (2-3) | 3643.34 (2827.33-4658.79) | 3 (2-4) | 4013.14 (2954.82-5443.89) | 0.32 (0.24 to 0.39) | < 0.001 |
| Tonga | 83 (64-109) | 3684.29 (2812.59-4812.8) | 137 (105-177) | 3754.13 (2878.95-4828.46) | 0.01 (-0.04 to 0.07) | 0.626 |
| Trinidad and Tobago | 3129 (2268-4095) | 7744.31 (5608.04-10148) | 7820 (5636-10151) | 8080.16 (5820.87-10491.76) | 0.1 (0.01 to 0.19) | 0.024 |
| Tunisia | 6813 (5212-8742) | 3500.7 (2682.56-4489.7) | 21772 (16129-28387) | 3647 (2700.9-4754.95) | 0.14 (0.1 to 0.18) | < 0.001 |
| Turkey | 47374 (35877-61286) | 3399.28 (2580.07-4395.91) | 169664 (132360-219546) | 3727.22 (2908.09-4821.15) | 0.3 (0.25 to 0.36) | < 0.001 |
| Turkmenistan | 6359 (4640-8360) | 6897.4 (5021.84-9080.5) | 13836 (10459-17953) | 7831.7 (5903.13-10181.88) | 0.41 (0.39 to 0.43) | < 0.001 |
| Tuvalu | 11 (9-15) | 3528.23 (2706.21-4580.58) | 19 (14-24) | 3712.86 (2802.66-4773.44) | 0.16 (0.15 to 0.17) | < 0.001 |
| Uganda | 12907 (9967-16136) | 4866.97 (3753.13-6088.6) | 31884 (24253-41598) | 5105.55 (3881.09-6659.88) | 0.17 (0.12 to 0.22) | < 0.001 |
| Ukraine | 385539 (298718-484322) | 8714.48 (6739.55-10955.06) | 395245 (287415-527900) | 8246.33 (6004.23-10992.19) | -0.23 (-0.42 to -0.04) | 0.017 |
| United Arab Emirates | 323 (247-419) | 3164.62 (2420.68-4103.07) | 1420 (1086-1829) | 3297.34 (2499.21-4298.82) | 0.14 (0.1 to 0.19) | < 0.001 |
| United Kingdom | 300475 (243061-368121) | 5737.96 (4643.54-7025.68) | 249315 (200199-309251) | 3926.78 (3171.41-4843.91) | -1.18 (-1.33 to -1.02) | < 0.001 |
| United Republic of Tanzania | 22393 (17172-28782) | 4982.17 (3820.15-6408.32) | 52925 (39158-70442) | 5295.07 (3918.5-7041.82) | 0.19 (0.15 to 0.23) | < 0.001 |
| United States of America | 4005345 (3182866-4972450) | 20980.18 (16678.86-26027.05) | 7507566 (5989760-9290531) | 23418.33 (18733.85-28939.68) | 0.39 (0.11 to 0.68) | 0.007 |
| United States Virgin Islands | 329 (250-434) | 8939.6 (6805.68-11785.71) | 954 (731-1241) | 9321.79 (7153.55-12086.46) | 0.15 (0.08 to 0.22) | < 0.001 |
| Uruguay | 22530 (15972-30013) | 10466.65 (7428.32-13953.26) | 42332 (32292-54742) | 13464.73 (10282.03-17425.27) | 0.89 (0.79 to 0.99) | < 0.001 |
| Uzbekistan | 38969 (30214-48933) | 7056.51 (5468.67-8863.4) | 90764 (65223-121933) | 8283.48 (5942.52-11096.34) | 0.5 (0.41 to 0.58) | < 0.001 |
| Vanuatu | 73 (55-97) | 3487.68 (2636.04-4651.47) | 244 (186-317) | 3685.71 (2807.13-4784.07) | 0.16 (0.1 to 0.23) | < 0.001 |
| Venezuela (Bolivarian Republic of) | 37466 (27879-48176) | 9268.92 (6898.15-11913.56) | 127669 (93194-168833) | 8787.99 (6405.06-11631.36) | -0.2 (-0.27 to -0.14) | < 0.001 |
| Viet Nam | 132265 (102433-167886) | 6396.18 (4955.85-8120.26) | 260134 (179686-351087) | 5516.37 (3808.08-7451.69) | -0.47 (-0.51 to -0.43) | < 0.001 |
| Yemen | 6197 (4667-8097) | 3077.29 (2325-4008.77) | 17114 (12951-22255) | 3177.74 (2398.01-4141.65) | 0.09 (0.08 to 0.11) | < 0.001 |
| Zambia | 4647 (3515-6081) | 4934.36 (3741.48-6462.94) | 13793 (10586-18116) | 5191.06 (3981.19-6825.42) | 0.17 (0.11 to 0.22) | < 0.001 |
| Zimbabwe | 6866 (5001-9109) | 3911.86 (2860.85-5167.89) | 13124 (9391-17830) | 3995.93 (2870.07-5394.1) | 0.03 (-0.05 to 0.11) | 0.424 |

Abbreviation: AAPC, average annual percentage change.

**Table S6. The case number and age-standardized rate of mortality of urinary tract infections in 1990 and 2021, and the temporal trends between 1990 and 2021 at the national level.**

| Location | Number of cases, 1990 | Age-standardized rate per 100 000 population, 1990 | Number of cases, 2021 | Age-standardized rate per 100 000 population, 2021 | AAPC, 1990-2021 | p value |
| --- | --- | --- | --- | --- | --- | --- |
| Afghanistan | 55 (28-136) | 28.88 (14.59-72.76) | 97 (51-208) | 34.52 (18.01-73.01) | 0.57 (0.48 to 0.66) | < 0.001 |
| Albania | 4 (2-6) | 4.48 (2.54-7.31) | 6 (3-11) | 3.04 (1.46-5.5) | -1.37 (-1.74 to -0.99) | < 0.001 |
| Algeria | 55 (28-148) | 25.58 (13.4-63.47) | 276 (167-455) | 34.45 (21.08-56.3) | 1.08 (0.76 to 1.39) | < 0.001 |
| American Samoa | 0 (0-1) | 66.53 (40.85-107.72) | 1 (1-2) | 68.24 (41.46-105.03) | 0.24 (-0.51 to 1.01) | 0.529 |
| Andorra | 1 (0-1) | 31.29 (16.42-64.03) | 2 (1-3) | 23.12 (12.91-36.47) | -1.09 (-1.42 to -0.74) | < 0.001 |
| Angola | 12 (6-29) | 12.75 (5.68-31.74) | 49 (23-109) | 15.18 (6.99-34.14) | 0.6 (0.42 to 0.78) | < 0.001 |
| Antigua and Barbuda | 0 (0-0) | 9.85 (8.01-11.88) | 2 (1-2) | 42.17 (34.66-50.59) | 4.97 (3.8 to 6.15) | < 0.001 |
| Argentina | 149 (122-177) | 9.92 (8.12-11.81) | 2976 (2369-3575) | 88.18 (70.41-105.87) | 7.1 (5.84 to 8.38) | < 0.001 |
| Armenia | 11 (8-14) | 9.51 (7.28-11.86) | 42 (33-53) | 18.08 (14.09-22.92) | 2.2 (0.32 to 4.11) | 0.021 |
| Australia | 174 (142-204) | 16.92 (13.72-19.86) | 813 (598-995) | 26.51 (19.66-32.44) | 1.67 (0.17 to 3.19) | 0.029 |
| Austria | 120 (98-142) | 15.25 (12.41-18.06) | 215 (160-265) | 15.33 (11.47-18.92) | 0.13 (-1.23 to 1.5) | 0.856 |
| Azerbaijan | 20 (13-31) | 8.94 (5.74-13.78) | 60 (30-91) | 15.77 (7.9-24.24) | 1.84 (1.51 to 2.17) | < 0.001 |
| Bahamas | 1 (1-1) | 15.99 (12.8-19.44) | 10 (8-13) | 63.06 (48.93-79.58) | 4.49 (3.14 to 5.87) | < 0.001 |
| Bahrain | 0 (0-1) | 8.36 (2.28-21.13) | 2 (1-5) | 14.2 (4.2-33.71) | 1.69 (0.44 to 2.95) | 0.008 |
| Bangladesh | 462 (263-874) | 35.29 (19.88-69.16) | 1523 (906-2429) | 33.43 (19.91-53.32) | -0.02 (-0.71 to 0.67) | 0.95 |
| Barbados | 4 (3-5) | 23.77 (19.64-28.4) | 24 (19-30) | 89.91 (69.93-112.31) | 4.47 (3.42 to 5.52) | < 0.001 |
| Belarus | 95 (78-114) | 12.68 (10.41-15.18) | 222 (171-280) | 21.5 (16.54-27.18) | 1.38 (-1 to 3.82) | 0.258 |
| Belgium | 91 (73-108) | 9.6 (7.64-11.47) | 716 (514-891) | 38.42 (28.17-47.59) | 4.47 (3.81 to 5.14) | < 0.001 |
| Belize | 0 (0-1) | 10.98 (8.87-13.3) | 4 (3-5) | 42.09 (33.13-51.38) | 4.53 (3.53 to 5.54) | < 0.001 |
| Benin | 11 (6-19) | 15.71 (7.9-26.75) | 25 (12-48) | 14.5 (6.56-27.58) | -0.25 (-0.34 to -0.15) | < 0.001 |
| Bermuda | 0 (0-0) | 8.1 (6.58-9.79) | 2 (1-2) | 17.96 (13.18-23.54) | 2.63 (1.61 to 3.65) | < 0.001 |
| Bhutan | 3 (2-6) | 45.28 (21.22-84.63) | 16 (8-27) | 68.51 (32.53-115.09) | 1.36 (1.23 to 1.48) | < 0.001 |
| Bolivia (Plurinational State of) | 37 (22-62) | 34.65 (20.37-59.78) | 170 (97-282) | 52.1 (30.03-86.17) | 1.31 (1.05 to 1.58) | < 0.001 |
| Bosnia and Herzegovina | 26 (15-39) | 15.84 (9.06-24.36) | 31 (16-61) | 8.95 (4.66-17.54) | -1.73 (-2.24 to -1.22) | < 0.001 |
| Botswana | 1 (0-3) | 8.56 (2.07-18.42) | 6 (2-12) | 11.49 (3.3-23.8) | 0.96 (0.09 to 1.83) | 0.031 |
| Brazil | 909 (801-993) | 28.9 (25.11-31.74) | 14166 (11351-16060) | 112.45 (90.48-127.28) | 4.56 (3.81 to 5.3) | < 0.001 |
| Brunei Darussalam | 2 (1-3) | 62.38 (30.8-112.29) | 7 (4-11) | 76.57 (41.14-120.28) | 0.56 (0.23 to 0.9) | 0.001 |
| Bulgaria | 208 (177-243) | 41.16 (34.73-48.87) | 136 (108-169) | 16.62 (13.28-20.59) | -2.49 (-3.72 to -1.25) | < 0.001 |
| Burkina Faso | 23 (11-40) | 17.06 (8.48-29.93) | 44 (19-85) | 14.57 (6.28-28.2) | -0.46 (-0.66 to -0.25) | < 0.001 |
| Burundi | 40 (23-66) | 44.76 (25.69-75.49) | 59 (32-99) | 43.45 (24.04-74.42) | -0.12 (-0.29 to 0.06) | 0.187 |
| Cabo Verde | 1 (0-1) | 5.14 (2.22-9.3) | 2 (1-3) | 7.62 (3.32-14.25) | 1.3 (1.13 to 1.46) | < 0.001 |
| Cambodia | 39 (20-71) | 24.7 (12.91-45.46) | 134 (79-210) | 28.98 (17.04-45.98) | 0.53 (0.48 to 0.59) | < 0.001 |
| Cameroon | 36 (18-64) | 27.68 (13.3-47.88) | 71 (31-138) | 19.21 (8.18-37.7) | -1.19 (-1.3 to -1.09) | < 0.001 |
| Canada | 313 (253-372) | 17.39 (14.01-20.65) | 1269 (974-1526) | 26.3 (20.36-31.62) | 1.55 (1.09 to 2.01) | < 0.001 |
| Central African Republic | 5 (2-12) | 16.78 (7.49-42.43) | 9 (4-22) | 16.37 (7.06-41.08) | -0.08 (-0.18 to 0.01) | 0.088 |
| Chad | 12 (5-22) | 11.35 (5.14-20.93) | 15 (7-32) | 10.42 (4.39-21.53) | -0.27 (-0.32 to -0.22) | < 0.001 |
| Chile | 237 (198-279) | 57.31 (47.46-67.33) | 1071 (850-1285) | 76.17 (60.7-91.21) | 0.85 (-0.53 to 2.24) | 0.229 |
| China | 1576 (954-2197) | 6.07 (3.57-8.5) | 2598 (1710-4573) | 2.83 (1.86-4.97) | -2.41 (-2.82 to -2.01) | < 0.001 |
| Colombia | 140 (117-164) | 22.01 (18.23-25.8) | 1090 (813-1342) | 37.34 (28.17-45.81) | 1.66 (0.28 to 3.05) | 0.018 |
| Comoros | 3 (2-5) | 54.37 (31.14-87.97) | 12 (7-19) | 64.13 (37.11-101.06) | 0.54 (0.43 to 0.66) | < 0.001 |
| Congo | 7 (4-15) | 22.58 (11.11-46.89) | 16 (8-29) | 22.63 (11.39-41.34) | 0.02 (-0.17 to 0.22) | 0.816 |
| Cook Islands | 0 (0-0) | 2.65 (1.57-4.39) | 0 (0-0) | 1.89 (1.11-3.04) | -1.04 (-1.59 to -0.47) | < 0.001 |
| Costa Rica | 10 (8-12) | 13.36 (10.92-16.06) | 104 (80-127) | 36.91 (28.73-44.92) | 3.24 (2.15 to 4.34) | < 0.001 |
| Coted'Ivoire | 13 (7-23) | 15.93 (7.94-26.87) | 42 (19-77) | 14.58 (6.74-26.87) | -0.29 (-0.49 to -0.09) | 0.005 |
| Croatia | 106 (89-125) | 35.94 (30.07-42.31) | 341 (277-416) | 56.47 (45.8-69.32) | 1.44 (-0.35 to 3.26) | 0.116 |
| Cuba | 20 (16-24) | 4.8 (3.94-5.69) | 149 (118-183) | 13.61 (10.82-16.78) | 3.41 (1.91 to 4.92) | < 0.001 |
| Cyprus | 35 (20-59) | 142.85 (79.8-244.61) | 68 (41-101) | 85.46 (50.55-128.25) | -1.57 (-2.45 to -0.68) | 0.001 |
| Czechia | 366 (309-427) | 45.03 (37.96-52.56) | 397 (312-490) | 28.74 (22.61-35.54) | -1.35 (-2.82 to 0.14) | 0.076 |
| Democratic People's Republic of Korea | 49 (22-111) | 7.72 (3.44-18.3) | 97 (42-232) | 5.87 (2.52-14.21) | -0.89 (-0.94 to -0.83) | < 0.001 |
| Democratic Republic of the Congo | 59 (27-134) | 15.02 (6.84-37.02) | 168 (73-382) | 15.14 (6.56-34.43) | 0 (-0.05 to 0.06) | 0.902 |
| Denmark | 89 (73-105) | 17.27 (14.19-20.27) | 336 (258-412) | 40.76 (31.47-49.91) | 2.83 (1.27 to 4.41) | < 0.001 |
| Djibouti | 1 (1-2) | 36.89 (20.04-64.81) | 8 (5-14) | 55.55 (31.32-89.64) | 1.33 (1.27 to 1.39) | < 0.001 |
| Dominica | 0 (0-0) | 5.43 (3.39-8.32) | 1 (0-1) | 12.66 (6.88-20.87) | 2.77 (2.58 to 2.97) | < 0.001 |
| Dominican Republic | 4 (2-6) | 3.31 (2.02-5.68) | 17 (9-28) | 3.67 (2.02-6.15) | 0.37 (-0.47 to 1.23) | 0.387 |
| Ecuador | 35 (28-43) | 19.48 (15.82-23.7) | 253 (190-335) | 35.5 (26.99-46.75) | 2.03 (0.54 to 3.55) | 0.008 |
| Egypt | 16 (7-40) | 3.13 (1.35-7.96) | 116 (57-184) | 12.32 (6.05-19.45) | 4.52 (3.93 to 5.11) | < 0.001 |
| El Salvador | 24 (16-38) | 18.27 (11.72-28.55) | 108 (63-170) | 29.77 (17.53-46.67) | 1.56 (1.24 to 1.88) | < 0.001 |
| Equatorial Guinea | 1 (0-2) | 15.03 (6.7-34.6) | 4 (2-7) | 24.04 (11.57-45.04) | 1.57 (1.41 to 1.73) | < 0.001 |
| Eritrea | 11 (6-19) | 35.35 (18.54-61.33) | 43 (24-75) | 48.62 (27.11-85.2) | 1.03 (0.97 to 1.08) | < 0.001 |
| Estonia | 44 (36-52) | 34.6 (28.87-41.03) | 58 (45-72) | 28.24 (22.05-35.15) | -0.7 (-2.08 to 0.7) | 0.325 |
| Eswatini | 1 (0-3) | 14.38 (3.9-28.52) | 3 (1-6) | 14.36 (3.85-28.33) | 0.05 (-0.18 to 0.28) | 0.659 |
| Ethiopia | 626 (422-933) | 115.35 (76.38-175.53) | 1383 (955-1906) | 98.45 (67.38-136.06) | -0.53 (-0.82 to -0.24) | < 0.001 |
| Fiji | 1 (1-2) | 10.45 (6-17.46) | 4 (2-6) | 17.93 (10.78-28.73) | 1.86 (1.32 to 2.39) | < 0.001 |
| Finland | 169 (136-198) | 37.29 (29.92-43.77) | 140 (102-176) | 14.23 (10.59-17.77) | -2.89 (-3.64 to -2.14) | < 0.001 |
| France | 1011 (822-1177) | 17.93 (14.58-20.85) | 2384 (1787-2911) | 18.3 (13.8-22.45) | 0.07 (-0.7 to 0.84) | 0.864 |
| Gabon | 5 (2-10) | 21.17 (9.99-46.05) | 11 (5-21) | 32.12 (15.88-61.37) | 1.4 (1.14 to 1.66) | < 0.001 |
| Gambia | 2 (1-3) | 14.8 (7.62-24.63) | 5 (3-10) | 15.85 (7.26-30.49) | 0.22 (-0.18 to 0.63) | 0.281 |
| Georgia | 9 (7-11) | 2.65 (2.18-3.18) | 34 (26-45) | 9.1 (7.06-11.94) | 4.35 (0.93 to 7.88) | 0.012 |
| Germany | 730 (594-860) | 8.66 (7.02-10.2) | 3395 (2554-4162) | 23.91 (18.19-29.26) | 3.42 (2.15 to 4.69) | < 0.001 |
| Ghana | 20 (11-35) | 10.82 (5.89-19.13) | 181 (86-315) | 33.01 (15.86-57.25) | 3.68 (3.48 to 3.87) | < 0.001 |
| Greece | 143 (118-170) | 20.19 (16.57-23.97) | 685 (541-820) | 37.01 (29.35-44.24) | 1.99 (-0.23 to 4.26) | 0.079 |
| Greenland | 1 (0-1) | 62.15 (35.61-99.23) | 1 (0-2) | 47.32 (21.92-76.34) | -0.86 (-1.48 to -0.24) | 0.007 |
| Grenada | 1 (0-1) | 10.11 (8.2-12.14) | 2 (1-2) | 35.91 (28.1-44.28) | 4.34 (3.21 to 5.48) | < 0.001 |
| Guam | 1 (1-2) | 80.92 (48.63-123.74) | 2 (1-3) | 15.41 (9.37-23.23) | -5.59 (-8.05 to -3.08) | < 0.001 |
| Guatemala | 11 (9-14) | 12.7 (10.56-15.18) | 108 (86-132) | 24.96 (19.84-30.54) | 2.16 (0.37 to 3.98) | 0.018 |
| Guinea | 18 (9-32) | 15.55 (7.47-27.59) | 25 (12-49) | 14.03 (6.3-26.78) | -0.35 (-0.4 to -0.29) | < 0.001 |
| Guinea-Bissau | 2 (1-3) | 17.84 (8.89-30.67) | 3 (1-6) | 15.35 (6.86-30.58) | -0.49 (-0.56 to -0.41) | < 0.001 |
| Guyana | 1 (1-2) | 10.48 (8.3-13.01) | 8 (6-10) | 34.4 (24.21-45.2) | 4.01 (2.87 to 5.15) | < 0.001 |
| Haiti | 16 (9-34) | 16.54 (8.91-36.95) | 43 (21-86) | 21.25 (10.64-43.06) | 0.82 (0.76 to 0.89) | < 0.001 |
| Honduras | 19 (12-31) | 26.91 (16.82-43.7) | 125 (61-202) | 54.83 (26.8-88.24) | 2.34 (1.74 to 2.95) | < 0.001 |
| Hungary | 220 (184-257) | 27.5 (22.97-32.19) | 251 (197-308) | 18.71 (14.73-23) | -0.72 (-2 to 0.58) | 0.278 |
| Iceland | 1 (1-2) | 7.43 (5.4-9.57) | 9 (6-11) | 22.55 (16.47-28.19) | 3.91 (3.47 to 4.36) | < 0.001 |
| India | 4023 (2921-5380) | 29.66 (21.56-40.27) | 19207 (13841-24705) | 42 (30.33-54.23) | 1.3 (0.42 to 2.2) | 0.004 |
| Indonesia | 324 (219-489) | 10.61 (7.12-16.18) | 1207 (850-1706) | 16.68 (11.79-24.35) | 1.51 (1.39 to 1.63) | < 0.001 |
| Iran (Islamic Republic of) | 106 (75-189) | 17.55 (12.26-31.35) | 454 (322-756) | 17.78 (12.55-29.53) | 0.03 (-0.17 to 0.23) | 0.752 |
| Iraq | 18 (9-36) | 5.48 (2.81-11.06) | 45 (25-70) | 6.35 (3.47-9.99) | 0.57 (0.29 to 0.86) | < 0.001 |
| Ireland | 59 (49-70) | 28.38 (23.43-33.49) | 151 (112-190) | 32.36 (24.1-40.6) | 0.64 (-0.92 to 2.23) | 0.424 |
| Israel | 41 (34-49) | 20.5 (16.64-24.52) | 440 (324-542) | 56.42 (41.96-69.63) | 3.25 (1.85 to 4.67) | < 0.001 |
| Italy | 223 (187-246) | 4.49 (3.74-4.97) | 1302 (935-1575) | 10.66 (7.81-12.97) | 2.88 (1.02 to 4.77) | 0.002 |
| Jamaica | 8 (6-9) | 8.01 (6.54-9.51) | 49 (36-64) | 27.65 (20.43-35.58) | 4.07 (2.75 to 5.4) | < 0.001 |
| Japan | 882 (732-965) | 10.73 (8.77-11.82) | 6066 (4065-7312) | 15.04 (10.52-17.91) | 1.06 (0.33 to 1.8) | 0.004 |
| Jordan | 4 (2-7) | 11.42 (6.65-20.62) | 24 (15-37) | 14.52 (8.71-22.6) | 0.85 (-0.77 to 2.49) | 0.307 |
| Kazakhstan | 69 (57-80) | 10.58 (8.75-12.37) | 169 (117-234) | 20.24 (13.89-28.36) | 2.28 (0.77 to 3.82) | 0.003 |
| Kenya | 113 (84-169) | 41.44 (30.35-62.22) | 496 (336-713) | 63.74 (43.14-91.43) | 1.4 (1.23 to 1.56) | < 0.001 |
| Kiribati | 0 (0-0) | 19.82 (11.05-35.84) | 0 (0-1) | 24.91 (14.37-41.07) | 0.76 (0.57 to 0.95) | < 0.001 |
| Kuwait | 0 (0-0) | 1.86 (1.38-2.32) | 17 (11-22) | 23.84 (15.58-31.96) | 8.08 (3.21 to 13.18) | 0.001 |
| Kyrgyzstan | 21 (18-25) | 14.05 (11.64-16.51) | 28 (21-37) | 14.41 (10.77-18.85) | 0.04 (-3 to 3.17) | 0.98 |
| Lao People's Democratic Republic | 18 (9-35) | 27.25 (13.7-54.87) | 39 (21-65) | 26.22 (13.99-44.12) | -0.14 (-0.18 to -0.09) | < 0.001 |
| Latvia | 66 (54-79) | 29.84 (24.29-35.84) | 90 (67-120) | 30.24 (22.49-40.36) | 0.04 (-1.16 to 1.26) | 0.947 |
| Lebanon | 44 (26-69) | 58.52 (34.82-92.52) | 177 (110-277) | 52.77 (32.87-82.23) | -0.32 (-0.58 to -0.05) | 0.021 |
| Lesotho | 3 (1-7) | 8.74 (2.45-17.79) | 7 (2-14) | 16.82 (4.63-33.34) | 2.21 (1.75 to 2.68) | < 0.001 |
| Liberia | 7 (4-12) | 21.05 (10.65-35.4) | 10 (4-19) | 17.95 (7.71-33.04) | -0.55 (-0.75 to -0.35) | < 0.001 |
| Libya | 10 (5-25) | 15.43 (7.29-37.2) | 41 (23-67) | 25.15 (13.91-40.93) | 1.66 (1.14 to 2.18) | < 0.001 |
| Lithuania | 45 (36-53) | 17.52 (14.3-20.83) | 90 (70-116) | 21.79 (16.87-28.34) | 0.74 (-0.34 to 1.84) | 0.181 |
| Luxembourg | 2 (1-2) | 5.37 (4.36-6.48) | 9 (7-11) | 11.93 (9.16-14.8) | 2.75 (1.66 to 3.85) | < 0.001 |
| Madagascar | 52 (31-88) | 32.84 (19.27-57.1) | 100 (55-161) | 33.56 (18.34-53.78) | 0.05 (-0.17 to 0.26) | 0.673 |
| Malawi | 50 (29-81) | 38.28 (22.19-64.11) | 122 (73-197) | 44.32 (26.21-70.96) | 0.54 (0.34 to 0.74) | < 0.001 |
| Malaysia | 61 (20-125) | 17.45 (5.57-35.69) | 244 (87-542) | 26.93 (9.69-60.14) | 1.4 (0.92 to 1.88) | < 0.001 |
| Maldives | 1 (0-1) | 34.63 (19.99-66.99) | 3 (2-4) | 28.67 (17.09-44.14) | -0.59 (-0.91 to -0.26) | 0.001 |
| Mali | 19 (9-34) | 17.16 (8.22-30.59) | 36 (16-71) | 14.93 (6.41-29.48) | -0.44 (-0.54 to -0.33) | < 0.001 |
| Malta | 3 (3-4) | 16.39 (13.21-19.78) | 20 (15-26) | 31.01 (22.76-39.28) | 2.18 (1.8 to 2.56) | < 0.001 |
| Marshall Islands | 0 (0-0) | 20.45 (10.71-43.05) | 0 (0-0) | 23.75 (13.38-44.78) | 0.47 (0.41 to 0.53) | < 0.001 |
| Mauritania | 9 (5-15) | 23.33 (11.93-38.74) | 13 (6-25) | 19.65 (8.61-37.04) | -0.56 (-0.73 to -0.4) | < 0.001 |
| Mauritius | 2 (1-2) | 6.37 (5.19-7.59) | 21 (17-26) | 23.68 (18.92-28.91) | 5.76 (5.11 to 6.42) | < 0.001 |
| Mexico | 475 (434-526) | 36.66 (33.29-40.67) | 3529 (2979-4202) | 65.47 (55.23-77.92) | 1.92 (1.29 to 2.55) | < 0.001 |
| Micronesia (Federated States of) | 1 (0-1) | 35.36 (18-94.01) | 1 (0-1) | 35.94 (20.42-68.52) | 0.05 (-0.03 to 0.13) | 0.241 |
| Monaco | 0 (0-0) | 1.73 (0.93-2.96) | 0 (0-0) | 3.15 (1.78-5.26) | 1.96 (1.88 to 2.04) | < 0.001 |
| Mongolia | 7 (4-10) | 14.42 (8.92-22.75) | 10 (6-15) | 12.19 (7.5-18.86) | -0.77 (-1 to -0.54) | < 0.001 |
| Montenegro | 0 (0-0) | 0.8 (0.49-1.23) | 1 (0-1) | 1.17 (0.66-1.83) | 1.3 (0.73 to 1.86) | < 0.001 |
| Morocco | 74 (38-207) | 15.27 (7.71-43.47) | 318 (179-604) | 26.83 (15.15-51.48) | 1.84 (1.76 to 1.92) | < 0.001 |
| Mozambique | 84 (49-136) | 43.6 (25.15-72.95) | 232 (130-380) | 60.64 (33.58-98.8) | 1.11 (0.85 to 1.37) | < 0.001 |
| Myanmar | 200 (107-372) | 26.08 (13.9-49.84) | 540 (292-914) | 27.72 (14.95-47.19) | 0.19 (0.14 to 0.24) | < 0.001 |
| Namibia | 2 (0-3) | 7.97 (2.09-16.46) | 5 (1-10) | 9.59 (2.72-19.71) | 0.63 (0.47 to 0.79) | < 0.001 |
| Nauru | 0 (0-0) | 37.22 (19.99-66.72) | 0 (0-0) | 40.24 (17.63-143.42) | 0.27 (0.13 to 0.41) | < 0.001 |
| Nepal | 88 (48-166) | 32.95 (17.9-64.88) | 418 (236-670) | 50.84 (28.84-82.45) | 1.43 (1.32 to 1.53) | < 0.001 |
| Netherlands | 498 (397-593) | 40.51 (32.2-48.18) | 1171 (870-1429) | 48.95 (36.65-59.74) | 0.68 (-0.08 to 1.44) | 0.08 |
| New Zealand | 53 (42-63) | 25.03 (19.83-29.74) | 120 (90-147) | 23.21 (17.52-28.53) | 0 (-2.39 to 2.46) | 0.998 |
| Nicaragua | 4 (2-6) | 5.75 (3.55-9.07) | 22 (12-35) | 10.55 (5.88-16.98) | 2.01 (1.46 to 2.55) | < 0.001 |
| Niger | 9 (4-17) | 13.56 (6.09-24.75) | 26 (11-50) | 11.44 (4.65-22.56) | -0.55 (-0.66 to -0.44) | < 0.001 |
| Nigeria | 219 (142-340) | 13.81 (8.99-21.36) | 362 (229-651) | 13.72 (8.78-24.68) | -0.04 (-0.17 to 0.1) | 0.599 |
| Niue | 0 (0-0) | 25.73 (13.68-47.56) | 0 (0-0) | 26.79 (15.5-43.44) | 0.14 (0 to 0.28) | 0.053 |
| North Macedonia | 1 (1-2) | 1.66 (1-2.64) | 2 (1-4) | 1.86 (1.1-3.22) | 0.33 (-0.01 to 0.67) | 0.058 |
| Northern Mariana Islands | 0 (0-0) | 77.28 (46.9-121.66) | 1 (0-1) | 62.63 (38.59-95.63) | -1.3 (-1.7 to -0.9) | < 0.001 |
| Norway | 143 (118-160) | 30.76 (25.46-34.29) | 261 (202-301) | 34.52 (27.06-39.55) | 0.31 (-0.39 to 1) | 0.387 |
| Oman | 4 (2-7) | 19.14 (10.46-33.47) | 15 (7-25) | 30.3 (14.05-49.33) | 1.46 (0.79 to 2.14) | < 0.001 |
| Pakistan | 865 (492-1407) | 52.52 (29.78-86.2) | 2739 (1664-4517) | 81.25 (49.16-138.63) | 1.42 (1.28 to 1.56) | < 0.001 |
| Palau | 0 (0-0) | 30.09 (17.09-50.64) | 0 (0-0) | 41.33 (24-66.53) | 1.05 (0.82 to 1.27) | < 0.001 |
| Palestine | 5 (2-10) | 16.07 (7.64-29.88) | 13 (6-23) | 17.36 (8.01-29.49) | 0.18 (-0.07 to 0.43) | 0.151 |
| Panama | 5 (4-6) | 8.34 (6.66-10.16) | 50 (36-64) | 21.81 (16.18-27.99) | 3.16 (2.34 to 3.98) | < 0.001 |
| Papua New Guinea | 5 (2-9) | 12.32 (6.06-24.9) | 12 (6-28) | 11.43 (5.63-26.75) | -0.24 (-0.33 to -0.15) | < 0.001 |
| Paraguay | 9 (5-16) | 9.93 (4.87-16.5) | 51 (23-100) | 19.12 (8.8-37.67) | 2.23 (1.78 to 2.68) | < 0.001 |
| Peru | 137 (86-225) | 29.12 (18.37-48.11) | 766 (410-1221) | 47.29 (25.16-75.42) | 1.69 (0.34 to 3.05) | 0.014 |
| Philippines | 250 (194-377) | 33.34 (23.68-51.03) | 1083 (823-1593) | 37.74 (28.48-56.97) | 0.31 (-0.08 to 0.71) | 0.123 |
| Poland | 363 (336-389) | 15.37 (14.12-16.5) | 1702 (1397-2005) | 35.4 (29.11-41.97) | 3.06 (0.44 to 5.74) | 0.022 |
| Portugal | 55 (45-65) | 8.18 (6.73-9.77) | 1194 (888-1459) | 60.48 (45.29-73.99) | 7.11 (5.5 to 8.74) | < 0.001 |
| Puerto Rico | 25 (21-30) | 16.01 (13.29-19.01) | 250 (185-321) | 45.85 (34.76-57.88) | 3.53 (2.45 to 4.62) | < 0.001 |
| Qatar | 0 (0-0) | 12.39 (3.37-27.03) | 1 (0-2) | 6.94 (1.56-15.66) | -2.08 (-3.04 to -1.11) | < 0.001 |
| Republic of Korea | 115 (53-267) | 11.74 (5.32-27.83) | 992 (314-1751) | 18.78 (6.01-33.1) | 1.51 (0.99 to 2.03) | < 0.001 |
| Republic of Moldova | 42 (35-49) | 19.42 (16.19-23) | 74 (58-92) | 21.35 (16.84-26.38) | 0.25 (-0.76 to 1.26) | 0.634 |
| Romania | 193 (162-226) | 15.69 (13.14-18.38) | 262 (203-324) | 10.97 (8.49-13.6) | -1.07 (-2.2 to 0.08) | 0.069 |
| Russian Federation | 1577 (1488-1651) | 14.55 (13.69-15.24) | 6424 (5486-7321) | 42.26 (36.21-48.03) | 3.61 (2.53 to 4.71) | < 0.001 |
| Rwanda | 63 (35-106) | 65.3 (35.7-113.81) | 135 (75-222) | 59.94 (33.29-98.34) | -0.28 (-0.39 to -0.17) | < 0.001 |
| Saint Kitts and Nevis | 0 (0-0) | 16.01 (13.19-19.13) | 1 (1-1) | 45.54 (36.23-56.12) | 3.51 (2 to 5.05) | < 0.001 |
| Saint Lucia | 0 (0-0) | 10.24 (8.43-12.25) | 3 (2-4) | 25.28 (19.67-31.62) | 2.82 (1.52 to 4.12) | < 0.001 |
| Saint Vincent and the Grenadines | 1 (1-1) | 23.39 (19.31-27.81) | 3 (2-4) | 52.7 (42.17-64.15) | 2.88 (1.79 to 3.97) | < 0.001 |
| Samoa | 1 (1-2) | 32.23 (17.37-59.72) | 1 (1-2) | 29.01 (16.82-48.22) | -0.36 (-0.49 to -0.23) | < 0.001 |
| San Marino | 0 (0-0) | 6.05 (3.06-11.04) | 0 (0-1) | 5.44 (2.75-9.87) | -0.43 (-1.12 to 0.26) | 0.217 |
| Sao Tome and Principe | 1 (0-1) | 26.15 (13.84-44.24) | 1 (1-2) | 33.13 (13.48-59.37) | 0.79 (0.62 to 0.96) | < 0.001 |
| Saudi Arabia | 74 (41-139) | 47.73 (25.88-89.5) | 177 (98-274) | 68.24 (37.37-105.63) | 1.15 (0.85 to 1.44) | < 0.001 |
| Senegal | 17 (9-29) | 17.08 (8.71-28.18) | 41 (19-79) | 15.98 (7.1-30.67) | -0.22 (-0.48 to 0.04) | 0.102 |
| Serbia | 33 (19-57) | 8.11 (4.56-13.88) | 59 (30-93) | 6.15 (3.17-9.74) | -0.79 (-1.44 to -0.14) | 0.017 |
| Seychelles | 1 (0-2) | 26.27 (14.4-53.71) | 4 (2-6) | 76.39 (39.36-119.46) | 3.41 (2.82 to 4) | < 0.001 |
| Sierra Leone | 10 (5-17) | 14.01 (6.82-24.01) | 15 (7-27) | 12.15 (5.54-23.04) | -0.48 (-0.55 to -0.4) | < 0.001 |
| Singapore | 69 (56-81) | 81.59 (66.59-96.4) | 333 (250-408) | 75.64 (57.28-92.57) | -0.28 (-0.98 to 0.42) | 0.427 |
| Slovakia | 61 (39-90) | 18.84 (12.18-28.12) | 89 (56-132) | 15.74 (9.96-23.41) | -0.7 (-1.08 to -0.33) | < 0.001 |
| Slovenia | 26 (20-33) | 18.33 (14.12-23.19) | 55 (37-77) | 17.49 (11.81-24.92) | -0.12 (-1.04 to 0.8) | 0.793 |
| Solomon Islands | 0 (0-1) | 20.49 (9.85-51.77) | 2 (1-4) | 22.12 (11.37-57.66) | 0.24 (0.16 to 0.32) | < 0.001 |
| Somalia | 31 (17-52) | 50.66 (27.34-86.02) | 111 (60-186) | 55.89 (29.91-93.66) | 0.33 (0.26 to 0.4) | < 0.001 |
| South Africa | 58 (31-93) | 6.67 (3.53-10.71) | 165 (94-302) | 8.52 (4.81-15.59) | 0.88 (0.48 to 1.28) | < 0.001 |
| South Sudan | 36 (20-60) | 46.78 (25.45-77.94) | 66 (33-107) | 65.72 (33.21-107.42) | 1.12 (1 to 1.24) | < 0.001 |
| Spain | 575 (465-681) | 18.98 (15.29-22.47) | 3826 (2777-4732) | 44.63 (32.69-55.36) | 2.97 (2.43 to 3.51) | < 0.001 |
| Sri Lanka | 57 (24-117) | 18.46 (7.73-37.44) | 265 (116-492) | 21.26 (9.18-39.62) | 0.43 (-0.12 to 0.99) | 0.123 |
| Sudan | 35 (18-93) | 12.42 (6.14-33.42) | 89 (49-162) | 17.01 (9.53-31.37) | 1.02 (0.93 to 1.11) | < 0.001 |
| Suriname | 2 (1-4) | 21.07 (12.67-36.65) | 12 (6-19) | 40.13 (21.61-65.92) | 2.24 (1.76 to 2.72) | < 0.001 |
| Sweden | 233 (189-272) | 23.18 (18.83-27.09) | 270 (203-331) | 17.2 (13.1-21.12) | -0.92 (-1.77 to -0.07) | 0.034 |
| Switzerland | 55 (43-66) | 7.86 (6.26-9.51) | 267 (189-336) | 18.52 (13.28-23.32) | 2.95 (1.42 to 4.5) | < 0.001 |
| Syrian Arab Republic | 125 (76-225) | 83.92 (51.03-150.02) | 241 (133-383) | 83.34 (42.93-135.48) | -0.18 (-0.67 to 0.31) | 0.46 |
| Taiwan (Province of China) | 155 (130-184) | 33.77 (27.94-40.09) | 2027 (1521-2502) | 87.26 (66.06-107.36) | 3.08 (2.23 to 3.93) | < 0.001 |
| Tajikistan | 22 (14-36) | 18.07 (11.26-29.89) | 36 (22-56) | 21.61 (12.93-33.59) | 0.59 (0.14 to 1.04) | 0.01 |
| Thailand | 422 (150-943) | 33.01 (11.57-75.02) | 4113 (2169-6539) | 72.66 (38.68-115.13) | 2.55 (2.13 to 2.98) | < 0.001 |
| Timor-Leste | 1 (1-3) | 21.14 (10.79-39.04) | 7 (4-12) | 22.48 (12.27-38.33) | 0.19 (0.06 to 0.33) | 0.005 |
| Togo | 5 (3-9) | 14.38 (7.21-24.61) | 17 (8-31) | 14.15 (6.25-26.68) | -0.06 (-0.17 to 0.05) | 0.295 |
| Tokelau | 0 (0-0) | 23.98 (12.64-47.85) | 0 (0-0) | 23.34 (12-49.9) | -0.07 (-0.17 to 0.03) | 0.154 |
| Tonga | 0 (0-0) | 8.66 (4.93-14.27) | 0 (0-1) | 10.51 (5.87-17.16) | 0.67 (0.22 to 1.13) | 0.004 |
| Trinidad and Tobago | 4 (3-4) | 12.08 (10.09-14.41) | 25 (18-32) | 26.42 (19.71-34.08) | 2.68 (1.34 to 4.03) | < 0.001 |
| Tunisia | 20 (11-40) | 14.23 (7.94-29.84) | 100 (55-187) | 18.58 (10.14-35.23) | 0.88 (0.79 to 0.98) | < 0.001 |
| Turkey | 184 (99-405) | 14.36 (7.71-31.7) | 775 (484-1162) | 19.21 (11.97-28.85) | 1.15 (-0.1 to 2.42) | 0.072 |
| Turkmenistan | 9 (8-11) | 11.24 (9.41-13.24) | 77 (57-100) | 47.07 (35.34-61.24) | 4.92 (3.26 to 6.6) | < 0.001 |
| Tuvalu | 0 (0-0) | 24.34 (12.79-49.99) | 0 (0-0) | 24.99 (13.76-48.04) | 0.09 (0.04 to 0.13) | < 0.001 |
| Uganda | 72 (38-124) | 33.33 (17.29-57.42) | 256 (148-415) | 47.86 (27.49-77.48) | 1.2 (0.97 to 1.44) | < 0.001 |
| Ukraine | 403 (339-473) | 9.19 (7.73-10.8) | 480 (327-677) | 9.84 (6.68-13.91) | 0.17 (-1.32 to 1.69) | 0.821 |
| United Arab Emirates | 2 (1-5) | 27.59 (14.13-52.31) | 14 (8-22) | 113.53 (59.05-181.16) | 4.83 (1.52 to 8.25) | 0.004 |
| United Kingdom | 695 (612-745) | 11.93 (10.49-12.8) | 3850 (3072-4305) | 42.91 (34.7-47.82) | 4.58 (3.68 to 5.49) | < 0.001 |
| United Republic of Tanzania | 180 (101-288) | 49.88 (27.93-80.76) | 493 (287-766) | 55.31 (31.86-86.07) | 0.36 (0.2 to 0.52) | < 0.001 |
| United States of America | 6477 (5337-7109) | 31.76 (26.28-34.81) | 12224 (9735-13759) | 34.54 (27.9-38.75) | 0.27 (-0.29 to 0.83) | 0.345 |
| United States Virgin Islands | 1 (1-2) | 35.9 (21.77-55.37) | 3 (2-4) | 27.71 (16.51-46.4) | -0.88 (-1.15 to -0.62) | < 0.001 |
| Uruguay | 26 (22-31) | 12.43 (10.25-14.76) | 441 (342-528) | 103.5 (81.02-123.73) | 6.88 (5.49 to 8.29) | < 0.001 |
| Uzbekistan | 41 (33-49) | 7.48 (6.07-8.97) | 107 (82-134) | 11.32 (8.69-14.27) | 0.95 (-2.52 to 4.53) | 0.596 |
| Vanuatu | 0 (0-0) | 19.22 (10.09-36.4) | 1 (0-2) | 19.73 (10.89-38.99) | 0.08 (0.01 to 0.15) | 0.034 |
| Venezuela (Bolivarian Republic of) | 22 (18-26) | 6 (4.88-7.23) | 263 (191-343) | 18.66 (13.66-24.31) | 3.7 (2.57 to 4.84) | < 0.001 |
| Viet Nam | 281 (136-495) | 14.71 (7.16-25.85) | 746 (340-1260) | 17.2 (7.88-29.03) | 0.53 (0.45 to 0.61) | < 0.001 |
| Yemen | 16 (8-42) | 10.43 (4.97-28.33) | 54 (28-120) | 13.27 (6.9-30.01) | 0.79 (0.65 to 0.94) | < 0.001 |
| Zambia | 35 (21-54) | 45.34 (26.96-70.8) | 107 (61-168) | 48.38 (27.66-76.29) | 0.23 (0.04 to 0.42) | 0.016 |
| Zimbabwe | 8 (2-15) | 6.41 (1.88-12.23) | 16 (5-32) | 7.55 (2.08-15.61) | 0.58 (0.35 to 0.8) | < 0.001 |

Abbreviation: AAPC, average annual percentage change.

**Table S7. The case number and age-standardized rate of DALYs of urinary tract infections in 1990 and 2021, and the temporal trends between 1990 and 2021 at the national level.**

| Location | Number of cases, 1990 | Age-standardized rate per 100 000 population, 1990 | Number of cases, 2021 | Age-standardized rate per 100 000 population, 2021 | AAPC, 1990-2021 | p value |
| --- | --- | --- | --- | --- | --- | --- |
| Afghanistan | 963 (493-2335) | 440.11 (224.07-1087.62) | 1627 (857-3512) | 517.57 (271.59-1106.73) | 0.52 (0.42 to 0.61) | < 0.001 |
| Albania | 65 (40-101) | 74.61 (45.58-116.82) | 106 (58-180) | 49.59 (27.04-84.35) | -1.43 (-1.75 to -1.1) | < 0.001 |
| Algeria | 863 (441-2329) | 310.82 (161.93-788.51) | 3736 (2249-6188) | 397.62 (241.76-652.69) | 0.88 (0.64 to 1.12) | < 0.001 |
| American Samoa | 6 (3-9) | 890.5 (545.74-1428.84) | 15 (9-22) | 912.19 (559.99-1399.07) | 0.24 (-0.53 to 1.02) | 0.542 |
| Andorra | 9 (5-16) | 395.11 (210.58-772.8) | 25 (14-39) | 286.76 (161.76-450.13) | -1.13 (-1.46 to -0.81) | < 0.001 |
| Angola | 225 (104-528) | 200.45 (91.45-483.12) | 848 (403-1869) | 228.11 (107.36-506.97) | 0.43 (0.25 to 0.61) | < 0.001 |
| Antigua and Barbuda | 5 (4-5) | 137.85 (112.91-165.46) | 26 (21-31) | 576.09 (475.44-688.47) | 4.67 (3.31 to 6.04) | < 0.001 |
| Argentina | 2139 (1783-2533) | 135.86 (112.81-160.94) | 37459 (30210-44805) | 1145.28 (926.52-1368.73) | 6.94 (5.71 to 8.18) | < 0.001 |
| Armenia | 193 (151-238) | 166.66 (129.73-206.49) | 623 (486-794) | 266.87 (208.08-339.99) | 1.6 (-0.18 to 3.42) | 0.078 |
| Australia | 2414 (1992-2821) | 229.95 (189.28-268.84) | 8832 (6622-10746) | 307.37 (233.05-373.6) | 1.13 (-0.41 to 2.69) | 0.151 |
| Austria | 1742 (1434-2049) | 222.16 (182.56-261.74) | 2409 (1810-2977) | 187.64 (142.23-232.27) | -0.47 (-1.78 to 0.85) | 0.482 |
| Azerbaijan | 321 (212-494) | 142.91 (94.21-219.21) | 1040 (566-1537) | 261.87 (141.03-390.58) | 2.08 (1.83 to 2.33) | < 0.001 |
| Bahamas | 17 (14-20) | 235.6 (191.16-285.55) | 149 (115-188) | 870.71 (676.62-1101.89) | 4.27 (3.05 to 5.52) | < 0.001 |
| Bahrain | 4 (1-10) | 112.2 (33.02-280.82) | 32 (11-74) | 186.7 (57.93-438.03) | 1.66 (0.47 to 2.86) | 0.006 |
| Bangladesh | 7570 (4389-13863) | 532.82 (306.33-1000.27) | 24241 (14568-38301) | 484.99 (291.39-767.6) | -0.18 (-0.79 to 0.43) | 0.559 |
| Barbados | 58 (48-69) | 322.89 (267.68-385.03) | 322 (249-405) | 1193.25 (923.11-1499.78) | 4.37 (3.33 to 5.42) | < 0.001 |
| Belarus | 1738 (1446-2071) | 229.74 (191.14-273.12) | 3510 (2720-4430) | 350.51 (271.49-443.42) | 0.98 (-1.47 to 3.49) | 0.437 |
| Belgium | 1158 (935-1381) | 122.59 (98.7-146.18) | 7881 (5787-9748) | 470.66 (353.99-578.51) | 4.79 (3.23 to 6.37) | < 0.001 |
| Belize | 7 (6-8) | 161.92 (132.26-195.33) | 62 (49-75) | 583.97 (464.93-709.8) | 4.29 (3.3 to 5.28) | < 0.001 |
| Benin | 183 (94-312) | 237.16 (121.65-404.13) | 396 (186-755) | 212.16 (99.17-403.01) | -0.35 (-0.43 to -0.26) | < 0.001 |
| Bermuda | 3 (3-4) | 111.96 (91.01-135.2) | 20 (15-26) | 226.09 (168.53-296.02) | 2.31 (1.3 to 3.32) | < 0.001 |
| Bhutan | 58 (28-103) | 724.17 (350.26-1313.25) | 244 (117-408) | 1006.69 (483.31-1684.59) | 1.09 (0.92 to 1.25) | < 0.001 |
| Bolivia (Plurinational State of) | 585 (349-956) | 492.93 (292.85-823.5) | 2570 (1461-4268) | 718.85 (412.33-1190.55) | 1.24 (1.11 to 1.38) | < 0.001 |
| Bosnia and Herzegovina | 447 (265-670) | 259.25 (152.39-391.08) | 488 (266-940) | 139.76 (76.21-270.58) | -1.91 (-2.33 to -1.48) | < 0.001 |
| Botswana | 24 (6-51) | 123.82 (31.74-262.75) | 86 (25-174) | 159.15 (46.54-324.23) | 0.77 (-0.12 to 1.68) | 0.092 |
| Brazil | 14097 (12622-15355) | 412.18 (365.04-450.34) | 184473 (152604-206739) | 1480.83 (1228.38-1657.79) | 4.28 (3.56 to 5) | < 0.001 |
| Brunei Darussalam | 29 (15-50) | 884.82 (453.91-1540.79) | 103 (59-160) | 1025.96 (568.86-1599.1) | 0.36 (0.06 to 0.66) | 0.018 |
| Bulgaria | 3751 (3201-4385) | 642.81 (545.42-757.23) | 1940 (1543-2427) | 228.7 (181.99-285.82) | -3.04 (-4.17 to -1.89) | < 0.001 |
| Burkina Faso | 386 (195-691) | 254.22 (128.21-449.63) | 710 (326-1356) | 212.96 (96.18-407.96) | -0.55 (-0.75 to -0.34) | < 0.001 |
| Burundi | 724 (424-1196) | 742.8 (432.2-1238.97) | 1041 (581-1734) | 696.02 (387.37-1172.6) | -0.23 (-0.39 to -0.06) | 0.007 |
| Cabo Verde | 10 (5-17) | 78.28 (36.46-139.4) | 23 (11-43) | 108.06 (50.21-197.35) | 1.05 (0.92 to 1.18) | < 0.001 |
| Cambodia | 693 (370-1259) | 390.66 (207.55-712.85) | 2249 (1328-3525) | 432.17 (255.13-680.53) | 0.34 (0.28 to 0.39) | < 0.001 |
| Cameroon | 610 (302-1068) | 417.17 (204.55-725.18) | 1171 (521-2266) | 287.66 (126.83-558) | -1.21 (-1.33 to -1.1) | < 0.001 |
| Canada | 4170 (3439-4918) | 231.01 (190.46-272.46) | 14637 (11481-17483) | 326.06 (258.3-388.89) | 1.3 (0.86 to 1.74) | < 0.001 |
| Central African Republic | 93 (42-219) | 262.02 (118.39-639.91) | 167 (74-409) | 252.85 (111.12-626.77) | -0.13 (-0.23 to -0.03) | 0.008 |
| Chad | 195 (92-359) | 173.69 (80.58-319.54) | 260 (114-533) | 160 (69.69-326.66) | -0.26 (-0.34 to -0.18) | < 0.001 |
| Chile | 3429 (2873-4011) | 776.02 (647.6-908.3) | 13305 (10726-15880) | 966.44 (782.14-1151.95) | 0.65 (-0.56 to 1.88) | 0.295 |
| China | 27134 (16702-37596) | 88.81 (53.59-123.62) | 40280 (27135-69917) | 41.33 (27.77-71.78) | -2.43 (-2.79 to -2.06) | < 0.001 |
| Colombia | 2162 (1820-2521) | 325.2 (272.77-379.61) | 14862 (11302-18197) | 530.33 (406.2-647.81) | 1.54 (0.06 to 3.03) | 0.042 |
| Comoros | 63 (37-101) | 893.45 (515.3-1432.11) | 204 (119-321) | 1021.8 (595.18-1607.98) | 0.44 (0.29 to 0.59) | < 0.001 |
| Congo | 135 (67-267) | 346.17 (171.78-702.05) | 281 (143-505) | 338.54 (171.99-613.25) | -0.07 (-0.27 to 0.13) | 0.509 |
| Cook Islands | 0 (0-0) | 36.88 (22.61-59.75) | 0 (0-0) | 25.82 (15.75-40.57) | -1.09 (-1.62 to -0.56) | < 0.001 |
| Costa Rica | 146 (120-175) | 197.43 (163.23-236.82) | 1413 (1108-1718) | 519.36 (410.08-630.65) | 3.06 (1.94 to 4.19) | < 0.001 |
| Coted'Ivoire | 228 (116-391) | 234.41 (119.2-397.03) | 677 (324-1235) | 215.09 (102.32-392.08) | -0.29 (-0.51 to -0.07) | 0.011 |
| Croatia | 1722 (1451-2025) | 555.33 (467.54-652.59) | 4386 (3552-5429) | 754.26 (608.18-940.73) | 0.98 (-0.64 to 2.62) | 0.237 |
| Cuba | 305 (252-366) | 68.45 (56.42-81.97) | 2033 (1630-2498) | 196.16 (157.64-241.14) | 3.46 (2.35 to 4.58) | < 0.001 |
| Cyprus | 498 (280-835) | 1727.4 (967.92-2935.08) | 816 (503-1217) | 940.87 (566.95-1409.63) | -1.83 (-2.46 to -1.19) | < 0.001 |
| Czechia | 6037 (5122-7016) | 738.82 (626.5-858.85) | 5256 (4146-6529) | 389.83 (307.4-486.2) | -1.98 (-3.37 to -0.57) | 0.006 |
| Democratic People's Republic of Korea | 811 (382-1753) | 115.56 (53.62-258.93) | 1509 (671-3550) | 89.33 (39.76-211.92) | -0.83 (-0.87 to -0.78) | < 0.001 |
| Democratic Republic of the Congo | 1129 (534-2507) | 232.05 (108.01-543.94) | 2900 (1279-6531) | 231.91 (101.82-524.07) | -0.02 (-0.08 to 0.04) | 0.478 |
| Denmark | 1223 (1013-1433) | 242.47 (201.09-284.14) | 3657 (2837-4470) | 469.83 (367.38-573.46) | 2.18 (0.7 to 3.68) | 0.004 |
| Djibouti | 23 (13-40) | 597.94 (325.4-1036.78) | 150 (85-244) | 875.33 (495.01-1418.65) | 1.23 (1.16 to 1.31) | < 0.001 |
| Dominica | 3 (2-4) | 78.32 (49.81-117.46) | 7 (4-12) | 175.63 (97.12-284.57) | 2.65 (2.44 to 2.87) | < 0.001 |
| Dominican Republic | 56 (36-99) | 45.73 (28.76-78.56) | 242 (142-395) | 53.58 (31.49-87.4) | 0.56 (-0.11 to 1.24) | 0.099 |
| Ecuador | 445 (365-537) | 233.3 (191.02-281.35) | 3418 (2539-4634) | 468.05 (349.71-630.77) | 2.31 (0.3 to 4.35) | 0.024 |
| Egypt | 258 (119-631) | 41.86 (18.91-103.91) | 1971 (980-3141) | 160.67 (79.62-253.91) | 4.46 (3.92 to 5) | < 0.001 |
| El Salvador | 369 (241-567) | 278.05 (182.29-427.39) | 1508 (894-2348) | 441.7 (262.48-686.13) | 1.47 (1.15 to 1.8) | < 0.001 |
| Equatorial Guinea | 17 (8-36) | 237.02 (108.6-530.67) | 62 (30-115) | 352.23 (171.53-656.03) | 1.3 (1.13 to 1.47) | < 0.001 |
| Eritrea | 215 (117-376) | 600.81 (321.82-1043.96) | 791 (441-1380) | 777.38 (434.12-1358.87) | 0.83 (0.76 to 0.9) | < 0.001 |
| Estonia | 754 (631-892) | 600.45 (503.61-709.62) | 815 (639-1012) | 436.52 (343.37-542.44) | -1.06 (-2.24 to 0.14) | 0.084 |
| Eswatini | 23 (6-45) | 207.86 (58.37-409.81) | 48 (13-96) | 214.32 (58.99-424.31) | 0.14 (-0.07 to 0.35) | 0.194 |
| Ethiopia | 12124 (8234-17902) | 1949.99 (1308.56-2923.51) | 23873 (16543-32832) | 1565.57 (1079.67-2156.96) | -0.71 (-0.89 to -0.53) | < 0.001 |
| Fiji | 16 (9-26) | 152.43 (89.05-252.04) | 64 (38-103) | 245.28 (147.82-394.11) | 1.61 (1.08 to 2.15) | < 0.001 |
| Finland | 2189 (1786-2556) | 477.66 (388.86-558.25) | 1654 (1244-2061) | 188.67 (144.76-233.19) | -2.76 (-3.99 to -1.5) | < 0.001 |
| France | 11908 (9739-13862) | 215.86 (176.77-251.1) | 24868 (18806-30358) | 216.81 (165.61-265.27) | 0 (-0.74 to 0.75) | 0.999 |
| Gabon | 76 (37-160) | 316.12 (152-672.21) | 169 (84-323) | 463.09 (230.66-882.29) | 1.27 (1.02 to 1.52) | < 0.001 |
| Gambia | 26 (14-43) | 222.24 (117.01-369.78) | 87 (41-165) | 235.91 (110.75-452.19) | 0.2 (-0.32 to 0.72) | 0.449 |
| Georgia | 158 (131-188) | 46.97 (39.03-55.83) | 526 (409-696) | 147.08 (114.72-194.77) | 3.89 (0.71 to 7.17) | 0.016 |
| Germany | 11160 (9191-13077) | 135.97 (111.87-159.41) | 40094 (30672-49014) | 309.19 (239.65-376.99) | 2.77 (1.63 to 3.93) | < 0.001 |
| Ghana | 348 (190-612) | 166.05 (91.27-291.56) | 3030 (1426-5279) | 489.58 (233.53-849.87) | 3.56 (3.37 to 3.75) | < 0.001 |
| Greece | 1856 (1536-2197) | 248.16 (204.88-293.94) | 7275 (5769-8702) | 412.8 (329.5-492.6) | 1.67 (-0.31 to 3.69) | 0.098 |
| Greenland | 9 (6-14) | 862.04 (512.43-1359.67) | 13 (6-21) | 619.81 (298.39-982.18) | -1.08 (-1.49 to -0.66) | < 0.001 |
| Grenada | 7 (5-8) | 142.95 (116.57-170.47) | 26 (20-32) | 505.73 (401.01-621.2) | 4.29 (3.24 to 5.34) | < 0.001 |
| Guam | 18 (11-27) | 982.55 (602.99-1494.2) | 29 (18-44) | 260.02 (165.06-383.63) | -4.52 (-6.46 to -2.54) | < 0.001 |
| Guatemala | 184 (154-219) | 176.07 (147.12-209.93) | 1645 (1308-2028) | 358.48 (285.04-440.61) | 2.38 (0.15 to 4.66) | 0.036 |
| Guinea | 305 (151-536) | 238.57 (117.17-419.93) | 410 (190-783) | 212.17 (98.03-404.26) | -0.38 (-0.45 to -0.3) | < 0.001 |
| Guinea-Bissau | 36 (19-62) | 278.91 (143.43-479.52) | 53 (26-106) | 235.76 (110-466.39) | -0.54 (-0.62 to -0.47) | < 0.001 |
| Guyana | 22 (18-27) | 154.41 (123.2-190.73) | 123 (90-160) | 496.17 (358.04-646.8) | 4.03 (2.83 to 5.24) | < 0.001 |
| Haiti | 277 (153-570) | 250.58 (137.47-536.16) | 735 (372-1468) | 317.99 (161.18-638.44) | 0.77 (0.7 to 0.84) | < 0.001 |
| Honduras | 315 (199-504) | 410.64 (258.87-659.15) | 2050 (1024-3321) | 815.5 (404.92-1316.16) | 2.27 (1.66 to 2.89) | < 0.001 |
| Hungary | 3528 (2969-4121) | 424.73 (357-496.21) | 3583 (2842-4415) | 277.01 (219.84-341.81) | -1.01 (-2.04 to 0.04) | 0.06 |
| Iceland | 16 (11-20) | 93.82 (68.95-120.28) | 96 (71-120) | 272.31 (202.66-339.69) | 3.78 (3.35 to 4.21) | < 0.001 |
| India | 74381 (54252-98249) | 489.46 (357.2-653.97) | 324548 (233752-414863) | 659.33 (475.83-845.15) | 1.06 (0.43 to 1.69) | 0.001 |
| Indonesia | 5778 (3942-8578) | 170.21 (115.84-255.21) | 20872 (14594-28816) | 251.42 (177.34-355.85) | 1.28 (1.17 to 1.39) | < 0.001 |
| Iran (Islamic Republic of) | 1642 (1176-2905) | 238.13 (168.49-423.49) | 6273 (4484-10473) | 232.18 (165.4-387.14) | -0.09 (-0.29 to 0.11) | 0.373 |
| Iraq | 265 (139-513) | 82.36 (43.23-158.95) | 677 (379-1061) | 87.77 (48.93-137.38) | 0.29 (0.03 to 0.55) | 0.029 |
| Ireland | 793 (661-931) | 363.52 (302.19-427.3) | 1763 (1332-2202) | 394.5 (300.41-492.03) | 0.71 (-0.88 to 2.32) | 0.386 |
| Israel | 585 (484-695) | 263.48 (216-313.43) | 4980 (3742-6134) | 669.72 (508.04-825.32) | 3.03 (1.69 to 4.38) | < 0.001 |
| Italy | 3193 (2744-3507) | 62.88 (53.81-69.2) | 14354 (10579-17413) | 130.89 (98.95-159.59) | 2.42 (0.65 to 4.23) | 0.007 |
| Jamaica | 106 (88-126) | 110.87 (91.32-131) | 630 (466-809) | 391.03 (290.93-501.71) | 4.17 (2.87 to 5.48) | < 0.001 |
| Japan | 11881 (10165-12874) | 138.53 (117.15-150.84) | 61401 (42641-73255) | 177.88 (130.55-208.65) | 0.76 (0.11 to 1.42) | 0.022 |
| Jordan | 61 (37-106) | 160.51 (95.3-281.61) | 360 (223-549) | 186.08 (113.64-286.4) | 0.5 (-0.88 to 1.89) | 0.48 |
| Kazakhstan | 1375 (1156-1588) | 209.99 (176.23-242.86) | 3115 (2212-4266) | 348.69 (245.48-480.46) | 1.73 (0.16 to 3.32) | 0.03 |
| Kenya | 1987 (1483-2975) | 664.05 (492.37-995.22) | 8627 (5834-12426) | 998.53 (676.46-1434.74) | 1.35 (1.21 to 1.48) | < 0.001 |
| Kiribati | 4 (2-6) | 288.19 (164.53-502.47) | 8 (5-13) | 345.27 (202.76-568.06) | 0.6 (0.42 to 0.78) | < 0.001 |
| Kuwait | 4 (3-5) | 28.76 (21.76-35.88) | 215 (146-282) | 316.21 (215.14-417.46) | 7.62 (2.93 to 12.52) | 0.001 |
| Kyrgyzstan | 362 (303-424) | 236.93 (198.23-277.16) | 466 (350-613) | 231.3 (173.6-304.36) | -0.04 (-2.71 to 2.69) | 0.975 |
| Lao People's Democratic Republic | 327 (167-629) | 429.9 (218.83-845.51) | 632 (345-1049) | 393.25 (213.01-654.91) | -0.31 (-0.35 to -0.26) | < 0.001 |
| Latvia | 1174 (966-1398) | 535.28 (440.17-637.95) | 1312 (980-1753) | 477.91 (357.19-641.81) | -0.36 (-1.5 to 0.79) | 0.537 |
| Lebanon | 621 (371-977) | 788.62 (470.57-1242.08) | 2142 (1335-3342) | 657.54 (409.64-1023.93) | -0.58 (-0.85 to -0.31) | < 0.001 |
| Lesotho | 53 (16-106) | 126.07 (36.85-252.29) | 121 (34-236) | 250.16 (70.74-491.45) | 2.34 (1.91 to 2.79) | < 0.001 |
| Liberia | 125 (66-210) | 316.57 (163.28-530.63) | 165 (73-303) | 268.57 (118.4-491.51) | -0.57 (-0.77 to -0.37) | < 0.001 |
| Libya | 148 (72-338) | 222.38 (107.79-505.67) | 599 (331-969) | 355.44 (196.58-575.02) | 1.57 (1.15 to 1.99) | < 0.001 |
| Lithuania | 789 (650-932) | 309.7 (254.97-365.76) | 1327 (1034-1730) | 349.83 (271.32-462.78) | 0.43 (-0.66 to 1.54) | 0.441 |
| Luxembourg | 22 (18-27) | 70.06 (56.91-84.62) | 99 (76-122) | 149.53 (116.92-185.64) | 2.6 (1.55 to 3.65) | < 0.001 |
| Madagascar | 941 (560-1574) | 541.2 (320.91-917.76) | 1868 (1023-3003) | 546.05 (298.97-874.49) | 0 (-0.22 to 0.23) | 0.999 |
| Malawi | 937 (558-1527) | 632.59 (372.4-1041.67) | 2184 (1294-3527) | 721.58 (427.65-1160.36) | 0.46 (0.27 to 0.65) | < 0.001 |
| Malaysia | 993 (333-1981) | 279.59 (93.25-559.31) | 3854 (1432-8368) | 373.83 (138.03-820.17) | 0.93 (0.44 to 1.42) | < 0.001 |
| Maldives | 10 (6-18) | 537.3 (314.15-1007.02) | 39 (24-60) | 384.17 (233.65-589.31) | -1.06 (-1.44 to -0.68) | < 0.001 |
| Mali | 334 (166-601) | 263.07 (129.33-469.46) | 631 (293-1243) | 229.2 (103.39-450.86) | -0.45 (-0.54 to -0.35) | < 0.001 |
| Malta | 45 (37-54) | 215.18 (175.63-257.98) | 243 (180-308) | 390.04 (291.92-494.56) | 1.94 (1.58 to 2.3) | < 0.001 |
| Marshall Islands | 2 (1-3) | 284.87 (151.68-583.41) | 3 (2-5) | 331.34 (187.29-618.04) | 0.48 (0.43 to 0.53) | < 0.001 |
| Mauritania | 155 (82-256) | 356.65 (185.13-592.13) | 217 (97-410) | 290.25 (130.03-545.8) | -0.69 (-0.81 to -0.58) | < 0.001 |
| Mauritius | 28 (23-33) | 93.27 (76.79-110.58) | 303 (245-367) | 336.6 (272.57-407.52) | 5.55 (4.93 to 6.17) | < 0.001 |
| Mexico | 6952 (6412-7594) | 478.01 (438.41-524.54) | 55076 (46380-66148) | 989.05 (833.05-1186.54) | 2.41 (1.81 to 3.01) | < 0.001 |
| Micronesia (Federated States of) | 9 (5-21) | 496.25 (258.19-1218.17) | 11 (6-20) | 493.48 (281.93-906.39) | -0.03 (-0.1 to 0.05) | 0.472 |
| Monaco | 1 (1-2) | 25.82 (14.8-41.95) | 3 (2-5) | 42.71 (25.25-68.81) | 1.63 (1.55 to 1.72) | < 0.001 |
| Mongolia | 119 (74-185) | 244.06 (152.98-381.24) | 167 (105-256) | 196.16 (122.59-300.85) | -0.81 (-1.32 to -0.29) | 0.002 |
| Montenegro | 5 (4-8) | 18.39 (12.21-26.05) | 11 (7-16) | 22.17 (14.43-32.4) | 0.65 (0.28 to 1.02) | 0.001 |
| Morocco | 1143 (596-3025) | 220.38 (114.05-596.39) | 4710 (2674-8674) | 371.37 (210.75-691.72) | 1.7 (1.61 to 1.79) | < 0.001 |
| Mozambique | 1515 (889-2447) | 701.97 (409.18-1151.44) | 4112 (2338-6768) | 971.62 (546.62-1590.77) | 1.07 (0.84 to 1.3) | < 0.001 |
| Myanmar | 3519 (1900-6396) | 405.02 (218.09-752.92) | 8492 (4633-14217) | 407.57 (222.01-685.25) | 0.01 (-0.04 to 0.07) | 0.613 |
| Namibia | 28 (8-58) | 116.45 (31.83-240.05) | 74 (21-150) | 135.94 (39.36-276.66) | 0.52 (0.37 to 0.68) | < 0.001 |
| Nauru | 1 (0-1) | 534.99 (284.62-986.99) | 1 (0-3) | 558.26 (254.52-1803.05) | 0.15 (0.06 to 0.23) | < 0.001 |
| Nepal | 1580 (883-2874) | 523.25 (289.75-985.07) | 6897 (3899-10955) | 751.69 (426.49-1203.65) | 1.19 (1.08 to 1.31) | < 0.001 |
| Netherlands | 5981 (4832-7100) | 486.55 (392.67-577.17) | 12662 (9570-15431) | 560.03 (427.23-681.93) | 0.53 (-0.26 to 1.32) | 0.189 |
| New Zealand | 710 (574-839) | 328.97 (265.19-389.16) | 1382 (1059-1689) | 281.81 (218.1-343.72) | -0.11 (-2.88 to 2.75) | 0.94 |
| Nicaragua | 56 (36-87) | 89.36 (57.47-138.38) | 339 (196-538) | 160.62 (92.19-254.66) | 1.9 (1.4 to 2.39) | < 0.001 |
| Niger | 162 (75-300) | 206.61 (95.42-377.52) | 445 (191-878) | 171.39 (72.13-337.16) | -0.6 (-0.68 to -0.52) | < 0.001 |
| Nigeria | 3568 (2314-5548) | 204.67 (133.13-317.27) | 5777 (3657-10335) | 198.65 (126.92-354.3) | -0.12 (-0.26 to 0.03) | 0.11 |
| Niue | 0 (0-1) | 349.88 (188.08-636.59) | 0 (0-1) | 359.03 (207.79-578.28) | 0.09 (-0.05 to 0.23) | 0.22 |
| North Macedonia | 25 (16-36) | 32.25 (21.47-47.53) | 48 (32-77) | 31.84 (20.79-51.34) | -0.01 (-0.35 to 0.33) | 0.947 |
| Northern Mariana Islands | 3 (2-5) | 1053.09 (637.09-1650.3) | 12 (7-19) | 827.97 (513.41-1273.61) | -1.23 (-1.87 to -0.59) | < 0.001 |
| Norway | 1738 (1468-1923) | 381.83 (323.72-422.08) | 2715 (2133-3109) | 389.14 (311.21-443.87) | -0.02 (-0.7 to 0.68) | 0.966 |
| Oman | 67 (37-115) | 294.95 (163.72-508.54) | 243 (123-392) | 442.91 (217.15-717.2) | 1.3 (0.71 to 1.89) | < 0.001 |
| Pakistan | 14958 (8628-24095) | 823.04 (472.93-1334.41) | 46360 (28340-74548) | 1234.85 (752.9-2035.61) | 1.33 (1.25 to 1.41) | < 0.001 |
| Palau | 1 (1-2) | 412.97 (235.06-688.34) | 3 (2-5) | 528.98 (302.57-856.86) | 0.82 (0.6 to 1.04) | < 0.001 |
| Palestine | 74 (35-136) | 218.23 (104.38-402.66) | 190 (90-321) | 230.22 (108.37-388.71) | 0.1 (-0.17 to 0.37) | 0.471 |
| Panama | 80 (65-97) | 129.47 (105-157.13) | 677 (509-862) | 313.85 (237.48-399.39) | 2.85 (0.63 to 5.11) | 0.011 |
| Papua New Guinea | 90 (44-176) | 185.36 (91.54-369.77) | 213 (106-488) | 169.51 (84.47-391.22) | -0.29 (-0.42 to -0.16) | < 0.001 |
| Paraguay | 133 (69-215) | 139.69 (71.81-225.36) | 675 (322-1306) | 259.18 (123.75-500.47) | 2.13 (1.73 to 2.52) | < 0.001 |
| Peru | 1861 (1191-3018) | 390.13 (249.56-634.36) | 10102 (5400-16007) | 645.38 (343.96-1023) | 1.73 (0.32 to 3.16) | 0.016 |
| Philippines | 3969 (3114-5943) | 444.58 (328.69-673.39) | 16883 (12900-24369) | 533.49 (405.89-784.67) | 0.53 (0.19 to 0.87) | 0.002 |
| Poland | 6167 (5759-6592) | 256.15 (238.39-274.19) | 22179 (18325-26498) | 483.57 (400.17-581.29) | 2.39 (0.17 to 4.66) | 0.035 |
| Portugal | 804 (669-959) | 111.67 (92.46-133.14) | 13073 (9820-15988) | 709.81 (537.82-868.93) | 6.55 (5.1 to 8.03) | < 0.001 |
| Puerto Rico | 359 (299-426) | 212.72 (177.42-252.04) | 2944 (2229-3738) | 600.81 (462.29-754.41) | 3.49 (1.79 to 5.22) | < 0.001 |
| Qatar | 2 (1-5) | 163.8 (45.7-355.82) | 12 (4-27) | 100.61 (26.77-221.74) | -1.53 (-2.47 to -0.58) | 0.002 |
| Republic of Korea | 1746 (857-3935) | 156.35 (74.71-359.14) | 11413 (3963-19906) | 219.72 (77.49-382.29) | 1.07 (0.53 to 1.62) | < 0.001 |
| Republic of Moldova | 782 (659-917) | 339.62 (285.25-398.78) | 1316 (1049-1611) | 381.62 (304.53-466.98) | 0.3 (-0.71 to 1.33) | 0.559 |
| Romania | 3516 (2963-4092) | 261.05 (219.77-303.92) | 3953 (3061-4937) | 170.28 (131.56-213.41) | -1.28 (-2.22 to -0.33) | 0.009 |
| Russian Federation | 29920 (28403-31255) | 274.73 (260.58-287.05) | 96468 (82842-110750) | 642.21 (553.44-734.89) | 2.86 (1.88 to 3.84) | < 0.001 |
| Rwanda | 1186 (659-2004) | 1084.81 (598.9-1858.94) | 2381 (1319-3910) | 939.35 (521.63-1541.01) | -0.47 (-0.6 to -0.34) | < 0.001 |
| Saint Kitts and Nevis | 5 (4-6) | 229.11 (189.71-273.5) | 16 (13-20) | 621.27 (491.85-768.59) | 3.33 (1.84 to 4.84) | < 0.001 |
| Saint Lucia | 5 (4-6) | 133.21 (109.74-159.29) | 38 (29-47) | 321.63 (251.14-402.83) | 2.76 (1.48 to 4.05) | < 0.001 |
| Saint Vincent and the Grenadines | 12 (10-14) | 318.16 (264.06-377.96) | 40 (32-49) | 681 (545.95-830.08) | 2.65 (1.65 to 3.66) | < 0.001 |
| Samoa | 14 (8-25) | 441.63 (240.39-797.64) | 21 (12-36) | 399.99 (232.32-670.15) | -0.34 (-0.47 to -0.22) | < 0.001 |
| San Marino | 2 (1-3) | 74.66 (38.86-134.15) | 4 (2-6) | 67.14 (34.98-119.13) | -0.39 (-0.9 to 0.11) | 0.126 |
| Sao Tome and Principe | 11 (6-19) | 378.23 (198.93-633.66) | 18 (8-33) | 475.76 (205.04-855.93) | 0.75 (0.64 to 0.86) | < 0.001 |
| Saudi Arabia | 1083 (597-1999) | 659.71 (361.75-1223.57) | 2694 (1534-4182) | 906.63 (507.88-1403.8) | 1.03 (0.69 to 1.36) | < 0.001 |
| Senegal | 289 (153-477) | 257.72 (134.48-424.62) | 662 (308-1262) | 237.06 (108.99-451.24) | -0.29 (-0.59 to 0) | 0.05 |
| Serbia | 550 (320-906) | 124.59 (72.49-206.07) | 942 (531-1442) | 99.5 (56.1-152.19) | -0.66 (-1.02 to -0.31) | < 0.001 |
| Seychelles | 12 (7-24) | 375.99 (210.11-772.35) | 50 (26-78) | 1030.15 (542.9-1590.34) | 3.16 (2.42 to 3.91) | < 0.001 |
| Sierra Leone | 161 (81-278) | 209.8 (104.35-361.19) | 239 (116-450) | 183.31 (87.11-345.88) | -0.43 (-0.59 to -0.28) | < 0.001 |
| Singapore | 1034 (856-1217) | 1163.4 (959.31-1370.62) | 3964 (3048-4833) | 936.93 (726.39-1141.04) | -0.79 (-1.5 to -0.07) | 0.031 |
| Slovakia | 1003 (660-1476) | 305.35 (200.65-449.9) | 1346 (875-1974) | 238.54 (155.04-349.75) | -0.95 (-1.31 to -0.58) | < 0.001 |
| Slovenia | 404 (314-506) | 282.9 (219.02-355.43) | 723 (493-1026) | 257.51 (175.5-367.83) | -0.33 (-1.13 to 0.47) | 0.418 |
| Solomon Islands | 9 (4-18) | 290.66 (140.56-680.6) | 29 (15-60) | 306.75 (161.81-725.71) | 0.16 (0.05 to 0.26) | 0.003 |
| Somalia | 588 (325-971) | 850.52 (465.65-1423.29) | 2184 (1191-3678) | 951.5 (514.36-1595.48) | 0.37 (0.31 to 0.44) | < 0.001 |
| South Africa | 816 (437-1288) | 89.93 (48.21-142.53) | 2477 (1463-4495) | 119.74 (69.94-217.7) | 0.95 (0.51 to 1.4) | < 0.001 |
| South Sudan | 641 (353-1056) | 768.71 (421.48-1271.43) | 1189 (605-1922) | 1078.14 (547.54-1750.44) | 1.11 (0.99 to 1.22) | < 0.001 |
| Spain | 7668 (6280-9029) | 247.27 (202.03-291.31) | 39924 (29272-49454) | 512.92 (380.91-636.57) | 2.54 (1.99 to 3.09) | < 0.001 |
| Sri Lanka | 933 (406-1885) | 263.66 (113.25-532.24) | 4233 (1900-7801) | 317.79 (141.45-587.37) | 0.6 (0.1 to 1.1) | 0.019 |
| Sudan | 587 (298-1516) | 183.97 (92.74-483.12) | 1372 (763-2476) | 244.18 (136.49-443.74) | 0.92 (0.82 to 1.01) | < 0.001 |
| Suriname | 32 (19-54) | 313.84 (191.2-533.85) | 164 (90-268) | 564.75 (308.62-921.07) | 2.02 (1.57 to 2.47) | < 0.001 |
| Sweden | 2905 (2398-3385) | 296.06 (244.82-344.65) | 3042 (2325-3723) | 210.72 (163.05-257.82) | -1.07 (-1.91 to -0.22) | 0.014 |
| Switzerland | 718 (578-864) | 107.33 (86.57-129.18) | 2875 (2068-3606) | 220.71 (161.72-277.3) | 2.43 (1.03 to 3.86) | 0.001 |
| Syrian Arab Republic | 1816 (1099-3282) | 1148.94 (696.63-2071.18) | 3806 (2156-5985) | 1070.82 (573.26-1718.5) | -0.24 (-0.85 to 0.37) | 0.434 |
| Taiwan (Province of China) | 2397 (2017-2840) | 466.26 (389.36-552.79) | 23858 (18219-29419) | 1059.52 (815.27-1303.57) | 2.61 (1.85 to 3.38) | < 0.001 |
| Tajikistan | 360 (228-583) | 290.77 (184.23-472.25) | 627 (381-967) | 344.76 (209.03-532.38) | 0.56 (0.19 to 0.93) | 0.003 |
| Thailand | 6748 (2531-14373) | 503.8 (186.36-1088.98) | 54024 (29664-85043) | 979.59 (542.08-1538.1) | 2.12 (1.77 to 2.48) | < 0.001 |
| Timor-Leste | 24 (13-44) | 328.33 (170.83-600.93) | 117 (66-196) | 336.68 (186.55-566.99) | 0.07 (-0.06 to 0.21) | 0.283 |
| Togo | 89 (46-153) | 218.51 (112.01-373.73) | 287 (134-536) | 212.03 (96.86-396.31) | -0.11 (-0.2 to -0.03) | 0.011 |
| Tokelau | 0 (0-0) | 337.38 (179.8-658.98) | 0 (0-0) | 315.28 (161.21-656.89) | -0.2 (-0.31 to -0.09) | < 0.001 |
| Tonga | 2 (1-4) | 122.16 (70.42-198.99) | 5 (3-9) | 141.74 (80.95-230.73) | 0.52 (0.08 to 0.95) | 0.019 |
| Trinidad and Tobago | 55 (45-65) | 159.37 (132.74-190.15) | 348 (258-449) | 370.21 (275.49-478.44) | 2.87 (1.44 to 4.32) | < 0.001 |
| Tunisia | 312 (178-626) | 196.91 (111.43-403.52) | 1371 (760-2450) | 247.77 (137.09-447.41) | 0.78 (0.6 to 0.97) | < 0.001 |
| Turkey | 2660 (1458-5707) | 202.43 (110.69-436.4) | 10281 (6503-15373) | 246.58 (155.61-369.1) | 0.79 (-0.22 to 1.82) | 0.127 |
| Turkmenistan | 155 (132-181) | 179.82 (152.4-210.06) | 1273 (963-1653) | 765.4 (582.17-992.37) | 4.95 (3.27 to 6.66) | < 0.001 |
| Tuvalu | 1 (0-2) | 350.49 (186.14-705.47) | 1 (1-3) | 343.21 (190.05-639.8) | -0.06 (-0.11 to -0.02) | 0.008 |
| Uganda | 1278 (676-2170) | 532.17 (279.84-908.38) | 4314 (2503-6991) | 750.18 (433.98-1214.98) | 1.13 (0.88 to 1.37) | < 0.001 |
| Ukraine | 7854 (6644-9179) | 178.19 (150.65-208.26) | 8740 (6000-12322) | 181.48 (124.5-256.07) | 0 (-1.42 to 1.45) | 0.997 |
| United Arab Emirates | 41 (22-78) | 430.92 (227.92-820.02) | 262 (152-404) | 1641.82 (875.37-2595.58) | 4.55 (1.87 to 7.29) | 0.001 |
| United Kingdom | 9387 (8430-10012) | 163.91 (147.36-174.77) | 42811 (34958-47610) | 510.03 (423.14-565.23) | 4.01 (3.13 to 4.91) | < 0.001 |
| United Republic of Tanzania | 3268 (1839-5219) | 807.76 (453.77-1297.38) | 8361 (4943-13007) | 885.91 (519.87-1378.08) | 0.32 (0.16 to 0.48) | < 0.001 |
| United States of America | 84434 (71969-91673) | 423.34 (362.69-458.9) | 160198 (132676-178438) | 475.09 (398.03-527.6) | 0.37 (-0.15 to 0.9) | 0.165 |
| United States Virgin Islands | 15 (9-23) | 483.36 (293.09-745.55) | 34 (20-55) | 346.67 (206.68-567.45) | -1.13 (-1.5 to -0.75) | < 0.001 |
| Uruguay | 372 (311-439) | 172.76 (144.16-203.88) | 5129 (4033-6123) | 1305.7 (1037.92-1557.03) | 6.94 (5.34 to 8.56) | < 0.001 |
| Uzbekistan | 709 (592-842) | 129.38 (107.77-153.73) | 1843 (1443-2300) | 184.51 (143.88-231.43) | 1.19 (-1.73 to 4.19) | 0.428 |
| Vanuatu | 4 (2-8) | 271.75 (142.89-511.71) | 14 (8-25) | 274.58 (154.42-519.56) | 0.02 (-0.09 to 0.14) | 0.689 |
| Venezuela (Bolivarian Republic of) | 349 (288-418) | 91.11 (75-109.14) | 3822 (2826-4961) | 271.32 (201.29-351.45) | 3.55 (2.47 to 4.63) | < 0.001 |
| Viet Nam | 4374 (2115-7634) | 221.99 (107.69-387.43) | 10861 (4961-18272) | 245.61 (112.39-412.92) | 0.35 (0.27 to 0.43) | < 0.001 |
| Yemen | 268 (131-685) | 154.58 (74.98-405.75) | 865 (452-1896) | 191.12 (99.98-424.47) | 0.7 (0.55 to 0.84) | < 0.001 |
| Zambia | 636 (381-986) | 743.8 (444.96-1156.26) | 1899 (1077-2975) | 782.81 (446.1-1229.28) | 0.16 (-0.09 to 0.41) | 0.205 |
| Zimbabwe | 130 (40-241) | 90.77 (27.82-170.33) | 289 (88-559) | 114.51 (33.56-228.41) | 0.84 (0.32 to 1.37) | 0.002 |

Abbreviations: DALYs, disability-adjusted life years; AAPC, average annual percentage change.
